# A Review on the Ethnopharmacology and Phytochemistry of the Neotropical Sages (*Salvia* Subgenus *Calosphace*; Lamiaceae) Emphasizing Mexican Species

**DOI:** 10.3389/fphar.2022.867892

**Published:** 2022-04-19

**Authors:** Nancy Ortiz-Mendoza, Eva Aguirre-Hernández, Itzi Fragoso-Martínez, María Eva González-Trujano, Francisco A. Basurto-Peña, Martha J. Martínez-Gordillo

**Affiliations:** ^1^ Laboratorio de Productos Naturales, Departamento de Ecología y Recursos Naturales, Facultad de Ciencias, Universidad Nacional Autónoma de México, Mexico City, Mexico; ^2^ Flora de Veracruz, Secretaría Académica, Instituto de Ecología, A.C, Xalapa, Mexico; ^3^ Laboratorio de Neurofarmacología de Productos Naturales, Dirección de Investigaciones en Neurociencias del Instituto Nacional de Psiquiatría Ramón de la Fuente Muñiz, Mexico City, Mexico; ^4^ Jardin Botánico, Instituto de Biología, Universidad Nacional Autónoma de México, Ciudad Universitaria, Mexico City, Mexico; ^5^ Departamento de Biología Comparada, Herbario de la Facultad de Ciencias, Facultad de Ciencias, Universidad Nacional Autónoma de México, Mexico City, Mexico

**Keywords:** abietane, bioactive compounds, clerodane, diterpene, mexican traditional medicine

## Abstract

*Salvia* is the most diverse genus within the mint family (Lamiaceae), many of its species are well-known due to their medicinal and culinary uses. Most of the ethnopharmacological and phytochemical studies on *Salvia* are centred on species from the European and Asian clades. However, studies about the most diverse clade, the Neotropical sages (*Salvia* subgenus *Calosphace*; 587 spp.), are relatively scarce. This review aims to compile the information on the traditional medicinal uses, pharmacological and phytochemistry properties of the Neotropical sages. To do so, we carried out a comprehensive review of the articles available in different online databases published from the past to 2022 (i.e., PubMed, Scopus, and Web of Science, among others) and summarized the information in tables. To uncover phylogenetic patterns in the distribution of four different groups of metabolites (mono-, sesqui-, di-, and triterpenes), we generated presence-absence matrices and plotted the tip states over a dated phylogeny of *Salvia*. We found several studies involving Mexican species of *Salvia*, but only a few about taxa from other diversity centres. The main traditional uses of the Mexican species of *Calosphace* are medicinal and ceremonial. In traditional medicine 56 species are used to treat diseases from 17 categories according to the WHO, plus cultural-bound syndromes. Pharmacological studies reveal a wide range of biological properties (e.g., antinociceptive, anti-inflammatory, anxiolytic, cytotoxic, and antidiabetic, etc.) found in extracts and isolated compounds of 38 Neotropical sages. From extracts of these species, at least 109 compounds have been isolated, identified and evaluated pharmacologically; 73 of these compounds are clerodanes, 21 abietanes, six flavonoids, five sesquiterpenoids, and four triterpenoids. The most characteristic metabolites found in the Neotropical sages are the diterpenes, particularly clerodanes (e.g., Amarisolide A, Tilifodiolide), that are found almost exclusively in this group. The Neotropical sages are a promising resource in the production of herbal medication, but studies that corroborate the properties that have been attributed to them in traditional medicine are scarce. Research of these metabolites guided by the phylogenies is recommended, since closely related species tend to share the presence of similar compounds and thus similar medicinal properties.

## 1 Introduction

The Lamiaceae family is the sixth most diverse family within the flowering plants, with 241 genera and 7,530 species widely distributed in the world (Christenhusz and Byng, 2016). Many of its species are well-known because of their important uses for human activities (Harley et al., 2004; Mint Evolutionary Genomics Consortium, 2018). They have culinary value, for example *Ocimum* spp. (Purushothaman et al., 2018); medicinal properties such as *Salvia officinalis* L. (Ghorbani and Esmaeilizadeh, 2017; Nishino et al., 2021), *S. miltiorrhiza* Bunge (Su et al., 2015) and *Scutellaria baicalensis* Georgi (Zhao et al., 2016), and they are used in different industries, such as the cosmetic (e.g., *Pogostemon cablin* (White) Benth., (Van Beek and Joulain, 2017)), and alimentary industries (e.g., *S. hispanica* L. (Muñoz et al., 2013; Hernández-Pérez et al., 2020)). Thus, it is of growing interest in the study of the phytochemistry and pharmacological properties of the members of this family.

The Nepetoideae is the most diverse subfamily of Lamiaceae with 33 genera and 3,685 species (Gul et al., 2019). It includes genera of great chemical diversity, particularly of essential oils (Hegnauer, 1989; Lawrence, 1992; Basset, 2000). The most diverse genus of this subfamily and of the entire family is *Salvia*, which is valued for its medicinal properties, particularly *S. officinalis* L., a prominent species since ancient times. The name *Salvia* comes from the Latin “*salvare*”, which means “to save”, a name given to the genus due to the medicinal properties attributed to it. From an economic point of view, the species of the genus are an important group that is widely known as ornamental, culinary, and medicinal. The species of *Salvia* are considered valuable due to their antiviral (Tada et al., 1994), anti-Alzheimer (Akhondzadeh et al., 2003), anti-inflammatory (Baricevic et al., 2001), antidiarrheal and antispasmodic (Khan et al., 2011), and antimicrobial (Khalil and Zheng-Guo, 2011) activities, among others. The presence of a wide range of bioactive constituents—terpenoids and phenolic compounds—is considered responsible for their pharmacological activities.

*Salvia* is a highly diverse genus with ca. 1,000 species (Etminan et al., 2018), that has been considered a natural group for a long time, due to the presence of a peculiar androecium structure, which exhibits two stamens with elongated connectives forming a structure known as a staminal lever (Claßen-Bockhoff et al., 2003; Claßen-Bockhoff et al., 2004; Walker and Sytsma, 2007). This structure, which is involved in the pollination mechanisms of the genus, has been considered a key innovation and synapomorphy for the group (Claßen-Bockhoff et al., 2003; Wester and Claßen-Bockhoff, 2007). However, since the rise of molecular studies, the genus was demonstrated to be a paraphyletic (Walker et al., 2004; Li et al., 2013), thus it was proposed to expand its circumscription to include other five genera that were nested within *Salvia*: *Dorystaechas* Boiss and Heldr. Ex Benth., *Meriandra* Benth., *Perovskia* Karel., *Rosmarinus* L., and *Zhumeria* Rech and Wendelbo (Drew et al., 2017; Hu G. R. et al., 2018). Alternatively, it was proposed to segregate different lineages of *Salvia* into several genera (Will and Claßen-Bockoff, 2017). However, the expanded circumscription of the genus has been widely accepted due to its practicality.

**FIGURE 1:**
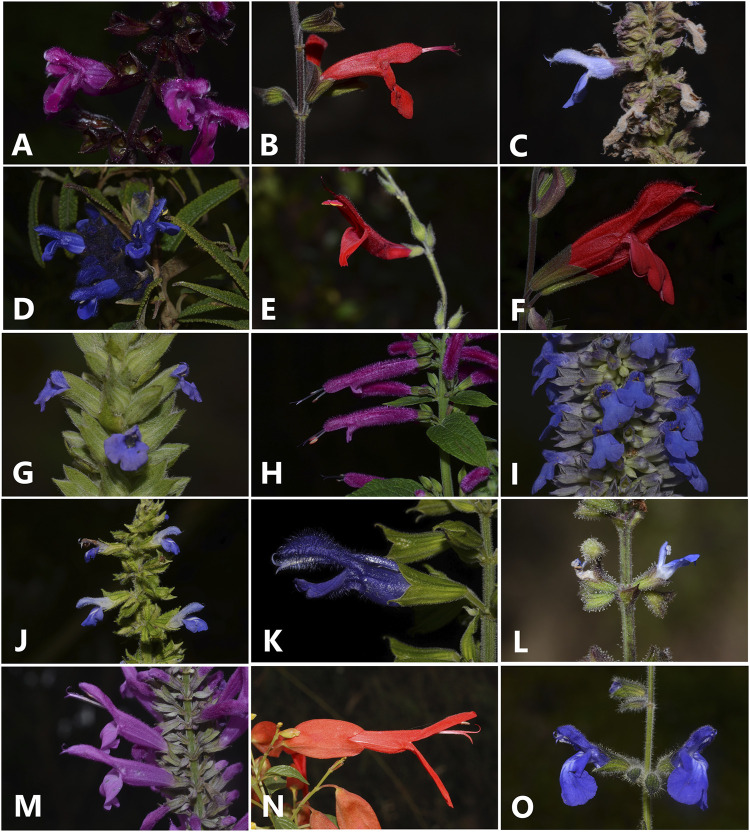
Selected species of Neotropical sages (*Salvia* subgenus *Calosphace*) that have been studied from phytochemical and/or ethnopharmacological perspectives. **(A)** *Salvia carnea*, **(B)** *S. coccinea*, **(C)** *S. connivens*, **(D)** *S. corrugata*, **(E)** *S. elegans*, **(F)** *S. gesneriiflora*, **(G)** *S. hispanica* (chia), **(H)** *S. iodantha*, **(I)** *S. lavanduloides*, **(J)** *S. longispicata*, **(K)** *S. mexicana*, **(L)** *S. occidentalis*, **(M)** *S. purpurea*, **(N)** *S. sessei*, **(O)** *S. urica*. Photo credits Gerardo Salazar.

Currently, *Salvia* is subdivided into 11 subgenera (Drew et al., 2017; Hu G. R. et al., 2018; Kriebel et al., 2019). *Salvia* subgenus *Calosphace* is the most diverse subgenus of *Salvia,* including about half of the species of the genus (587 spp, González-Gallegos et al., 2020; Figure 1). It is endemic to America (Ramamoorthy and Elliot, 1993; Froissart, 2008; González-Gallegos et al., 2020), and its species are mainly distributed in the Neotropics (Jenks et al., 2013; Fragoso-Martínez et al., 2018). Mexico is the main centre of diversification of *Calosphace*, harbouring 295 species. Secondary centres are found in the Andean region and the Antilles, and the most outstanding endemism level is found in Mexico (82%), followed by Brazil (72%), Peru (63%), and Colombia (61%) (González-Gallegos et al., 2020). *Calosphace* is monophyletic and was originally subdivided into several sections by Epling (1939). However, only 12 of these sections have proven to be monophyletic (Jenks et al., 2013; Fragoso-Martínez et al., 2018). Most of the species that have been sampled to date are included in the core *Calosphace* clade, a group that likely resulted from an evolutionary radiation within the subgenus and that encompasses highly morphological diverse taxa from all the diversity centres of the subgenus (Fragoso-Martínez et al., 2018).

The species of *Calosphace* are generally recognized by the presence of a calyx with an entire or tridentate upper lip, a corolla with the upper lip straight and the lower lip patent, the corolla tube lacking a ring of trichomes and, the androecium with the connective of the two stamens fully fused on the posterior region, and the posterior thecae sterile or absent (Bentham, 1848; Epling, 1939). Some of the Mexican species are well known due to their documented nutritional properties (i.e., *S. hispanica* L.; Cahill, 2003; Mohd Ali et al., 2012; Ullah et al., 2016) or for their psychoactive properties that are of pharmacological interest due to their antinociceptive effects (i.e., *Salvia divinorum* Epling and Jativa; Jenks et al., 2011; Tlacomulco-Flores et al., 2020). The most commonly reported bioactive constituents within the subgenus have been characterized as diterpenoids—abietanes and clerodanes—(Bautista et al., 2016; Jiang et al., 2016; Bautista et al., 2017; Esquivel et al., 2017; Campos-Xolalpa et al., 2021).

Despite the high species diversity and economic importance of subgenus *Calosphace,* the most comprehensive ethnopharmacological and phytochemical studies in *Salvia* have been conducted on Asian and European species. The aims of this review are the following: 1) to compile the ethnobotanical knowledge of subgenus *Calosphace* in Mexico, 2) to integrate the pharmacological studies that have been carried out for the species of this subgenus as a promising resource for health resources as antimicrobial, antidiabetic, cytotoxic, anti-inflammatory, and antinociceptive, among other activities, and 3) to summarize the bioactive metabolites characterized within *Calosphace*, in order to explore if their distribution within the subgenus shows a phylogenetic pattern.

## 2 Materials and Methods

### 2.1 Literature Review

The species considered for this review were selected from those correctly identified and reported to belong to subgenus *Calosphace* according to the checklists of Martínez-Gordillo et al. (2017) and González-Gallegos et al. (2020). Then accordingly, the ethnopharmacology and phytochemistry studies were searched in scientific databases of several platforms and editorials such as Google, Google Scholar, PubMed, Elsevier, Science Direct, Springer, Wiley online library, Taylor and Francis, ACS, and RSC using keywords like *Salvia*, *Calosphace*, and more specific terms such as epithets (e.g., *Salvia adenophora*, *S. assurgens, and S. urica,* etc.) which were also combined with other words such as pharmacological, ethnobotany, chemistry, terpenoids. Depending on the topic searched, this review includes literature from 1923 to 2022. All the literature found in these systems was classified, systematized, and organized in tables (Supplementary Tables S1, S2). The disease classifications from the ethnobotanical information followed those recommended by the WHO (WHO. ICD-11, 2021). From the information compiled in the tables, data matrices were generated to classify it. To avoid the use of synonyms in chemical nomenclature or the trivial names of molecules we verified the correct names of the chemical compounds using the digital databases of the National Institute of Standards and Technology (NIST) and of the National Center for Biotechnology Information (NCBI).

### 2.2 Plot of the Differential Distribution of Metabolites in *Salvia*

To uncover phylogenetic patterns on the metabolite distribution of *Salvia* subgenus *Calosphace,* we plotted the presence or absence of the main metabolites found in the subgenus. To do so, we constructed presence-absence matrices that included the 90 species of the Neotropical sages surveyed in this study and 29 species from other *Salvia* s.l. lineages (Supplementary Table S3). For each species, the presence of the following metabolites was scored: mono-, di-, tri-, and sesquiterpenes. The diterpenes were further classified into abietanes and clerodanes and their presence in the surveyed species was scored too. The dated phylogeny of *Salvia* s.l. from Kriebel et al. (2019) was used as a phylogenetic framework to plot the metabolite distribution in the Neotropical sages.

We compared and matched the tips of the phylogenetic tree with the species included in the data matrices, the tips belonging to species that lacked data were pruned using the “drop.tip” function in the *ape* package (Paradis and Schliep, 2019) in R (R Core Team, 2021). The presence-absence data was plotted over a pruned phylogeny containing 102 taxa: 74 species from subgenus *Calosphace*, and 28 representative species of other clades of *Salvia* s.l. (i.e., *Audibertia, Glutinaria, “Heterosphace”, Rosmarinus, Salvia, Salvia aegyptiaca,* and *Sclarea*). To create the figure that summarizes the states at the tips of the trees of multiple metabolites, we used the function “plotTree.datamatrix” in the *phytools* package (Revell, 2012) in R.

## 3 Results

### 3.1 Traditional Uses of the Neotropical Sages

*Salvia* is a genus with a long record of traditional use not only in America but also around the world. Among the most widely known and traditionally used species in the Old World are *Salvia officinalis*, from the Mediterranean, and *Salvia miltiorrhiza*, from China (Chun-Yan et al., 2015; Ghorbani and Esmaeilizadeh, 2017). In America, the species of subgenus *Calosphace* have been used since the pre-Columbian era mainly in Mexico, which is their center of diversity. Two Mexican species stand out for their importance for the pre-Hispanic cultures: *Salvia hispanica* (Figure 1G), known as *Chia*, whose nutritional, ceremonial, and medicinal properties were recognized long before the arrival of the Spanish and were later described in codexes and the chronicles narrated by the conquerors. The diviner’s sage or *S. divinorum* is another widely known species with pre-Columbine use. This species was used by the Mazatec in rituals and later became fashionable as a recreational drug throughout the world (Diaz, 2013). Many of the metabolites of the diviner’s sage remain unknown, and therapeutic properties for the treatment of diseases, particularly neurological ones, have begun to be studied (e.g., Simón-Arceo et al., 2017; Tlacomulco-Flores et al., 2020).

From the literature revision, we identified 56 species of *Calosphace* that have different uses in Mexico. The main use of these species is medicinal, but some of them are also used as substitutes for common household items such as brooms (*S. mexicana* L., Bello et al., 2015, Figure 1K; *S. chamaedryoides* Cav., Solano and Blancas, 2018). The flowers of *S. cinnabarina* M. Martens and Galeotti are used to dye yarns to brown or marron tones (Naranjo, 2012). *Salvia hispanica* (Chia) has been used as a food source since the pre-Columbian era by the Aztecs (Cahill, 2003). Other species whose fruits are consumed are *S. mexicana* and *S. polystachia* Cav. (Bello et al., 2015). Stems and flowers are other organs that are also consumed in certain regions of Mexico (*S. fulgens* Cav., Cornejo et al., 2008). Despite the beauty and variety of colors of the flowers of the Neotropical sages (Figure 1), only a handful of species are used as ornamentals: *S. elegans* Cav. (Cornejo et al., 2008, Figure 1E), *S. leucantha* Cav. (Villavicencio and Pérez, 2002), and *S. splendens* Sellow ex Wied-Neuw. (Martínez et al., 1995). A less frequent use of these species is cosmetic, for instance, the red flowers of *S. coccinea* Buc’hoz ex Etl. are used as blush (Jenks and Seung-Chul, 2013, Figure 1B), while infusions of *S. lavanduloides* Kunth (Figure 1I) and *S. polystachia* are used to wash the hair, darken it, stimulate growth, and prevent loss (Martínez, 1975; Navarro and Avendaño, 2002). Finally, some species have ceremonial use, like the diviner’s sage for the Mazatec (*S. divinorum*, Diaz, 2013). In catholic ceremonies *S. gesneriflora* Lindl. and Paxton (Figure 1F), *S. mocinoi* Benth., *S. purpurea* Cav., (Figure 1M) and *S. thyrsiflora* Benth., are used to decorate religious images and altars (Bello et al., 2015).

**FIGURE 2:**
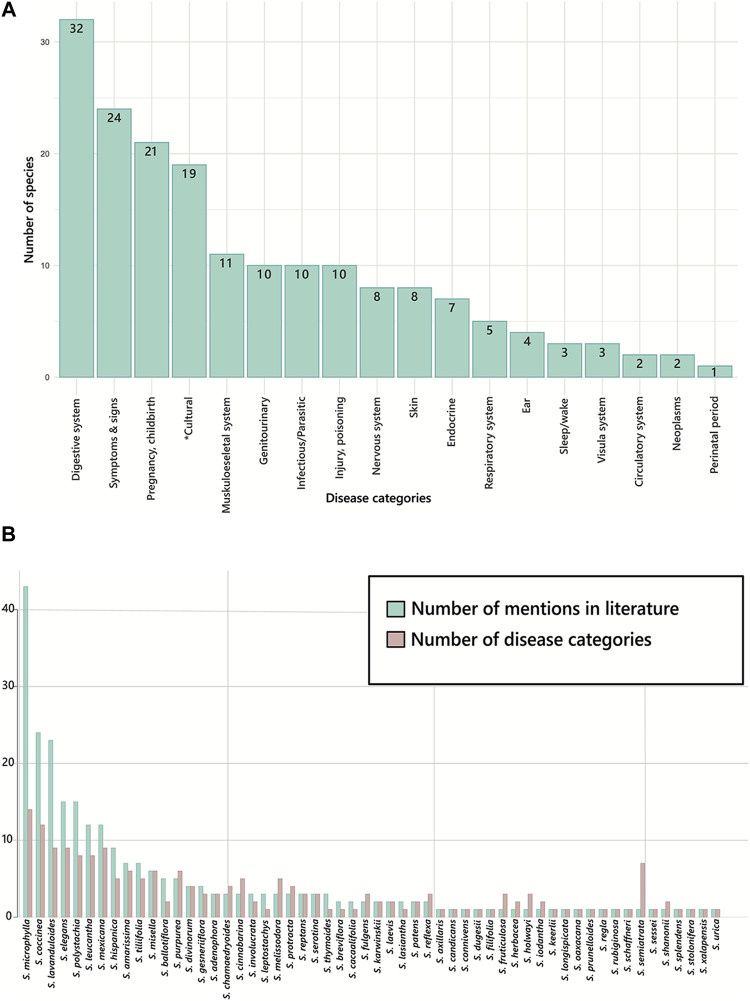
Medicinal uses reported for species of *Salvia* subgenus *Calosphace* in Mexico. **(A)** Number of species used to tackle diseases from different categories according to the WHO (WHO. ICD-11, 2021), **(B)** Number of mentions and disease categories associated with different species of Neotropical sages in Mexican traditional medicine. Abbreviations of the disease categories are as follows: Digestive system (diseases of the digestive system); symptoms and signs (Symptoms, signs or clinical findings, not elsewhere classified); pregnancy, childbirth (Pregnancy, childbirth or the puerperium); *cultural (Cultural-bound syndromes, not included in the WHO classification); musculoskeletal system (Diseases of the musculoskeletal system or connective tissue); infectious/Parasitic (Certain infectious or parasitic diseases); genitourinary (Diseases of the genitourinary system); injury, poisoning (Injury, poisoning or certain other consequences of external causes); nervous system (Diseases of the nervous system); skin (Diseases of the skin); endocrine (Endocrine, nutritional or metabolic diseases); respiratory system (Diseases of the respiratory system); ear (Diseases of the ear or mastoid process); sleep/wake (Sleep-wake disorders); visual system (Diseases of the visual system); neoplasms (Neoplasms); circulatory system (Diseases of the circulatory system); perinatal period (Certain conditions originating in the perinatal period).

Despite the diversity of the subgenus in America, and particularly in Mexico as its main distribution centre*,* science popularization and scientific information about the properties of the species of Neotropical sages are scarce. There are reports of 56 species of *Calosphace* with medicinal use; however, only for 48 of these species there is specific information on the ailments they are used for (Supplementary Table S1). These species are used to treat diseases from 17 categories according to the WHO classification (WHO. ICD-11, 2021), and cultural-bound syndromes. The reported species are mainly used to cure diseases of the digestive system (32 spp.), followed by symptoms, signs or clinical findings (24 spp.), pregnancy, childbirth, and puerperium (21 spp.), and culture-bound syndromes (19 spp.; Figure 2A). The species that was used to treat diseases of most of the categories was *S. microphylla* Kunth (14 categories, Figure 2B), followed by *S. coccinea* (12), *S. lavanduloides, S. elegans,* and *S. mexicana* (9 each).

### 3.2 Pharmacological Activities in *Salvia* Subgenus *Calosphace*

Different studies reveal that the Neotropical sages exhibit a wide range of pharmacological properties. For instance, *S. divinorum* and its active compound salvinorin A have been widely evaluated (e.g., Valdés et al., 2001; Bigham et al., 2003; Lee et al., 2005) using experimental models, such as *in vitro* (lipopolysaccharide-stimulated macrophages, binding affinity opioid receptors, among others), *in vivo* (antidepressant and anxiolytic-like effects, antinociceptive, and to mention a few), *ex vivo* (presynaptic modulator, inhibited twitch contractions, and among others) and in the clinic (i.e., elevated blood cortisol levels) (Casselman et al., 2014).

This section describes the main biological activities reported for species of subgenus *Calosphace* distributed in Mexico (Supplementary Table S2). Table 1 summarizes the biological activities of species that are widely mentioned in Mexican traditional medicine but have been scarcely studied from the pharmacological perspective.

**TABLE 1:** Pharmacological activities for species of *Salvia* subgenus *Calosphace* from Mexico.

Species (Scientific name)	Extract or compound(s)	Part of the Plant	Pharmacological Activities	Positive Control	References
*S. coccinea* Buc’hoz ex Etl.	Aqueous extract	Leaves	DPPH: 80% inhibition at 160 μg/ml	Ascorbic acid	Sudaramoorthy et al. (2021)
			Anti-diabetic. Albino Wistar rats (150–200 g)	Glibenclamide (10 mg/kg, p.o.)	
*S. leucantha* Cav.	Salvileucantholide	Aerial parts	HCT116 (IC_50_ = 32.61 μM); BT474 (IC_50_ = 25.02 μM); HepG2 (IC_50_ = 37.35 μM)	Hsp90 luciferase refolding	Jiang et al. (2016)
	Salvileucantholide; 3β-methoxyisopuberulin; dugesin B		Acetylcholinesterase inhibitory activity (IC_50_ = 50.55; 32.2; 22.13 μM)	n.i	
	Leucansalvialin G and J		Neurotrophic activities on PC12 cells (Differentiation rate 9.52%)	Nerve Growth Factor (Differentiation rate 18.43%)	Li et al. (2018)
	Salvileucalin B		Cytotoxic activity against A549 and HT-29 cells with IC_50_ 5.23 and 1.88 μg/ml, respectively	n.i	Aoyagi et al. (2008)
	Essential oil		Inhibitory activity of the enzyme butyrylcholinesterase (IC_50_ = 32.60 μg/ml)	Donepezil (IC_50_ 3.6 μg/ml)	Villalta et al. (2021)
	Salviandulin E		Antitrypanosomal activity against *Trypanosoma brucei* (IC_50_ 0.72 μg/ml)	Pentamidine (IC_50_ 0.0017 μg/ml), suramin (IC_50_ 1.58 μg/ml), eflornithine (IC_50_ 2.27 μg/ml)	Aoyagi et al. (2014)
*S. mexicana* L.	Aqueous extract	Aerial parts	*Staphylococcus aureus* (MIC/MBC 1.19/1.19 mg/ml). *S. epidermidis* (MIC/MBC 4.75/9.50 mg/ml). *Escherichia coli*, *Pseudomonas aeruginosa* (MIC/MBC 9.50/9.50 mg/ml)	Nisin: *S. aureus*, *S. epidermidis* (0.63/0.63 mg/ml). *E. coli* (0.50/1.0 mg/ml). *P. aeruginosa* (1.0/1.0 mg/ml)	Afonso et al. (2019a)
			DPPH, ferric reducing power, TBARS (EC_50_ = 10.0; 34.0; 26.2 μg/ml)	Ascorbic acid, butylated hydroxy anisole and trolox (EC_50_ = 6.68; 16.1; 23.0 μg/ml)	
			Antiinflammatory activity: NO production inhibition	Dexamethasone (EC_50_ = 66.3 μg/ml)	
			HepG2 (EC_50_ = 52.4 μg/ml); HeLa (EC_50_ = 61.0 μg/ml); MCF-7 (EC_50=_66.2 μg/ml)	Ellipticine (EC_50_ = 1.0; 2.0; 1.0 μg/ml)	
*S. microphylla* Kunth	Carnosic acid 12-methylether	Aerial parts	Antibacterial activity (*S. aureus* 78 μg/ml)	Cefuroxime sodium	Aydoǧmuş et al. (2006)
	Microphyllandiolide; salvimicrophyllin B; salvimicrophyllin D		*E. histolytica* (IC_50_ = 182.2; 172.9; 187.2 µM); *G. lamblia* (IC_50_ = 201.3; 161.4; 215.3 μM)	Metronidazole (IC_50_ = 0.23 µM); (IC_50_ 1.22 µM); Emetine (IC_50_ = 0.83 µM); (IC_50_ 2.18 µM)	Calzada et al. (2015)
	Essential oil	Leaves	Antioxidant activity β-carotene/linoleic acid (IC_50_ = 770 μg/ml)	Thymol (IC_50_ = 714 μg/ml)	Lima et al. (2012)
	Hexane extract	Aerial parts	Insecticidal against *Spodoptera frugiperda* (LC_50_ = 456 ppm)	n.i	Romo-Asunción et al. (2016)
	Ethanol extract (95%)	Leaves	-Neuroprotective effect in the memory impairment evaluated in male albino rats Step-through passive avoidance (300 mg/kg, p.o.), Morris water maze (150 and 300 mg/kg, p.o.)	Donezepil (0.5 mg/kg, i.p.)	Ayoub et al. (2022)
			-Cholinergic dysfunction via acetyl cholinesterase activity (150 and 300 mg/kg, p.o.)		
			-Oxidative stress markers: catalase activity, reduced glutathione level and lipid peroxidation level (300 mg/kg, p.o.)		
*S. polystachia* Cav.	Linearolactone; polystachyne E	Aerial parts	*E. histolytica* (IC_50_ = 22.9; 76.6 µM). *G. lamblia* (IC_50_ = 28.2; 83.6 μM)	Metronidazole (IC_50_ = 0.23; 1.22 µM) Emetine (IC_50_ = 0.83; 2.18 µM)	Calzada et al. (2015)
	Ethanol extract		Neuroprotective: *In vitro* excitotoxicity model (100% protection at 0.1 μg/μl)	Resveratrol	Pineda-Ramírez et al. (2020)
			*In vivo* ischemia model. Male Wistar rats (90.4% at 3 mg/kg, i.v.)		
			Antioxidant. OH (IC_50_ = 154.18 μg/ml); O_2_^−^ (IC_50_ = 118.97 μg/ml); ROO- (IC_50_ = 18.21 μg/ml)	Resveratrol: OH (IC_50_ = 30.02 μg/ml); O2- (IC_50_ = 28.28 μg/ml); ROO (IC_50_ = 18.21 μg/ml)	
	Salvifiline A; 15-epi-salvifiline A	Leaves	Induced collagen (COL1A1) transcription at 0.27 µM. Human dermal fibroblasts	Ascorbic acid (100 µM)	Bautista et al. (2017)
	Polystachyne G; 15-epi-polystachyne G		Stimulated expression of the elastin gene at 25.6 µM		

DPPH, 2,2-diphenyl-1-picrilhidrazil; EC_50_, Half-maximal effective concentration; IC_50_, Half-maximal inhibitory concentration; LC_50_, Half-maximal lethal concentration; MBC, minimal bactericidal concentration; MIC, minimum inhibitory concentration; n.i., non-information; TBARS, thiobarbituric acid reactive substance.

#### 3.2.1 Antibacterial Activity

The large groups of secondary metabolites of plants—mainly phenolics, terpenes, and alkaloids—selectively exert their antibacterial action against different microorganisms through different routes of intervention, such as binding to proteins, for example, adhesins, by enzyme inhibition, substrate deprivation, membrane disruption, metal ion complexation or with cell wall (Cowan, 1999). A few species from *Salvia* have been investigated for their effective antibacterial activity, using the crude extract or the isolated bioactive compounds to compare with a positive control. Examples found in the literature were *S. adenophora* Fernald*, S. albocaerulea* Linden*, S. buchananii* Hedge*, S. chamaedryoides, S. farinacea* Benth.*, S. greggii* A. Gray*, S. mexicana, S. microphylla, S. reptans* Jacq.*, S. sessei* Benth., and *S. urica* Epling (Pereda-Miranda et al., 1992; Martínez-Vázquez et al., 1998; Kawahara et al., 2004; Romero et al., 2005; Aydoǧmuş et al., 2006; Bisio et al., 2015; Gómez-Rivera et al., 2018; Afonso et al., 2019a). The experiments refer to a specific part of the plant used in traditional medicine, reporting mainly aerial parts or leaves and flowers, and rarely roots (Supplementary Table S2). Antibacterial activity of majority of them has been reported against *Staphylococcus aureus* and/or *S. epidermidis*, as well as *Escherichia coli* and *Psedomona aeruginosa.* For *S. chamaedryoides,* several bioactive compounds effective against *Enterococcus faecium* and *E. faecalis* were isolated (Bisio et al., 2017). Compounds from *S. reptans* and *S. greggii* had antibacterial activity against *Bacillus cereus*, *Micrococus luteus* (Martínez-Vázquez et al., 1998), and *Bacillus subtilis* (Kawahara et al., 2004). Whereas the extracts from different polarities of *S. sessei* were effective against *S. haemolyticus, S. hominis,* and *E. faecalis* (Gómez-Rivera et al., 2018) (Supplementary Table S2).

#### 3.2.2 Anticholinesterase Effect

Anticholinesterase drugs can prolong the presence of released acetylcholine in the synapse producing an increase in the muscarinic and nicotinic receptors activities. Thus, these drugs might have an influence on pharmacological activity in neuromuscular junction; cardiovascular, respiratory, and gastrointestinal systems; secretory glands, and in local application in eyes mediated by a medium duration (reversible) or long-acting (irreversible) activity depending on the anticholinesterase drug. Clinical applications involve Myasthenia gravis (an autoimmune disease), Alzheimer’s disease (dementia associated with deficiency of structural intact cholinergic neurons), paralytic ileus and glaucoma, among others (Nair and Hunter, 2004). In *Salvia* subgenus *Calosphace*, the essential oil of *S. buchananii* has antiacetylcholinesterase and antibutyrylcholinesterase effects (Beladjila et al., 2018a). On the other hand, *S. leucantha* has been reported with anticholinesterase activity (Villalta et al., 2021), in part due to the presence of the compounds salvileucantholide (IC_50_ = 50.55 μM) (Table 1), 3β-methoxyisopuberulin (IC_50_ = 32.20 μM) Dugesin B (IC_50_ = 22.13 μM) (Jiang et al., 2016), as well as its essential oil (IC_50_ = 32.60 μg/ml) obtained from aerial parts. (Supplementary Table S2).

#### 3.2.3 Antidepressant and Anxiolytic Effects

Central nervous system (CNS) activity produced by plants is commonly appreciated by people, mainly because of their effects on mood and the belief of them being an efficient and innocuous treatment for diseases. It has been reported greater use of traditional medicine more than 2 fold in individuals affected by a mental disorder (52.9%) compared to people without a mental disorder (22.1%), in depression or anxiety where prevalence is higher in women, being those who use it for self-help more than men. Severity of side effects over the course of treatment with antidepressant or anxiolytic medications is an important factor that causes people to give up pharmacological treatment, mainly those suffering comorbidly, where the time of treatment and response play an important role (Braund et al., 2021). Antidepressant activity is recognized in *Salvia* species from subgenus *Calosphace* such as *S. elegans* (Mora et al., 2006) due to the compound 5-O-(6-rhamnosylglucoside)-7-hydroxy-4′-methoxyflavanone (González-Cortazar et al., 2013) using the forced swimming test. Whereas *S. tiliifolia* Vahl contains *tilifodiolide* as bioactive compound responsible for its antidepressant activity evaluated in the tail suspension test (Alba-Betancourt et al., 2019). A relevant compound with several pharmacological activities is Salvinorin A obtained from *S. divinorum* which not only has antidepressant activity (Calzada et al., 2015), but also anxiolytic-like response was detected in the light-dark test (Herrera-Ruiz et al., 2006). Anxiolytic-like response due to tranquilizing effects has also been reported for *S. cinnabarina* (Maione et al., 2009), *S. elegans* (Mora et al., 2006), and *S. semiatrata* using the elevated plus-maze test. The herbal medication for these two CNS health conditions has not reported severe adverse effects, being recognized as one of the most important advantages of their use (Posadzki et al., 2013). In fact, one of the most common secondary effects in depressant activity of medicinal plants could be the sedative response, which is also useful in CNS diseases such as in the sleep architecture alterations. *Salvia cinnabarina* might exemplify this property by enhancing the sodium pentobarbital hypnotic effects (Maione et al., 2009) (Supplementary Table S2).

#### 3.2.4 Antifungal and Insecticidal Activities

The most common types of antifungal drugs are systemically administered and target different parts of the fungal cell. An example of this kind of antifungal compound is the heptaene amphotericin B that interacts with the major component of the fungal cell membrane called ergosterol (ergosta-5,7,22-trien-3β-ol), modifying the permeability and fluidity of membrane. The most used antifungal families of drugs azoles (miconazol itraconazole, and clotrimazole) or alilamines (terbinafine) also inhibit the ergosterol synthesis. On the other hand, the most selective drugs for fungal cells are the echinocandins, which inhibit the synthesis of the fungal cell wall. In mammalian cells these drugs have reduced adverse effects, compared to those that have been commonly observed with the 5-Flucytosine (5-FC) (e.g., hepatotoxicity, nephrotoxicity, myelotoxicity, and gastrointestinal problems), that interacts with the nucleus of the fungus, altering the biosynthesis of deoxyribonucleic acid (DNA) alone or combined with cytarabine (Houšť et al., 2020). Medicinal preparations of *S. hispanica* have been reported to produce not only antifungal effects such as in the case of its essential oil (Elshafie et al., 2018), but also insecticidal properties (Chen et al., 2021). These preparations have been tested against *Spodoptera frugiperda*, an important and well-known pest in the agricultural field that attacks various crops of economic importance, such as corn and cotton (León-García et al., 2012) (Supplementary Table S2). Regarding insecticidal substances, these have been classified depending on their chemical structure, toxicological effects, and or the mechanism of penetration. For this pharmacological activity, several organic compounds occurring naturally in plants are useful insecticides. Toxic action of them occurs by ingestion when insects inhale or have contact with the poison that penetrates their exoskeleton, or when they bite or chew some parts of the plant. Some species from subgenus *Calosphace* have been reported with these properties mainly by using the essential oils, organic extracts, or even isolated constituents. The essential oil of *S. ballotiflora* Benth., has these properties in part due to the presence of β-caryophyllene (Cárdenas-Ortega et al., 2015), *S. connivens* (Zavala-Sánchez et al., 2013; Figure 1C), *S. keerlii* Benth., (Zavala-Gómez et al., 2021), and *S. microphylla* (Romo-Asunción et al., 2016) also possess these activities (Supplementary Table S2).

#### 3.2.5 Antiprotozoal Activity

Protozoans are responsible for a variety of diseases including malaria (caused by species of *Plasmodium*) and Chagas disease (caused by flagellated protozoan *Trypanosoma cruzi*). Antiprotozoal drugs are agents that kill or inhibit the growth of these organisms. Many of them can cause DNA breakage or prevent its replication, such as metronidazole used for vaginal infections caused by *Trichomonas vaginalis*, but also in the treatment of giardiasis by flagellated amoeba. Other drugs can produce enzymatic inhibition, for example trimethoprim-sulfamethoxazole, which is used to inhibit folic acid synthesis in *Pneumocystis carinii.* However, the chemotherapy for protozoal infections causes adverse effects during therapeutic doses that limit their safety use where combined therapies are considered too (Thurston et al., 2015). At least nine species from *Calosphace* have been investigated as potential options for antiprotozoal therapy the 5,6-dihydroxy-7,3′,4′-trimethoxyflavone was isolated from acetone extract and ethyl acetate fraction of *S. amarissima* Ortega and proved to be effective against *E. histolytica*, IC_50_ = 0.05 μg/ml and *G. lamblia*, IC_50 =_0.13 μg/ml (Calzada and Bautista, 2020). Whereas nuchensin (flavone) from *S. connivens* produced effects at similar potency with an IC_50_ = 0.072 µM and IC_50_ = 0.118 μM, respectively (Bautista et al., 2020). *Salvia divinorum* contains abundant salvinorin A that had effect on the same protozoans at a reported IC_50_ = 49.0 µM and IC_50_ = 64.8 μM, respectively (Calzada et al., 2015) (Supplementary Table S2). Other diterpenoids (clerodanes) with activity against *E. histolytica* and *G. lamblia* have been isolated from *S. herbacea* Benth., *S. microphylla, S. shannonii* Donn. Sm. (Bautista et al., 2013; Calzada et al., 2015). The diterpenoids (abietanes) Clinopodiolide A-C, 19-O-acetylclinopodiolide A, Triacetylclinopodiolide B from *S. clinopodiodies* Kunth exhibit the same activity (Bustos-Brito et al., 2019). The linearolactone from *S. polystachia* had amoebicidal and giardicidal activities at IC_50_ = 22.9 µM and IC_50_ = 28.2 µM (Calzada et al., 2015) (Supplementary Table S2). Finally, the salviandulin E from *S. leucantha* had activity against *Trypanosoma brucei* at IC_50_ 0.72 μg/ml (Aoyagi et al., 2014).

#### 3.2.6 Neuroprotective Activity

Neurodegenerative diseases cause cellular damage that can affect the structure of neurons and/or their function. This damage is in part due to processes of inflammation, oxidative stress, and neurotransmission or hormone-modified conditions that can be regulated by the presence of extracts and their chemical constituents improving the quality of life of people. According to different studies, *Salvia leucantha*, *S. polystachia,* and *S. tiliifolia* are sources of neuroprotective alternatives (Supplementary Table S2). From these species, leucansalvialin G and J from aerial parts of *S. leucantha* (Li et al., 2018) (Table 1), and Tiliifolin E produced this activity in PC12 cells differentiation (Fan et al., 2017). Differentiation of PC12 cells is assessed by semi-quantitative or quantitative morphological methods. These methods can include the measurement of the cell size, neurite number, and neurite length (Hu R. et al., 2018).

#### 3.2.7 Antidiabetic and Antioxidant Activities

The anti-hyperglycemic effects of medicinal plants are considered to improve the function of pancreatic tissue by rising insulin secretion or reducing the intestinal absorption of glucose (Kooti et al., 2016). The ether extract and fraction (Solares-Pascasio et al., 2021), as well as the compounds (Salinas-Arellano et al., 2020) of *S. amarissima, S. chamaedryoides* (Bisio et al., 2017), *S. coccinea* (Sudaramoorthy et al., 2021), and *S. elegans* (Pereira et al., 2018) are examples of species from *Salvia* subgenus *Calosphace* reported as significant antidiabetic plants. For instance, the aerial parts of *S. amarissima* contain in abundance the flavonoid pedalitin, which has been reported to possess antidiabetic activity by the recombinant α-glucosidase with maltase-glucoamylase action (Flores-Bocanegra et al., 2017), and the inhibition of the protein tyrosine phosphatase (PTP-1b) (Salinas-Arellano et al., 2020). The dichloromethane extract and the compound (1R,5S,7S,8S,9R,10R,12R)-1,7,8-trihydroxycleroda-3,13 (16),14-triene-17,12; 18,19-diolide from aerial parts of *S. chamaedryoides* was reported to be inhibitory of a-glucosidase and α-amylase activities (Bisio et al., 2017) (Supplementary Table S2).

Since diabetes is a chronic endocrinological disorder in which the metabolism of proteins, fats, and carbohydrates is altered in the oxidative stress that enhances the reactive oxygen species, the antidiabetic activity of plants could be due to their antioxidant properties. Thus, plants that contain several natural antioxidants from different natures can intervene in the regulation of tissular damage, as it has been corroborated using the essential oil, organic extracts, and/or a few bioactive isolated compounds (Supplementary Table S2). The aqueous and dichloromethane extracts from aerial parts of *S. greggii* (Pereira et al., 2018) and *S. wagneriana* Pol. (Giamperi et al., 2012) and 6,7,11,14-tetrahydro-7-oxo-icetexone isolated from the aerial parts of *S. ballotiflora* (Esquivel et al., 2017), respectively, produced significant antioxidant activity evaluated in DPPH assay. The DPPH assay is complemented by using other tests, such as ferric reducing power (FRAP) and TBARS explored with the hexane, dichloromethane, methanol extracts, and the isolated compounds isosessein and sessein from the aerial parts of *S. sessei* (Gómez-Rivera et al., 2018), and the aqueous extract of the aerial parts of *S. mexicana* (Afonso et al., 2019b).

#### 3.2.8 Antidiarrheal and Antispasmodic Activities

Both diarrhea and spasms can be part of symptoms of several unhealth conditions associated with the gastrointestinal system. For these symptoms, the identification of its aetiology to establish a pharmacological treatment is very important. Antidiarrheal drugs are motility inhibitors, adsorbents, and/or fluid and electrolyte transport modifiers. The most common adverse effects produced by these substances are constipation, nausea, dry mouth, and abdominal pain, whereas serious adverse effects of some products are paralytic ileus, toxic megacolon, and angioneurotic edema. Spasmolitic drugs are a group of substances that prevent or interrupt the painful and involuntary contraction of intestinal smooth muscle named spasm, a mechanism referred to as the genesis of pain in gastrointestinal pathologies. Several substances isolated as natural products have been recognized and used as spasmolytic drugs, many of them associated with direct smooth muscle relaxants such as those agents derived from papaverine. Other substances act as anticholinergics, for example, butylhyoscine, hyoscine, hyoscyamine, levocine, dicycloverine, butylscopolamine, trimebutine and cimetropium bromide, as well as calcium channel blocking agents as pinaverium bromide, otilonium bromide, alverine, fenoverine, rociverine, and pirenzepine. At least three species of Neotropical sages have been reported to possess antidiarrheal properties: the methanol extract of the aerial parts of *S. connivens* (Pérez-Gutiérrez et al., 2014; the isolated compounds 19-deoxyicetexone (Pérez-Gutiérrez et al., 2013) and clinopodiolide A, B, and C, triacetylclinopodiolide B (Bustos-Brito et al., 2019) from *S. ballotiflora* and *S. clinopodiodies*, respectively. Finally, tilifodiolide obtained from aerial parts of *S. tiliifolia* (Alba-Betancourt et al., 2019) and 3,4-secoisopimara-4 (18),7,15-triene-3-oic acid from *S. cinnabarina* have been investigated as spasmolytic treatment (Romussi et al., 2000) (Supplementary Table S2).

#### 3.2.9 Antihypertensive Activity

Hypertension is one of the highest risk factors for cardiovascular accidents, coronary heart disease, cardiac hypertrophy with heart failure (hypertensive heart disease), aortic dissection, and renal failure being a major cause of morbidity and mortality. Hypertension is a disease that very often is not detected and when diagnosed, is not always adequately treated. Anti-hypertensive drugs act by decreasing the cardiac output, the peripheral vascular resistance, or both. The classes of drugs most often used to treat hypertension include the thiazide diuretics, b-blockers, angiotensin-converting enzyme (ECA) inhibitors, angiotensin II receptors antagonists, calcium channel blockers, a-adrenoceptor blockers, combined a- and b-blockers, direct vasodilators, and some centrally acting drugs such as a2-adrenoceptor agonists (Dphil, 2004). In the Neotropical sages hypotensive and antihypertensive effects have been reported in the 3,4-secoisopimara-4 (18),7,15-triene-3-oic acid isolated from aerial parts of *S. cinnabarina* (Alfieri et al., 2007), and the hydroalcoholic extract and fractions from aerial parts of *S. elegans* (Jiménez-Ferrer et al., 2010).

#### 3.2.10 Antinociceptive and Anti-inflammatory Activities

Pain alone or associated with inflammation is a clinical condition present in several pathologies. It could be *per se* a disease, producing disabling conditions and diminished quality of live-in people. Both analgesic and anti-inflammatory drugs are pharmacological armaments for pain relief. Despite these medications being recognized because of their efficacy, the risk of the presence of several adverse effects are limited in their chronic use. Thus, there is a continuous need to develop new efficacious and safety alternatives. Plants have been the origin of several potent and efficacious analgesic compounds for the most important groups of analgesics drugs, such as opiates and non-steroidal anti-inflammatory drugs. To this respect, *Calosphace* species have been reported to possess significant antinociceptive and/or anti-inflammatory activities according to the results obtained in several pain models, mainly induced by thermal and chemical stimuli. Non-polar, medium polar, and polar extracts of the aerial parts of *S. amarissima* (Moreno-Pérez et al., 2019; Fernando et al., 2021) and *S. semiatrata* (Ortiz-Mendoza et al., 2020), as well as from leaves of *S. divinorum* (Tlacomulco-Flores et al., 2020) were explored in the plantar, writhing and/or formalin test (Fragoso-Serrano et al., 2019; Moreno-Pérez et al., 2019). Amarisolide A and pedalitin from *S. amarissima* (Moreno-Pérez et al., 2019; Fernando et al., 2021), salvinorin A from *S. divinorum* (Tlacomulco-Flores et al., 2020), and tilifodiolide from *S. tiliifolia* (González-Chávez et al., 2018) were isolated as the most abundant bioactive constituents using the same nociceptive tests. Carrageenan-induced edema assay was also applied to corroborate the anti-inflammatory properties of the tilifodiolide isolated from *S. tiliifolia* (González-Chávez et al., 2018) and the ethyl acetate extract containing amarisolide A in higher concentration vs. other extracts of the same *Salvia* species (Calzada et al., 2015; Fernando et al., 2021). To corroborate their anti-inflammatory properties the chloroform and dichloromethane extracts from the aerial parts of *S. ballotiflora* (Campos-Xolalpa et al., 2021) and *S. connivens* (González-Chávez et al., 2017), respectively, were explored in the TPA-induced ear edema (Supplementary Table S2).

#### 3.2.11 Cytotoxic Activity

Cytotoxic drugs inhibit or prevent the function of cells, they include the substances used in chemotherapy for cancer treatment. When therapeutic doses are given to patients, cytotoxic drugs produce toxic side effects due to their poor selectivity between the target (i.e., cancerous cells) and normal cells, having mutagenic, teratogenic, and carcinogenic effects in humans, particularly in cancer patients receiving long-term therapy. These adverse effects include neoplasms and leukemias, testicular and ovarian dysfunction—including permanent sterility, cumulative chromosome damage, and other organ damage such as in the cases of the alkylating agents (nitrogen mustards, ethylenimine derivatives, and nitrosoureas). However, not all cytotoxic drugs are carcinogenic. Moreover, the use of combined drugs might be more effective than single agents, reducing the adverse effects. In the case of plants with cytotoxic properties, a synergy among their bioactive constituents is recognized, which makes them a good option for cancer therapy, being effective and safe to use. Several species of *Salvia* subgenus *Calosphace* have been reported to possess cytotoxic activity, for example: *S. amarissima*, *S. anastomosans* Ramamoorthy, *S. ballotiflora, S. buchananii*, S*. leucantha,* and *S. semiatrata* (Supplementary Table S2). The ethyl acetate fraction obtained from the acetone extract of the arterial parts of *S. amarissima* produced cytotoxic activity in HeLa cells with an IC_50_ = 1.50 ± 0.21 μg/ml (Bautista et al., 2016). The isolation of pure compounds showed the same activity but in a reduced potency, as it was noticed for teotihuacanin (IC_50_ = 13.7 ± 4.9 μg/ml) and amarissinin A (IC_50_ = 14.0 ± 1.04 μg/ml) (Bautista et al., 2016). Cytotoxic activity was also reported for cariocal isolated from the roots of *S. anastomosans* (Esquivel et al., 2005). From the chloroform extract of the aerial parts of *S. ballotiflora* antitumoral and cytotoxicity activities were described (Campos-Xolalpa et al., 2017; Campos-Xolalpa et al., 2021), in part due to the presence of 7,20-dihydroanastomosine, 7α-acetoxy-6,7-dihydroicetexone, and anastomosine (Esquivel et al., 2017), as well as 19-deoxyisoicetexone (Campos-Xolalpa et al., 2017). From the roots of *S. buchananii* it was isolated hyptadienic and salvibuchanic acids (Beladjila et al., 2018b), and tilifolidione from *S. semiatrata* (Esquivel et al., 2005). Whereas from the aerial parts of *S. leucantha*, salvileucantholide were evaluated using MTT assay (Jiang et al., 2016) (Table 1), a colorimetric assay to measure cellular metabolic activity as an indicator of cell viability, proliferation, and cytotoxicity. Finally, the aqueous extract from aerial parts of *S. mexicana* was evaluated to confirm cytotoxic activity using HepG2 (hepatocellular carcinoma), HeLa (cervical carcinoma), and MCF-7 (breast carcinoma) (Afonso et al., 2019b) (Supplementary Table S2).

### 3.3 Bioactive Compounds Found in *Salvia* Subgenus *Calosphace*

Phytochemical studies have resulted in the isolation and identification of a wide variety of bioactive secondary metabolites—grouped in terpenoids and phenolic compounds—, from ca. 90 species of Neotropical sages (Esquivel, 2008; Wu et al., 2012). In the following section these groups of constituents are described.

#### 3.3.1 Terpenoids

A number of *in vitro, in vivo,* and *ex vivo* studies, have demonstrated that the terpenoids are the main compounds responsible for the therapeutic activity of the sages (Aydoǧmuş et al., 2006; Pérez-Gutiérrez et al., 2013; Li et al., 2018; Fernando et al., 2021). The active terpenoids that have been isolated from species of *Salvia* subgenus *Calosphace* can be grouped into mono-, sesqui-, di-, and triterpenes. The diterpenes (abietanes and clerodanes) are the most common of these compounds and thus have been more extensively studied, having several pharmacological evaluations and biological activities reported.

##### 3.3.1.1 Monoterpenoids

**FIGURE 3:**
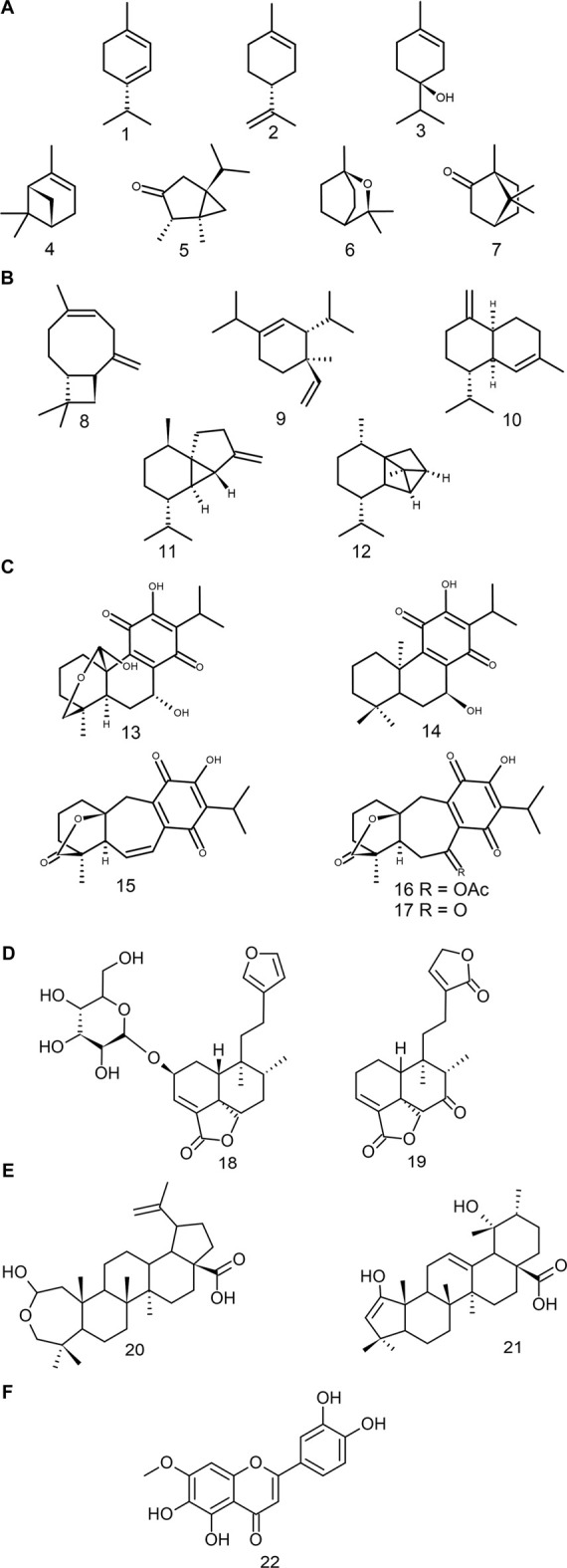
Bioactive compounds found in *Salvia* subgenus *Calosphace*. **(A)** Monoterpenoids, **(B)** Sesquiterpenoids, **(C)** Abietanes, **(D)** Clerodanes, **(E)** Triterpenoids, **(F)** Polyphenols.

Monoterpenoids are widely distributed in land plants and are mostly found in essential oils (Kabir et al., 2020). Studies have demonstrated the insecticidal activity of the essential oils of *S. ballotiflora, S. elegans,* and *S. keerlii* (Cardenas et al., 2015; Mathew and Thoppil, 2011; Zavala-Gómez et al., 2021), the antioxidant potential of *S. microphylla* (Lima et al., 2012), and the antifungal activity and inhibition of the butyrylcholinesterase enzyme of *S. hispanica* and *S. leucantha* (Elshafie et al., 2018; Villalta et al., 2021). Species such as *S. elegans, S. keerlii* and *S. leucantha* have a great diversity of these types of metabolites in their essential oils (De Martino et al., 2010; Rojas et al., 2010; Zavala-Gómez et al., 2021). Some of the most common components of the essential oils from these species belong to the monocyclic type—γ-terpinene (1), limonene (2) and 4-terpineol (3)—, while others are bicyclic (e.g., α-pinene (4), *cis*-thujone (5), 1,8-cineole (6) and camphor (7) (Figure 3A). The essential oils of *S. cinnabarina* and *S. greggii* showed the same diversity of monoterpenoids; however, they lack pharmacological or biological evaluations (Bisio et al., 1998; Giuliani et al., 2017).

##### 3.3.1.2 Sesquiterpenoids

These compounds, as the monoterpenoids do, form a significant part of the essential oils of sages. They constitute the most diverse groups of terpenoids, having lineal, mono-, bi-, tri-, and tetracyclic structures (Ludwiczuk et al., 2017). However, within the Neotropical sages, the cytotoxic effect has only been demonstrated by the insecticidal effect of the β-caryophyllene 8) (Cárdenas-Ortega et al., 2015). The latter is a bicyclic compound common to the species of the genus *Salvia* s.l. (Tenore et al., 2011; Ascrizzi et al., 2017; Borges et al., 2019). A wide diversity of these metabolites can be found in different species of the subgenus, for instance, δ-elemene 9), γ-muurolene 10), β-cubebene 11) and cyclosativene 12) (Figure 3B) have been identified in the essential oil of *Salvia elegans.* Sesquiterpenoids are a potential field of study in pharmacology, due to their anti-inflammatory properties (Da Silveira e Sá Rde et al., 2015).

##### 3.3.1.3 Diterpenoids

These terpenoids are characteristic of *Salvia* and have been proposed as a quimiotaxonomic marker. A number of these metabolites have been characterised from species of subgenus *Calosphace*, they can be classified mainly in abietanes and clerodanes (Esquivel, 2008). In several evaluations of pharmacological and biological activities, it has been demonstrated that the most active extracts are those of median polarity, where these compounds are found in the highest concentration (Ortiz-Mendoza et al., 2020; Tlacomulco-Flores et al., 2020; Fernando et al., 2021).

###### Abietanes

Conacytone (13), horminone (14), and icetexone (15) are all examples of compounds commonly found in species of Neotropical sages (Wu et al., 2012) (Figure 3C). The icetexone and its derivatives (i.e., α-acetoxy-6,7-dihydroicetexone (16) and the 6,7,11,14-tetrahydro-7-oxo-icetexone (17)) have mainly cytotoxic, anti-inflammatory and antioxidant activities (Esquivel et al., 2017). On the other hand, the conacytone and horminone have anti-inflammatory and antibacterial properties (Esquivel et al., 2017; Martínez-Vázquez et al., 1998). Some species that have a wide range of abietanes but lack pharmacological studies are: *S. anastomosans, S. ballotiflora, S. candicans* M. Martens and Galeotti, *S. concolor* Lamb. ex Benth., *S. corrugata* Vahl (Figure 1D), *S. pubescens* Benth., S. *reptans*, and *S. uliginosa* Benth., (Sanchez et al.,. 1989; Cárdenas and Rodríguez-Hahn, 1995; Esquivel et al., 1997; Martínez-Vázquez et al., 1998; Giacomelli et al., 2013; Esquivel et al., 2017; Cezarotto et al., 2019; Díaz-Fernández et al., 2019).

###### Clerodanes

A number of clerodanes have been characterised in the following species of subgenus *Calosphace*: *Salvia amarissima, S. divinorum, S. dugesii* Fernald, *S. hispanica, S. leucantha,* and *S. polystachia* (Shirota et al., 2006; Gang et al., 2011; Jiang et al., 2016; Bautista et al., 2017; Calzada et al., 2020; Fan et al., 2020). Most of these clerodanes are classified, according to their absolute stereochemistry, as *neo*-clerodanes (Li et al., 2016). More than 80 *neo*-clerodanes isolated from Neotropical sages have been evaluated in pharmacological and biological activity studies, corroborating their several properties (Supplementary Table S2). Recent studies have demonstrated that *neo*-clerodanes such as, Amarisolide A (18) and 7-keto-neoclerodan-3,13-dien-18,19:15,16-diolide (19) (Figure 3D), have great antinociceptive and anti-inflammatory potentials (Ortiz-Mendoza et al., 2020; Fernando et al., 2021).

##### 3.3.1.4 Triterpenoids

In this group stand out the oleanolic and ursolic acids, two compounds common to sages from all the subgenus (Wu et al., 2012). Both pentacyclic triterpenoids have several biological effects, such as antimicrobial, anti-inflammatory, anti-ulcer, antihyperlipidemic, hepatoprotective, and hypoglycemic effects (Topçu, 2006). Within *Calosphace*, two compounds that are derived from these acids have been isolated: hyptadienic acid (20) and the salvibuchanic acid (21) (Figure 3E). Both compounds have a cytotoxic effect against Jurkat and HeLa cell lines (Beladjila et al., 2018b).

##### 3.3.1.5 Polyphenols

These metabolites are notorious for their potent antioxidant effect, and recently activity associated with chronic illness prevention has been detected (Ziaullah and Rupasinghe, 2015). This group of compounds is characterised by the presence of hydroxyl groups (-OH) in their structures, which gives them an affinity for high-polarity solvents. In high-polarity extracts from species of subgenus *Calosphace* antidiabetic, antioxidant, antibacterial, antinociceptive and anxiolytic effects have been reported (Supplementary Table S2). However, pharmacological evaluations of the isolated compounds from this group are scarce, and only six of them have been studied, being the Pedalitine (22) the most cited (Figure 3F) (Flores-Bocanegra et al., 2017; Moreno-Pérez et al., 2019; Salinas-Arellano et al., 2020).

#### 3.4 Differential Distribution of Terpenoids in *Salvia* Subgenus *Calosphace*

**FIGURE 4:**
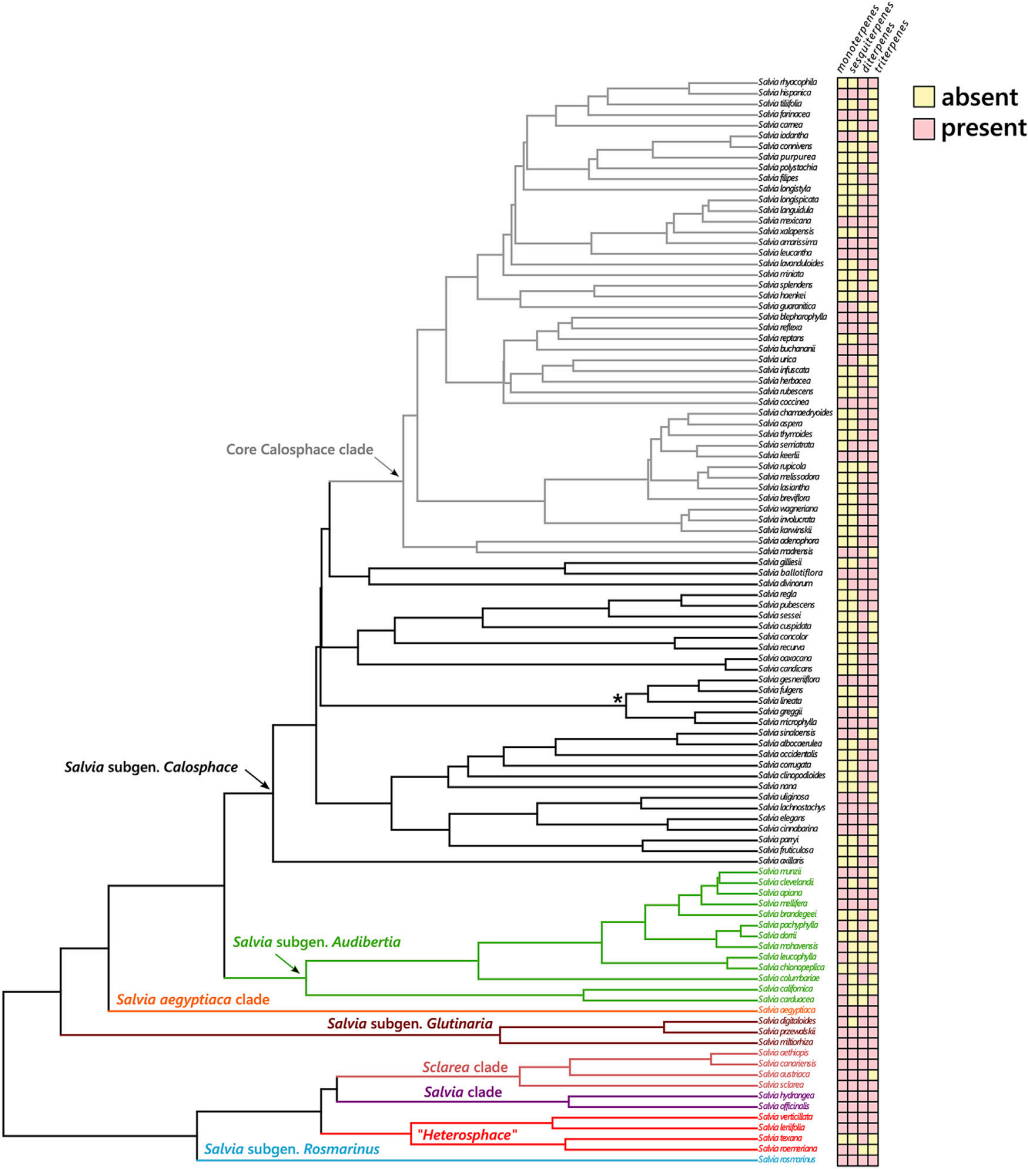
Distribution of different types of terpenes in the Neotropical sages (*Salvia* subgen. *Calosphace*) and their related lineages. The lineages of *Salvia* s.l. are color-coded. An asterisk (*) marks the Fulgentes subclade, recovered as part of the core Calosphace clade in other phylogenies (i.e., Jenks et al., 2013; Fragoso-Martínez et al., 2018).

The most characteristic metabolites found in *Salvia* are the diterpenoids and there is no clear pattern on their distribution throughout the phylogeny (Figure 4). However, when the diterpenoids are further classified into abietanes and clerodanes, there is a marked difference in their distribution along the lineages of *Salvia* (Supplementary Figure S1). The abietanes are mostly found in the European, Asian, and North American lineages of *Salvia* (i.e., subgenus *Audibertia, Glutinaria, Rosmarinus,* and the clades *Salvia, Sclarea* and *Salvia aegyptiaca*). Within *Salvia* subgenus *Calosphace*, these metabolites are mainly present in members of the early diverging lineages, and are absent mostly in the members of the most diverse clade, core Calosphace. On the other hand, the clerodanes are restricted to the Neotropical sages, and they are mostly found in the members of the core Calosphace clade (Supplementary Figure S1).

Regarding the distribution of the remaining terpenoids in the subgenus, the second most common metabolites found in *Salvia* subgenus *Calosphace* were the triterpenoids. However, they were also common in the remaining lineages of *Salvia*, although they have been scarcely reported for the Californian sages (*Salvia* subgenus *Audibertia*), which are the sister lineage of the Neotropical sages (Figure 4). According to the phytochemical studies reviewed, the sesquiterpenes are the third most abundant metabolite in subgenus *Calosphace*. They were present in the other lineages of *Salvia* as well, and they were usually produced by the same species that present monoterpenes (Figure 4). Nevertheless, this pattern was not observed in *S.* subgenus *Audibertia*, probably due to the scarcity of phytochemical studies that identified them. Lastly, the monoterpenes seem to be the least abundant metabolites in subgenus *Calosphace.* Only 26% of the surveyed species have reports of monoterpenoids, being apparently more widely distributed in the other lineages of *Salvia* (86% of studies from the included species).

## 4 Discussion

*Salvia* subgenus *Calosphace* is the most diverse lineage of the genus, and it is mainly distributed in the Neotropics. In Mexico, *Salvia* is the most diverse genus of flowering plants, which explains its central paper in traditional medicine, being used by many of the ethnic groups found in the country to treat a wide variety of diseases. The most employed species are *S. microphylla*, *S. coccinea,* and *S. lavanduloides,* all of them native and widely distributed species, but none of them endemic to Mexico. The main uses attributed to the Mexican species are those related to the digestive system, followed by symptoms and signs (e.g., fever, headaches), pregnancy and childbirth, and cultural-bound syndromes. Despite the diversity of traditional uses reported for the different species of Neotropical sages, many are yet to be explored and corroborated by scientific studies. For instance, the properties of *S. lavanduloides*, which is used to treat diseases from 9 categories, have not been investigated in any pharmacological study. Reviews such as the one presented here can encourage the study of the medicinal properties and pharmacological potential of the native sages.

Regarding the pharmacological studies of species from subgenus *Calosphace*, literature revision allowed us to notice that the investigation of their potential is scarce. The extracts or isolated compounds of 38 species of subgenus *Calosphace* have been studied, from these ca. 109 chemical structures have been evaluated, being the diterpenes with clerodane- and abietane-type skeleton the most common with 73 and 21 structures, respectively. The few species that have been explored in preclinical studies have shown a wide spectrum of biological activities. Most of the studied species were referred to as potential antioxidant, anti-inflammatory and cytotoxic, followed by the group of plants used for their activities against microorganisms and insects (antibacterial > insecticidal > antiprotozoal effects). It is worth noting that the extracts of some species produced important effects on the central nervous system, including those species used as anxiolytic > antinociceptive > antidepressant. Finally, few species are reported to be effective for gastrointestinal and cardiovascular systems and some others have influence on metabolic illnesses.

The anti-inflammatory, antioxidant, and/or cytotoxic properties reported for independent species or even a single one, suggests their potential use for difficult conditions such as cancer therapy. It is well-known that free radicals contribute to protein and DNA damage, inflammation, tissue damage, and subsequent cellular apoptosis, which is thought to play a role in the development of cancer and neurodegenerative diseases (Uttara et al., 2009). Thus, antioxidant properties of plants and their natural products can exert protective effects to this and others health conditions, due to its remarkable activity against reactive oxygen species and other free radicals, a property mainly recognized for polyphenols, nitrogen compounds, terpenoids, and vitamins (Uttara et al., 2009; Nani et al., 2021). Anti-inflammatory effects are also an important property of plants, since several alterations in health at peripheral, and central level begin with the presence of inflammation. Chronic inflammation has been associated to be involved in tumorigenesis (i.e., in cellular transformation, promotion, survival, proliferation, invasion, angiogenesis, and metastasis), as well as in degenerative diseases such as Alzheimer, Parkinson, Multiple Sclerosis, and Huntington’s disease (Amor et al., 2010; Singh et al., 2019). According to our literature search, other mental conditions such as anxiety, depression, and sleep alterations, can also be impacted by several extracts of Neotropical sages.

The most characteristic and studied metabolites in the Neotropical sages are the diterpenoids (clerodanes and abietanes). Studies focusing on the pharmacological properties of other metabolites such as monoterpenes, sesquiterpenes, triterpenes, and phenolic compounds should be expanded to provide a more complete picture of the medicinal potential of *Salvia* subgenus *Calosphace*.

The members of *Salvia* subgenus *Calosphace* are rich in bioactive compounds, but many of them are shared with their sister lineages (e.g., mono-, tri-, and sesquiterpenoids) or even with other groups of land plants (e.g., the oleanolic and ursolic acids). However, the diterpenoids, particularly the clerodanes, are almost exclusive to the Neotropical sages. One of such clerodanes is the Amarisolide A, found in *Salvia amarissima*, that has been studied for its analgesic, anti-inflammatory, antidiabetic, anxiolytic, and cytotoxic properties. An increased study of members of *Calosphace* from other centres of diversity (i.e., Andean region, Antilles, and Brazil) is needed to further understand the evolution of the phytochemistry of the Neotropical sages and uncover finer biogeographic, ecological, karyological, phylogenetic, and/or morphological patterns that could potentially be associated with the distribution of the terpenoids in *Calosphace*.

## 5 Perspectives

Ethnobotanical, pharmacological, and phytochemical information integrated into this review gives evidence that the Neotropical sages are a source of possible new drugs for acute and chronic diseases that will support chemical weaponry of medicine which always requires novel, effective and safe options for the treatment of diseases. We recommend that future studies targeting specific compounds at finer scales use the phylogenies available for the group to guide their sampling, focusing on sister species or taxa from the same clades that are likely to share some of the compounds of interest.
