# Supplementary material for: A Review on the Ethnopharmacology and Phytochemistry of the Neotropical Sages (Salvia Subgenus Calosphace; Lamiaceae) Emphasizing Mexican Species
Source: Front Pharmacol. 2022 Apr 19;13:867892. doi: 10.3389/fphar.2022.867892 (PMC9061990; doi:10.3389/fphar.2022.867892)
Supplement: Supplementary file 2 [file Table1.docx]

**Supplementary Table 1.** Traditional uses reported for species of *Salvia* subgenus *Calosphace* from México sorted according to the disease classification of the WHO (ICM-11, 2021).

| **Species** | **Vernacular name** | **State (Mexico)*** | **Ailment categories**  **ICD WHO 2019*** | **Part of the plant, preparation and/or administration** | **Reference** |
| --- | --- | --- | --- | --- | --- |
| ***S. adenophora* Fernald** | Salvia roja de Oaxaca | Oax | Skin, Culture-bound syndromes (“limpias”) | Poultice  Bundle of branches | Bisio et al., 2015 |
|  | ND | Oax | Skin | ND | Cruz-Pérez et al., 2021 |
|  | Mirto | Hgo | Digestive system | Aerial parts, Infusion, Beverage | Aguilar et al., 1996 |
| ***S. amarissima* Ortega** | Bretónica | Oax | Endocrine disease | Leaves, Decoction or crushed in water, Beverage | Cervantes, 1979 |
|  | Chan | Mich | Skin | Aerial parts, Decoction, Washing | Esquivel et al., 2018 |
|  | Bretónica | Oax | Endocrine disease | Leaves | Castro et al., 2014 |
|  | ND | ND | Culture-bound syndromes (“aire”, “susto”, “mal de ojo”) | Bundle of branches | García et al., 2014 |
|  | ND | Oax | Endocrine disease  Digestive system | ND | Cruz-Pérez et al., 2021 |
|  | Nadri (ñhañhu) | Mex | Musculoskeletal system | Aerial parts, Crushed, Topical | Aguilar et al., 1996 |
|  | Hierba del cáncer, hierba del golpe | Mor | Neoplasms | Decoction | Monroy and Castillo, 2000 |
| ***S. axillaris* Moc. and Sessé ex Benth.** | Hierba del buche | Jal | Digestive system | Aerial parts, Cecoction, Beverage | López, 1994 |
| **S*. ballotiflora***  **Benth** | Engordacabra, crespa, mejorana, mejorana del país | Chih, Zac, Hgo, Tam. | ND | ND | Standley, 1923 |
|  | engordacabra, mejorana | Dgo | Digestive system, Pregnancy, childbirth, puerperium | ND | González et al., 2004 |
|  | Peistón | NL | Digestive system | Aerial parts | González, 2010 |
|  | ND | ND | Digestive system | Aerial parts, Decoction | Domínguez et al., 1976 |
|  | Mejorana | Mex | Pregnancy, childbirth, puerperium | Aerial parts, Decoction, Baths | Campos et al., 2021 |
| ***S. breviflora* Moc. and Sessé ex Benth.** | Salvia | Mor | Digestive system | Leaves | Maldonado, 1997 |
|  | Salvia | Mor | Digestive system | ND | Monroy and Castillo, 2000 |
| ***S. cacaliifolia* Benth.** | ND | Chis | Pregnancy, childbirth, puerperium | Steam cure | Breedlove and Laughlin, 1993 |
|  | Cacalia sage | Chis | Pregnancy, childbirth, puerperium | Aerial parts, Decoction | Domínguez and Castro 2002 |
| ***S. candicans* M. Martens and Galeotti** | ND | Oax | ND | ND | Cruz-Pérez et al., 2021 |
| ***S. chamaedroydes* Cav.** | Mirto | Pue | Digestive system | ND | Senties, 1984 |
|  | Chía | SLP | Symptoms and signs | Decoction, Baths | Solano and Blancas, 2018 |
|  | Salvia | Mor | Pregnancy, childbirth, puerperium, Conditions related to sexual health | ND | Monroy and Castillo, 2000 |
| ***S. cinnabarina* M. Martens and Galeotti** | Tzajalpomtz´unun | Chis | Diseases of the digestive system | Aerial parts | De la Cruz et al., 2014 |
|  | ND | Chis | Musculoskeletal system, Pregnancy, childbirth, puerperium, Symptoms and signs, Culture bound syndromes (“llanto”) | Aerial parts | Domínguez and Castro, 2002 |
|  | ND | Oax | ND | ND | Cruz-Pérez et al., 2021 |
| ***S. coccinea* Buc’hoz ex Etl*.*** | Mirto, salvia | Chis, Nay, NL, Tam, Yuc | ND | ND | Standlley, 1923 |
|  | Oromento, Chachaknich (Chol) | Chis | Symptoms and signs, Culture-bound syndromes (“espanto” and “vergüenza”) | Aerial parts, Crushed, on the head or spreads on the chest (heart)  Sun-dried leaves, Decoction, Beverage | Cahuich et al., 2014 |
|  | Tziktzil (mayan) | Yuc | Culture-bound syndromes (“mal de ojo”) | Leaves and flowers, Decoction, Beverage and Baths | Cahuich et al., 2014 |
|  | Mirto | Hgo | Digestive system | Decoction, Beverage | Andrade, 2009 |
|  | Mirto rojo | Ver | Visual system | Flowers, Decoction, Baths | Domínguez et al., 2015 |
|  | Mirto | Tam | Ears | Leaves, Crushed in warm water, Topical in ears | Macouzet et al., 2013 |
|  | Mirto | Mor | Digestive system, Culture-bound syndromes (“susto”, “mal de ojo”), Nervous system | Leaves and flowers | Maldonado, 1997 |
|  | Mirto | NL | Digestive system, Musculoskeletal system, Visual system | Leaves and seeds. | González, 2010 |
|  | Mirto rojo | Hgo | Genitourinary system, Digestive system, Infectious diseases | Flowers, Decoction, Washes, or Beverage | Espinosa, 1985 |
|  | Mirto, koral, hut’ut’ wits (teenek) | SLP | Digestive system, Infectious diseases | Roots or leaves, Decoction, Beverage | Alcorn, 1984 |
|  | Mirto rojo | Mor, Tab | Digestive system, Genitourinary system, Skin, Nervous system, Musculoskeletal system. | Aerial parts and roots, Decoction, Cataplasm, Bath | Argueta, 1994 |
|  | x-p´oklampiix | Yuc | Culture-bound syndromes (“aire”, “susto”, “mal de ojo”) | Bunch of plants | Jenks and Seung-Chul, 2013 |
|  | ND | NL | Culture-bound syndromes (“ik viento”), Symptoms and signs, Visual system | Aerial parts, Decoction  Wet seeds on the eye | Jenks and Seung-Chul, 2013 |
|  | Flor de colibrí, vitzikixoóhitl, mirto | Ver | Culture-bound syndromes (“mal aire”) | Bunch of branches. | Navarro and Avendaño, 2002 |
|  | Mirto | ND | Digestive system, Circulatory system, Culture-bound syndromes (“limpias”) | Aerial parts, Whole plants, Decoction  Bunch of branches, Sweeps | Zamora and Nieto, 1992 |
|  | Betónica | NL | Symptoms and signs, Visual system | Stems, Decoction  Seeds over eyes | Estrada et al., 2007 |
|  | ND | Oax | ND | ND | Cruz-Pérez et al., 2021 |
|  | Chaktsits (mayan), chaktsitsil (mayan) tsabits (mayan) | Q.Roo | ND | ND | Pulido and Serralta, 1993 |
|  | Sajanic, huamal | Chis | Digestive system, Infectious diseases | Aerial parts, Decoction, Beverage | Aguilar et al., 1996 |
|  | Tlachinolxochitl | SLP | Visual system | ND | Aguilar et al., 1996 |
|  | Mirto | Ver | Digestive system, Infectious diseases | Leaves, Decoction | Del Amo, 1979 |
|  | Chamamalhtuwat (totonaco) | Pue | Pregnancy, childbirth, puerperium, Culture-bound syndromes (“susto en niños”) | Aerial parts, Decoction, Beverage and Baths | Guerrero, 2020 |
|  | Bils (mayan) | Yuc | Digestive system, Infectious diseases | Aerial parts, Decoction, Beverage | Ku, 2018 |
|  | Mirto | Mor | Digestive system, Nervous system, Culture-bound syndromes (“mal de ojo”, “susto”) | Aerial parts, Placed under the pillow | Monroy and Castillo, 2000 |
| ***S. connivens* Epling** | ND | Gto | Digestive system | Aerial parts | Calzada and Bautista, 2020 |
| ***S. divinorum* Epling and Játiva** | Ska Pastora | Oax | ND | Aerial parts | Díaz, 2013 |
|  | Hierba de ka pastora, ska pastora | Oax | Digestive system, Infectious diseases | Leaves | Calzada and Bautista, 2020 |
|  | Pastora | Oax | Symptoms and signs | Leaves, Decoction, Beverage | Romero, 2018 |
|  | Ska Pastora, la María, Ska María Pastora | Oax | Symptoms and signs, Infectious diseases, Culture-bound syndromes (“panzón de borrego”) | ND | Valdés et al., 1983 |
| ***S. dugesii* Fernald** | Chía cimarrona | Gto, Qro, Mex | ND | ND | Standley, 1923 |
| ***S. elegans* Vahl** | Uop yooxi’ (Tepehuano) | Dgo | Symptoms and signs | Seeds | González et al., 2004 |
|  | Mirto de campo | Hgo | Nervous system, sleep-wake disorders | Aerial parts, Placing under the pillow | Villavicencio and Pérez, 2002 |
|  | Mirto rojo | Hgo | Nervous system, sleep-wake disorders | ND | Molina et al., 2012 |
|  | Mirto | Mex | Digestive system | ND | Monroy 2016 |
|  | Limoncillo | Mich | Digestive system | ND | Bello and Salgado, 2007 |
|  | Mirto de campo | Hgo | Nervous system, Sleep-wake disorders | Aerial parts, Decoction, Beverage and/or Placing under pillow | Martínez, 2007 |
|  | Mirto | Pue | Pregnancy, childbirth, puerperium | Flowers, Decoction, Bath | Chino and Jacques, 1986 |
|  | Hierba del burro, mirto de flor roja, mirto inglés, mirto macho, salvia, toronjil de monte | Mex, CDMX | Digestive system, Nervous system, Sleep-wake disorders, Symptoms and sings, Culture-bound syndromes (“aire”, “espanto”), Pregnancy, childbirth, puerperium | Leaves, Aerial parts and roots, Decoction, Beverage and Poultice | Argueta, 1994 |
|  | ND | Hgo | Nervous system | Aerial parts, Decoction, Beverage or Baths | Aguilar et al., 1996 |
|  | mirto macho | CDMX | Nervous system, Sleep-wake disorders | Aerial parts | Aguilar et al., 1996 |
|  | jetcho deni (otomí) | Mex | Respiratory system | ND | Aguilar et al., 1996 |
|  | Lyé zii (zapotec) | Oax | ND | Plant | Luna and Rendón, 2008 |
|  | Mirto rojo | ND | Ears | Leaves, Poultice | Zamora and Nieto, 1992 |
|  | Salvia | Mex | Symptoms and signs | Poultice, Topical | Mendoza, 1883 |
|  | Mirto, tonaltixochitl | Ver | Infectious diseases | Poultice | Aguilar et al., 1996 |
| ***S. filifolia***  **Ramamoorthy** |  | Mich | Digestive system | ND | Hurtado and Rodríguez, 2006 |
| ***S. fruticulosa* Benth.** | ND | Oax | Culture-bound syndromes, Endocrine diseases, Pregnancy, childbirth, puerperium | ND | Cruz-Pérez et al., 2021 |
| ***S. fulgens* Cav.** | Mirto | Dgo | ND | ND | González et al. 2004 |
|  | Pinyesi (Mazahua), Mirto macho | Mex | Nervous system, Sleep-wake disorders, Symptoms and signs | Aerial parts and flowers, Decoction, Topical or steam Inhalation | Argueta, 1994 |
| ***S. gesneriiflora* Lindl. and Paxton** | Cadenilla | Dgo | Injury, Symptoms and signs |  | González et al., 2004 |
|  | Huitzihzilxochitl | Mex | Digestive system, Injury | Aerial parts, Decoction, Beverage | Cazares, 1994 |
|  | Flor de colibrí | ND | Digestive system, Parasitic diseases | Leaves | Calzada and Bautista, 2020 |
|  | Mirto | Mor | Digestive system | Decoction | Monroy and Castillo, 2000 |
| ***S. herbacea* Benth.** | ND | ND | Digestive system, Parasitic diseases | Leaves | Calzada and Bautista, 2020 |
| ***S. hispanica* L.** | Chía, bayalh (Tepehuano) | Dgo | Musculoskeletal system | Leaves, Roasted, Topical | González et al., 2004 |
|  | Chía | Mich | Musculoskeletal system | Leaves, Roasted, Topical | Toledo, 2014 |
|  | Chía | Mex | Symptoms and signs | Aerial parts, Scrubbed | Jiménez, 1994 |
|  | Chía, chian | Mich, Gro | Injury (remove spider larvae from eyes) | Seeds on the eyes | Soto, 1987 |
|  | Chía | Mex | Pregnancy, childbirth, puerperium | ND | White et al., 2013 |
|  | Chía | ND | Digestive system | Ingested like pills | Alonso et al., 2015 |
|  | Salvia, Chía | NL | Digestive system | Aerial parts, Decoction, Beverage | Estrada et al., 2007 |
|  | ND | Oax | ND | ND | Cruz-Pérez et al., 2021 |
|  | Chía, salvia | Son | Digestive system | Leaves, Seeds | López and Hinojosa, 1988 |
| ***S. holwayi* S.F. Blake** | Salvia | Chis | Symptoms and signs, Cultural-bound syndromes (“llanto”), Pregnancy, childbirth, puerperium | Aerial parts | Domínguez and Castro, 2002 |
| ***S. involucrata* Cav.** | Mirto real | Pue | Cultural-bound syndromes (“limpias”) | Aerial parts | Chino and Jacques, 1986 |
|  | Mirto real | Pue | Cultural-bound syndromes (“aire”) | Leaves | Jenks and Seung-Chul, 2013 |
|  | Mirto real | Pue | Pregnancy, childbirth, puerperium | Decoction | Aguilar et al., 1996 |
| ***S. iodantha* Fernald** | Hierba de la virgen | Mich | Pregnancy, childbirth, puerperium, Skin | Aerial parts, Decoction, Baths | Aburto, 2013 |
| ***S. karwinskii* Benth.** | Salvia | Chis | Digestive system | Aerial parts | Domínguez and Castro, 2002 |
|  | Xoma (mazateco) | Oax | Pregnancy, childbirth, puerperium | Aerial parts, Decoction, Beverage | Romero, 2018 |
| ***S. kerlii* Benth.** | Sake | SLP | Symptoms and signs (bruises or twist) | Aerial parts | Serrano et al., 2020 |
| ***S. laevis* Benth.** | Salvia real cimarrona | Ver | Pregnancy, childbirth, puerperium | Aerial parts | Navarro and Avendaño, 2002 |
|  | Palmita | Mex | Genitourinary system | Leaves, Decoction, Beverage | Aguilar et al., 1996 |
| ***S. lasiantha* Benth.** | ND | Chis | Cultural-bound syndromes (“pérdida del alma”) | ND | Breedlove and Laughlin, 1993 |
|  | Salvia | Chis | Cultural-bound síndrome (“pérdida del espíritu”) | Aerial parts | Domínguez and Castro, 2002 |
| ***S. lavanduloides* Kunth** | Cantuesa | Mex | Respiratory system, Injury | Decoction | Heras and Ariza, 2007 |
|  | Poleo | Dgo | Respiratory system, Pregnancy, childbirth, puerperium, Skin, Digestive system | Decoction | González et al., 2004 |
|  | Guikilludunee (cuicateco) | Oax | Digestive system | Aerial parts, Decoction or Alcoholic maceration, Beverage | Mercado, 2013 |
|  | Cordón de cristo, cantahueso, poleo | Mich | Cultural-bound syndromes (“empacho”), Pregnancy, childbirth, puerperium | Aerial parts, Decoction, Beverage | Toledo, 2014 |
|  | Lucema | Oax | Respiratory system | Leaves and flowers, Decoction with salt, Beverage | Martínez, 2016 |
|  | Lucema | Mich | Digestive system, Pregnancy, childbirth, puerperium, Symptoms and signs | ND | Bello and Salgado, 2007 |
|  | Hierba de la carbonera, itamo, cantueso, alucema | Méx | Digestive system, Respiratory system | Aerial parts, Decoction, Beverage | Jiménez, 1994 |
|  | Alucema | Mich, Gro | Pregnancy, childbirth, puerperium | Aerial parts, Decoction, Beverage or Baths | Soto, 1987 |
|  | Cantahuesos | Mex | Digestive system | Aerial parts, Decoction, Beverage | Cazares, 1994 |
|  | Flor de locema, alucema, koj rechuun (triqui) | Oax | Digestive system, Endocrine diseases, Genitourinary system | Aerial parts, Decoction, Beverage | Naranjo, 2012 |
|  | Esculcona | Mich | Genitourinary system | Flowers and leaves, Decoction, Beverage. | Aburto, 2013 |
|  | Flor de olote, re’e itha (me’phaa) | Gro | Digestive system, Pregnancy, childbirth, puerperium, Symptoms and signs, Cultural-bound syndrome (“mal aire”) | Flowers, Decoction, Beverage | Arellano, 2017 |
|  | Salvia | Gro | Respiratory system | Leaves, Raw, Chewed | Solano, 2008 |
|  | ND | Chis | Respiratory system, Symptoms and signs | ND | Breedlove and Laughlin, 1993 |
|  | Cuetehton (náhuatl) | Mor | Digestive system | Aerial parts, Decoction | Argueta, 1994 |
|  | Baka nich momol (tzeltal/tzotzil),  Torongiz | Chis | Pregnancy, childbirth, puerperium, Respiratory system, Cultural-bound syndrome (“aire”) | Flowers and leaves, Decoction  Branches | Argueta, 1994 |
|  | La rla (zapoteco), hierba del coraje | Oax | ND | Leaves | Luna and Rendón, 2008 |
|  | Lucema, mazorquilla, alucema, aguanda-tsitsiki, chía cimarrona, flor del cielo, k’uironi simarroni | Mich | Digestive system, Pregnancy, childbirth, puerperium, Symptoms and signs | Aerial parts and flowers, Decoction, Beverage or Bath, Sweeps | Bello et al., 2015 |
|  | ND | Oax | Skin | ND | Cruz-Pérez et al., 2021 |
|  | Cantueso | Mex | Skin | Aerial parts, Decoction, Washes | Martínez, 1975 |
|  | Bacal nichz, Chabacal, nish vomol (tzotzil) | Chis | Respiratory system | Leaves, Decoction, Beverage | Aguilar et al., 1996 |
|  | Lucuma | Mich | Digestive system | Aerial parts, Decoction, Beverage | Aguilar et al., 1996 |
|  | Cante, cantueza, cuatehto, salvia morada | Mor | Cultural-bound syndrome (“aire”, “frialdad”), Digestive system and, Respiratory system, Symptoms and signs, Injury | Aerial parts, Decoction, Beverage  Bunches of branches, Sweeps with rubbing alcohol | Monroy and Castillo, 2000 |
| ***S. leptostachys* Benth.** | Chía | Mor | Cultural-bound syndromes (“aire en los oídos”) | Leaves | Maldonado, 1997 |
|  | Chia, chan | Mich, Gro | Cultural-bound syndromes (“aire en los oídos”) | Leaves, Heated and with alcohol, Topical | Soto, 1987 |
|  | Chía | Mor | Cultural-bound syndromes (“aire en los oídos”) | Leaves | Monroy and Castillo, 2000 |
| ***S. leucantha* Cav.** | Cordoncillo | Hgo | Respiratory system | Flowers, Decoction | Villavicencio and Pérez, 2002 |
|  | Salvia morada | Mex | Respiratory system, Nervous system, Endocrine diseases | Aerial parts, Decoction, Beverage | White et al., 2013 |
|  | Salvia real, salvaría, alucema, planta del bálsamo, salvia na noó (mazahua). | Mex | Digestive system, Cultural-bound syndrome (“aire”), Symptoms and signs, Pregnancy, childbirth, puerperium. | Aerial parts, Decoction, Beverage or Baths  Leaves, chewed, Topical (ears) | Lozano, 1996 |
|  | Algodoncillo |  | Pregnancy, childbirth, puerperium | Aerial parts, Decoction, Beverage | Chino and Jacques, 1986 |
|  | Cordón de San Francisco | Hgo | Digestive system | ND | Flores, 1998 |
|  | Salvia morada | ND | Endrocrine diseases | Aerial parts, Decoction | Andrade and Heinrich, 2005 |
|  | Salvia morada | Gro | Digestive system, Symptoms and signs, Cultural-bound syndrome (“mal aire”) | Leaves, Decoction, Beverage | Juárez et al. 2013 |
|  | ND | ND | Digestive system | Root, Leaves, Stem. | Jacobo, et al., 2016 |
|  | Cordón de Jesús | Hgo | Cultural-bound syndromes (“espanto”) | Flowers, Decoction, Beverage | Aguilar et al., 1996 |
|  | Cordón de San Francisco | Mex | Pregnancy, childbirth, puerperium | Aerial parts, Decoction, Beverage | Aguilar et al., 1996 |
|  | Lana | Mex | Digestive system, Genitourinary system | ND | Aguilar et al., 1996 |
|  | Moradoxochitl | Mor | Pregnancy, childbirth, puerperium | Aerial parts, Decoction, Baths | Monroy and Castillo, 2000 |
| ***Salvia longispicata* Martens et Gal.** | ND | ND | Symptoms and signs | ND | Heinrich, 1992 |
| ***S. melissodora* Lag.** | Orégano, tkulh origan (Tepehuano). | Dgo | Symptoms and signs | Aerial parts, Poultice, Topical | González et al., 2004 |
|  | Salvia | Mich | Digestive system | ND | Bello and Salgado, 2007 |
|  | ND | Oax | Culture-bound syndromes, Digestive system, Genitourinary system, Nervous system | ND | Cruz-Pérez et al., 2021 |
| ***S. mexicana* L.** | Chía gorda, tacote, tapachichi | Dgo | Digestive system | Roots, Decoction, Beverage | González et al., 2004 |
|  | Tlanchichinole | Hgo | Digestive system, Genitourinary system | Decoction, Beverage and topical | Andrade, 2009 |
|  | Chía, chía corriente, chía de monte | Mich | Digestive system, Respiratory system | Seeds in water, Beverage.  Root, Decoction, Topical (fomentation) | Toledo, 2014 |
|  | Chía | Mich | Digestive system | ND | Bello and Salgado, 2007 |
|  | Achía, chía, achicha morada | Mex | Visual system, Injury, Symptoms and signs | Aerial part, Decoction, Eye washes  Crushed, Topical | Jiménez, 1994 |
|  | Chía gorda, tet-she (cora) | Nay | Digestive system | Roots, Decoction, Drink | Rodríguez, 1992 |
|  | ND | Chis | Pregnancy, childbirth, puerperium | ND | Breedlove and Laughlin, 1993 |
|  | Chía, charahuesca, ichukuta, azul-sipari | Mich | Digestive system, Genitourinary system, Respiratory system | Leaves, Seeds and Flowers, Decoction, Beverage | Bello et al., 2015 |
|  | ND | Oax | Endocrine diseases | ND | Cruz-Pérez et al., 2021 |
|  | Mirto morado | Mex | Culture-bound syndromes (“espanto”) | Decoction | Lozano, 1996 |
|  | Salvia del monte | Son | Digestive system | ND | López e Hinojosa, 1988 |
|  | Marrubio | Tlax | Digestive system | Decoction | Aguilar et al., 1996 |
| ***S. microphylla* Kunth** | Mirto, mirto chico, pabellón mexicano, salvia del monte, toronjil, bandera mexicana. | Chih, SLP, Pue, Mex | ND | ND | Standley, 1923 |
|  | Mirto | Mex | Nervous system, sleep-wake disorders | Aerial parts, Decoction, Beverage and Baths  Aerial parts, Crude, Under pillow | Heras and Ariza, 2007 |
|  | Mirto, mirto de huerto | Dgo | Ears, Nervous system, Cultural-bound syndromes (“limpias”, “mal de ojo”) | Leaves, Roasted | González et al., 2004 |
|  | Mirto rosa | Hgo | Nervous system, Sleep-wake disorders, Cultural-bound syndromes (“susto”) | Aerial parts, Decoction, Bath  Aerial parts, Crude, Under pillow | Villavicencio and Pérez, 2002 |
|  | Mirto | Ags | Digestive system, Symptoms and signs, Nervous system, sleep-wake disorders | Aerial parts, Decoction, Beverage Aerial parts, Rubbed on the head | García, 2014 |
|  | Mirto | Mich | Nervous system, Sleep-wake disorders | Aerial parts, Decoction, Beverage | Arriaga et al., 2007 |
|  | Mirto, mirto chico, ix tasalak (tepehua). | Pue | Digestive system | Aerial parts, Decoction, Rubbed with oil on the stomach | Martínez et al., 1995 |
|  | Mirto | Mich | Digestive system, Respiratory system, Pregnancy, childbirth, puerperium, Visual system  Nervous system, Sleep-wake disorders, Cultural-bound syndromes (“susto”) | Aerial parts, Infusion, Beverage or Baths  Flowers, Scrubbed, the juice on the eyelid  Aerial parts, Decoction, Beverage | Toledo, 2014 |
|  | Mirto, mirto rojo | Hgo | Nervous system, Sleep-wake disorders, Musculoskeletal system, Cultural-bound syndromes (“empacho”) | ND | Molina et al., 2012 |
|  | ND | Mich | Digestive system | ND | Hurtado and Rodríguez, 2006 |
|  | Mirto | Ver | Pregnancy, childbirth, puerperium, Digestive system, Infectious diseases, Symptoms and signs, Cultural-bound syndromes (“aljorra”). | Aerial parts, Decoction, Bath | Cabrera, 2010 |
|  | Mirto | Mex | Visual system,  Respiratory system,  Digestive system. | Aerial part. Decoction in washes or Beverage  Aerial part. Decoction with alcohol  Beverage | Jiménez, 1994 |
|  | Chupamirto | Mex | Digestive system, Ears | Leaves, Decoction, Beverage or Topical | Cazarez, 1994 |
|  | Mirto | Pue | Digestive system | ND | Gómez, 2013 |
|  | Mirto, diente de acamaya, ix tasalak (tepehua). | Pue | Digestive system, infectious diseases, Visual system | Aerial parts, Decoction, Beverage  Flowers, Scrubbed, the juice on the eyelids | López, 1988 |
|  | Rama negra | SLP | Pregnancy, childbirth, puerperium | Aerial parts, Decoction, Beverage | Rodríguez, 1994 |
|  | Mirto | Hgo | Nervous system, Sleep-wake disorders, Cultural-bound syndromes (“espanto”) | Aerial parts, Decoction, Beverage, or Baths | Martínez, 2007 |
|  | Mirto rojo, mirto chiquito, mistro | Mex | Cultural-bound syndromes (“espanto, “dolor de aire”), Digestive system | Aerial parts, Decoction drink and Baths  Aerial parts, Macerated in rubbing alcohol, Rubbing | Lozano, 1996 |
|  | Mirto | Mich | Digestive system, Nervous system, Sleep-wake disorders | Leaves & flowers, Decoction | Aburto, 2013 |
|  | Mirto | Hgo | Respiratory system | Aerial parts, Decoction.  Inhalation of steam | Flores, 1998 |
|  | Mirto | Hgo | Cultural-bound syndromes (“espanto”) | Aerial parts, Scrubbed in water, Baths | López, 2009 |
|  | Mirto | Hgo | Digestive system, Cultural-bound syndromes (“susto”), Circulatory system | Aerial parts, Decoction, Beverage | Villanueva et al., 2020 |
|  | ND | Chis | Pregnancy, childbirth, puerperium | ND | Breedlove and Laughlin, 1993 |
|  | Mirto | Ags | Digestive system, Symptoms and signs | Leaves, Decoction, Beverage  Leaves, Crude, Rubbed on the head | Barba et al., 2003 |
|  | Mirto | CDMX | Nervous system | Aerial parts | Guzmán et al., 2014 |
|  | Mirto | NL | Digestive system, Infectious diseases, Cultural-bound syndromes (“susto”) | Aerial parts, Decoction, Beverage, Bath  Aerial parts/ branches, Sweeps | Estrada et al., 2014 |
|  | ND | Oax | Digestive system, Genitourinary system, Nervous system | ND | Cruz-Pérez et al., 2021 |
|  | Mirto, blackcurrant sage | ND | ND | ND | Calzada and Bautista, 2020 |
|  | Litropueti | Pue | Symptoms and signs | Aerial parts, Crude, Topical, Rubbed | Aguilar et al., 1996 |
|  | Mirto | Tam | Cultural-bound syndromes (“empacho”) | Decoction | Aguilar et al., 1996 |
|  | Mirto | Jal | Pregnancy, childbirth, puerperium | Aerial parts, Decoction, Beverage | Aguilar et al., 1996 |
|  | Mirto | NL | Digestive system, Cultural-bound syndromes (“dolor de aire”) | Aerial parts, Decoction, Beverage | Aguilar et al., 1996 |
|  | Mirto | Mich | Digestive system | ND | Aguilar et al., 1996 |
|  | Mirto | Hgo | Nervous system | Flowers, Decoction, Beverage | Aguilar et al., 1996 |
|  | Mirto | Mex | Cultural-bound syndromes (“susto”, “friegas”) | ND | Aguilar et al., 1996 |
|  | Mirto | Mex | Cultural-bound syndromes (“coraje”) | Decoction | Aguilar et al., 1996 |
|  | Mirto | Mex | Nervous system | Aerial parts, Crude | Aguilar et al., 1996 |
|  | Mirto | Gto | Digestive system | ND | Aguilar et al., 1996 |
|  | Mirto | Pue | Cultural-bound syndromes (“espanto”) | Leaves, Dried, as suppository or ‘pelotillas’ | Aguilar et al., 1996 |
|  | Mishto (Tzotzil) | Chis | Pregnancy, childbirth, puerperium | Aerial parts, Beverage | Aguilar et al., 1996 |
|  | Tzil bimol (Tzotzil) | Chis | Digestive system, Symptoms and signs | Aerial parts, Decoction, Beverage | Aguilar et al., 1996 |
|  | Mirtus, mirto | Pue | Cultural-bound syndromes (“susto”, “quemado”), Skin,  Genitourinary system | Decoction, Baths  Aerial parts, Sweeps or “limpias” | Aguilar et al., 1996 |
|  | Mirto, salve real larga | Mor | Digestive system, Cultural-bound syndromes (“susto de niño”), Symptoms and signs | Poultice | Aguilar et al., 1996 |
|  | Mirto | SLP, Mex | Symptoms and signs | Aerial parts, Decoction | Standley, 1923 |
|  | ND | Mich | Digestive system | ND | Hurtado and Rodríguez, 2006 |
| ***S. misella* Kunth** | Verbena, lengua de toro, hierba del cáncer. | Dgo | Injury | ND | González et al., 2004 |
|  | Verbena, cocuc-sha (cora | Nay | Injury | Decoction or Crushed, Poultice | Rodríguez, 1992 |
|  | ND | Chis | Symptoms and signs | ND | Breedlove and Laughlin, 1993 |
|  | ND | Oax | Musculoskeletal system, Skin | ND | Cruz-Pérez et al., 2021 |
|  | Mukuy (maya), xiw (maya), hierba santa | Q. Roo | Skin | ND | Pulido and Serralta, 1993 |
|  | Hierba del cáncer | Mor | Digestive system, Infectious diseases, Symptoms and signs, Skin, Injury | Aerial parts, Decoction, Washes  Leaves, Crushed, Poultice | Monroy and Castillo, 2000 |
| ***S. oaxacana* Fernald** | ND | Oax | Endocrine diseases | ND | Cruz-Pérez et al., 2021 |
| ***S. patens* Cav.** | Mirto azul | Hgo | Musculoskeletal system | ND | Molina et al., 2012 |
|  | Gallitos | Hgo | Injury | Leaves and flowers, Crushed, Massage | Martínez, 2007 |
| ***S. polystachia* Cav.** | Hierba chica | Dgo | Injury (wounds) | Decoction, Wash wounds | González et al., 2004 |
|  | Chía, parhi, salvia | Mich | Digestive system, Injury (wounds), Pregnancy, childbirth, puerperium | Decoction, Wash wounds  Decoction, Beverage | Toledo, 2014 |
|  | Chía | Mich | Digestive system | Decoction, Drank hot | Bello and Salgado, 2007 |
|  | Chía | Mor | Pregnancy, childbirth, puerperium, Symptoms and signs | Aerial parts | Maldonado, 1997 |
|  | Chía | Mich, Gro | Cultural-bound syndromes (“aire”) | Leaves, Crushed in rubbing alcohol, Rub tincture | Soto, 1987 |
|  | Chía | Gto | Symptoms and signs | Leaves, Decoction, Beverage | Estrada, 1984 |
|  | Chía, achian | Mex | Symptoms and signs | Seeds, in water, Beverage | Cazares, 1994 |
|  | Chía | Mich | Digestive system, Symptoms and signs | Aerial parts, Decoction, Beverage | Cornejo e Ibarra, 2008 |
|  | Ulcema | Ver | Digestive system, Skin, (makes hair grow) | Aerial parts | Navarro and Avendaño, 2002 |
|  | Lucemilla, azuema, chía | Mich | Digestive system, Genitourinary system | Aerial parts, Decoction, Beverage | Bello et al., 2015 |
|  | Chía | CDMX | Nervous system | Aerial parts | Guzmán et al., 2014 |
|  | ND | Oax | Digestive system, Skin | ND | Cruz-Pérez et al., 2021 |
|  | Chía, romerillo | ND | Digestive system | Leaves | Calzada and Bautista, 2020 |
|  | Chía, osturaqui, tlacuchichea | Mor | Symptoms and signs, Cultural-bound syndromes (“sacar el diablo”) | Leaves, Crushed, Smelling the crushed leaves  Leaves, Burned in the house | Monroy and Castillo, 2000 |
|  | Alchichia | Mex | Digestive system | Decoction | Aguilar et al., 1996 |
| **S. *protracta* Benth.** | Chilla, jehuite bendito | Pue | Digestive system | Decoction | Martínez et al., 1995 |
|  | tlachapahuastle (nahua) | Pue | Cultural-bound syndromes (“mal de ojo”), Injury, Pregnancy, childbirth, puerperium | Aerial parts, Decoction, Washes  Aerial parts, Decoction, Bath | Castro, 1988 |
|  | Chilla, jegüite blandito, xitexe (otomí), yemanquixiuitl (nahua). | Pue | Digestive system, Injury (wounds) | Leaves, Decoction, Beverage | Castro, 1988 |
| ***S. purpurea* Cav.** | Hierba de muerto, flor de muerto, ginutno (cuicateco), jinubitinoo (cuicateco). | Oax. | Neoplasms, Symptoms and signs, Cultural-bound syndromes (“mal aire de muerto”), Musculoskeletal system | Decoction, Bath | Mercado, 2013 |
|  | Mirto de campo | Ver | Pregnancy, childbirth, puerperium | Decoction, Bath | Cabrera, 2010 |
|  | La rla (zapoteco | Oax | ND | Leaves | Luna and Rendón, 2008 |
|  | Flor de morada, ramoncillo sirani tsitsiki | Mich | Digestive system | Aerial parts, Decoction, Beverage | Bello et al., 2015 |
|  | ND | Oax | Culture-bound syndromes | ND | Cruz and Pérez et al., 2021 |
| ***S. prunelloides* Kunth** | Orejas de venado, suimalh nanakl (tepehuano) | Dgo | Genitourinary system | ND | González et al., 2004 |
| ***S. reflexa* Hornem**. | Hierba del pajarito | Dgo | Digestive system, Infectious diseases, Musculoskeletal system | Decoction | González et al., 2004 |
|  | Chía | NL | Digestive system | Decoction | Aguilar et al., 1996 |
| ***S. regla* Cav.** | Salvia | NL | Symptoms and signs | Leaves, Decoction, Beverage | Estrada et al., 2007 |
| ***S. reptans* Jacq.** | Hierba de pozuña, hierba del pollo, fiño tr’axo, kampfiño (mazahua) | Mex | Symptoms and signs, Genitourinary system, Digestive system | Aerial parts, Decoction, Washes  Aerial parts, Decoction, Beverage | Lozano, 1996 |
|  | Romerillo | Hgo | Digestive system | Aerial parts, Decoction, Beverage | Aguilar et al., 1996 |
|  | Hierba de la golondrina | Méx | Symptoms and signs,  Digestive system | Aerial parts, Decoction,  Drink | Aguilar et al., 1996 |
| ***S. rubiginosa* Benth.** | ND | Chis | ND | Steam cures | Breedlove and Laughlin, 1993 |
| ***S. schaffneri* Fernald** | Mirto grande | SLP | ND | ND | Standley, 1923 |
| ***S. semiatrata*** | Mirto morado | Oax | Musculoskeletal system, Injury, Symptoms and signs, Ears, Digestive system, Nervous system, Pregnancy, childbirth, puerperium | Aerial parts, Decoction, Topical (rubbing the affected part) | Nambo, 2015 |
| ***S. sessei* Benth.** | ND | Oax | ND | ND | Cruz-Pérez et al., 2021 |
| ***S. shannonii* Donn. Sm.** | Monte amargo | ND | Digestive system, Infectious diseases | Leaves | Calzada and Bautista, 2020. |
| ***S. splendens* Sellow ex Wied-Nees (introduced species)** | ND | Oax | ND | ND | Cruz-Pérez et al., 2021 |
| ***S. serotina* L.** | Juku´wel (Chol) | Chis | Musculoskeletal system | Decoction, Topical (rubbing the affected part) | Cahuich et al., 2014 |
|  | Pasmar xiw (maya) | Yuc | Digestive system | Leaves, Decoction or Crushed in water, Baths | Cahuich et al., 2014 |
|  | Hoja de cólico, flor de canica | Tab | Digestive system | Leaves, Decoction, Beverage | Villarreal et al., 2014 |
|  | Chía | Hgo | Symptoms and signs (“Chincual”) | Aerial parts, Decoction, Topical | López, 2009 |
| ***S. stolonifera* Benth.** | ND | Oax | ND | ND | Cruz-Pérez et al., 2021 |
| ***S. tiliifolia* Vahl** | Chupona | Hgo | Infectious diseases, Symptoms and signs | ND | Molina et al. 2012 |
|  | ND | Mich | Digestive system | ND | Hurtado and Rodríguez, 2006 |
|  | ND | Chis | Injury | ND | Breedlove and Laughlin, 1993 |
|  | Hierba del cólera | Pue | Endocrine diseases | Leaves, flower | Martínez et al., 2016 |
|  | ND | Oax | ND | ND | Cruz-Pérez et al., 2021 |
|  | Chinatuwan | Pue | Injury (wounds) | Leaves, Decoction, Wash injured part | Guerrero, 2020 |
|  | Salvia | Son | Digestive system | ND | López and Hinojosa, 1988 |
| ***S. thymoides* Benth.** | Gobernadora | Pue | Digestive system | ND | Gómez, 2013 |
|  | Gobernadora | Pue | Digestive system | ND | Sentíes, 1984 |
|  | ND | Oax | ND | ND | Cruz-Pérez et al., 2021 |
| ***S. xalapensis* Benth.** | Shcoyo ni (Mazateco) | Pue | Infectious or parasitic diseases | Decoction | Aguilar et al., 1994 |
| ***S. urica* Epling** | ND | Oax | ND | ND | Cruz-Pérez et al., 2021 |
| *Abbreviations of States (Mexico): Ags= Aguascalientes, Chih= Chihuahua, CDMX= Mexico City, Chis=Chiapas, Dgo= Durango, Gro= Guerrero, Gto= Guanajuato, Hgo= Hidalgo, Jal=Jalisco, Mex= State of México, Mich= Michoacán, Mor= Morelos, Nay= Nayarit, NL= Nuevo León, Oax= Oaxaca, Pue= Puebla, Q. Roo= Quintana Roo, SLP= San Luis Potosí, Son= Sonora, Tab= Tabasco, Tam= Tamaulipas, Tlax= Tlaxcala, Ver= Veracruz, Yuc= Yucatán, Zac= Zacatecas. | | | | | |

**Literature cited**

Aburto, M. (2013). *Plantas medicinales silvestres y de traspatio de Yoricostio, municipio de Tacámbaro, Michoacán, México*. Tesis profesional. Facultad de Biología, UMSNH. Morelia, Michoacán.

Aguilar, A., Camacho, J.R., Chino, S., Jacquez, P. and López, ME. (1996). *Herbario medicinal del Instituto Mexicano del Seguro Social*. Información etnobotánica. IMSS. México, D.F.

Alcorn, J. (1984). *Huastec maya ethnobotany.* University of Texas Press Austin, TX.

Alonso, A., Domínguez F. and Zapata, J.R. (2015). Plants used in the traditional medicine of Mesoamerica (Mexico and Central America) and the Caribbean for the treatment of obesity. *J. Ethnopharmacol.* 175, 335-345. doi: 10.1016/j.jep.2015.09.029.

Andrade, A. and Heinrich, M. (2005). Mexican plants with hypoglycemic effects used in the treatment of diabetes. *J. Ethnopharmacol.* 99, 325-348. DOI: 10.1016/j.jep.2005.04.019.

Andrade, A. (2009). Ethnobotanical study of the medicinal Plants from Tlanchinol, Hidalgo, México. *J. Ethnopharmacol.* 122: 163-171. DOI: 10.1016/j.jep.2008.12.008.

Arellano, B. (2017). *Etnobotánica medicinal de la cultura me’phaa en La Ciénega, municipio de Malinaltepec, Guerrero*. Tesis Maestría, UAG. Igual, Guerrero.

Argueta, A., Cano L., Rodarte, M. and Gallardo, C. (1994). *Atlas de las plantas de la medicina tradicional mexicana*. Instituto Nacional Indigenista. 1786 pp.

Arriaga, S. (2007). *Estudio y colección viva de plantas medicinales nativas y formación de un banco de germoplasma del estado de Michoacán*. Informe final.

Barba, M.D., Croce, M. and de la Cerda, M. (2003). *Plantas útiles de la región semiárida de Aguascalientes.* UAA. Aguascalientes.

Bello, M. and Salgado, R. (2007). Plantas medicinales de la comunidad indígena Nuevo San Juan Parangaricutiro, Michoacán, México. *Biológicas* 9, 126-138.

Bello, M.F., Hernández, S., Chávez, B. and Salgado, R. (2015). Plantas útiles de la comunidad indígena Nuevo San Juan Parangaricutiro, Michoacán, México. *Polibotánica* (39), 175-215. DOI: 10.18387/polibotanica.39.10.

Bisio, A., Schito, A. M., Ebrahimi, S. N., Hamburger, M., Mele, G., Piatti, G., et al. (2015). Antibacterial compounds from *Salvia adenophora* Fernald (Lamiaceae). *Phytochemistry* 110, 120–132. doi:10.1016/j.phytochem.2014.10.033.

Breedlove, D. and Laughlin, R. (1993). *The flowering of man. A tzotzil botany of Zinacantan*. Vol. II. Smithsonian Contributions to Anthropology No. 35. Smithsonian Institution Press. Washington D. C.

Cabrera, R. (2010). *Plantas medicinales del municipio de Tlalnelhuayocan, Veracruz*. Tesis profesional. Facultad de Biología UC. Xalapa, Veracruz.

Cahuich, D., Cano, L., Hernández, A., Hirose, J., Huicochea, L., Mondragón, R., Sánchez, O.M., Serralta, L., Torrescano, N. and Vera, G. (2014). *Herbolaria curativa y sanadora: La experiencia terapéutica de hombres y mujeres del sur-sureste mexicano*. Secretaría de cultura del estado de Campeche. San Francisco de Campeche.

Calzada, F. and Bautista, E. (2020). Plants used for the treatment of diarrhoea from Mexican flora with amoebicidal and giardicidal activity, and their phytochemical constituents. *J. Ethnopharmacol.* 253, 112676. doi: 10.1016/j.jep.2020.112676.

Campos-Xolalpa, N., Alonso-Castro, Á. J., Ortíz-Sánchez, E., Zapata-Morales, J. R., González-Chávez, M. M., and Pérez, S. (2021). Anti-inflammatory and antitumor activities of the chloroform extract and anti-inflammatory effect of the three diterpenes isolated from *Salvia ballotiflora* Benth. *BMC Complement. Med. Ther*. 21, 1-11. doi:10.1186/s12906-020-03179-w.

Castro, A. (1988). *Estudio comparativo del conocimiento sobre plantas medicinales utilizadas por dos grupos étnicos del municipio de Pahuatlán, Puebla.* Tesis profesional. ENEP Iztacala UNAM. Tlalnepantla.

Castro, C., Villa, N., Ramírez, S.A. and Mosso C. (2014). Medicinal use of antidiabetic plants in Oaxaca ethnobotanical tradition. *Revista Cubana de Plantas Medicinales* 19 (1), 101-120.

Cazares, A. (1994). *Catálogo de Plantas medicinales del estado de México.* Tesis profesional. ENEP Iztacala, UNAM. Tlalnepantla.

Cervantes, L. (1979). *Plantas medicinales del distrito de Ocotlán en la región de los Valles Centrales de Oaxaca*. Tesis profesional. Facultad de Ciencias, UNAM. México, D.F.

Chino, S. and Jacquez, P. (1986). *Contribución al conocimiento de la flora medicinal de Quimixtlán, Puebla*. Tesis profesional. ENEP Iztacala, UNAM. Tlalnepantla.

Cornejo, G. and Ibarra, G. 2008. *Flora ilustrada de la Reserva de la mariposa monarca*. UNAM, CONABIO. México, D.F.

Cruz-Pérez, A., Barrera, J., Bernal, L., Bravo D. and Rendón, B. (2021). Actualized inventory of medicinal plants used in traditional medicine in Oaxaca, Mexico. *J. Ethnobiol. Ethnomedicine* 17 (7), 1-15. doi.org/10.1186/s13002-020-00431-y.

del Amo, S. (1979). *Plantas medicinales del estado de Veracruz*. INIREB. Xalapa, Veracruz.

De La Cruz-Jiménez, L., Guzmán-Lucio, M., and Viveros-Valdez, E. (2014). Traditional medicinal plants used for the treatment of gastrointestinal diseases in Chiapas, México. *World Appl. Sci. J.* 31, 508–515. doi:10.5829/idosi.wasj.2014.31.04.8381.

Diaz, J.L. (2013) *Salvia divinorum*: A psychopharmacological Riddle and a Mind-Body prospect. *Curr. Drug Abuse Rev.* 6, 43-53. doi: 10.2174/18744737112059990004.

Domínguez, X.A., González, H.F., Aragón, R., Gutiérrez, M., Marroquín, J.S. and Watson, W. (1976). Mexican medicinal plants XXIX three new diterpene quinones from *Salvia ballotaeflora*. *Planta Med.* 30(7), 237-241. doi:10.1055/s-0028-1097724.

Domínguez G. and Castro, A. (2002). Usos medicinales de la familia Labiatae en Chiapas, México. *Etnobiología* (2), 19-31.

Domínguez, C., Cruz, E. and González C. (2015). Plantas de uso medicinal de la Reserva Ecológica Sierra de Otontepec, municipio de Chontla, Veracruz, México. *Ciencia UAT* 9(2), 41-52.

Espinosa, A. (1985). *Plantas medicinales de la Huasteca Hidalguense*. Tesis profesional. Facultad de Ciencias, UNAM. México, D.F.

Esquivel, R., Pérez-Cálix, E., Ochoa, A. and García, M. (2018). Ethnomedicinal plants used for the treatment of dermatological affections on the Purépecha Plateau, Michoacán, México. *Acta Bot. Mex.* 125, 95-132. doi.org/10.21829/abm125.2018.1339.

Estrada, E. (1984). *Las plantas medicinales y los sistemas tradicionales de curación del municipio de Dr. Mora, Guanajuato*. Tesis profesional. ENEP Iztacala, UNAM. Tlalnepantla.

Estrada, E., Garza, M., Villareal, J.A., Salinas, M.M., Soto, B.E., González, H., González, D.U., Cantú, I., Carrillo, A. and Cantú C. (2014) Ethnobotany in Rayones, Nuevo León, México. *J. Ethnobiology and Ethnomedicine* 10 (62), 1-13. doi.org/10.1186/1746-4269-10-62.

Estrada E., Villarreal, J.A., Cantú, C., Cabral, I., Scott L. and Yen C. (2007). Ethnobotany in the Cumbres de Monterrey National Park, Nuevo León, México. *J. Ethnobiol. Ethnomed.* 3(8), 1-8. doi: 10.1186/1746-4269-3-8.

Flores, J. (1998). *Estudio etnobotánico de las plantas medicinales de Tolcayuca, Hidalgo*. Tesis profesional. ENEP Iztacala, UNAM. Tlalnepantla

García, G. (2014). *Plantas medicinales de Aguascalientes*. UAA. Aguascalientes. 498 pp.

Gómez, A. (2013). *Conocimiento tradicional sobre Plantas medicinales en huertos familiares de la mixteca poblana, México*. Tesis doctoral. CP campus Puebla. Puebla.

González, M., López, L., González, S. and Tena, J. (2004). *Plantas medicinales del estado de Durango y zonas aledañas*. IPN. México, D.F.

González, M. (2010). *Plantas medicinales de Nuevo León*. En: De la lechuguilla a las biopelículas vegetales. Las Plantas útiles de Nuevo León. UANL. Monterrey, México. pp: 109-143.

Guerrero, V. (2020). *Estudio etnobotánico de las plantas medicinales y conocimiento tradicional de la localidad totonaca de Dimas López en Olintla, Puebla, México*. Tesis profesional. Facultad de Ciencias, UNAM. Ciudad de México.

Guzmán, L., Reyes, R. and Bonilla, H. (2014). Medicinal Plants for the treatment of “nervios”, anxiety and depression in Mexican Traditional Medicine. *Rev. Brasileira Farmacog.* 24, 591-608. doi.org/10.1016/j.bjp.2014.10.007.

Heinrich, M. (1992). *Economic Botany of American Labiatae*. In R.M. Harley and T. Reynolds (Ed.). Advances in Labiatace Science, pp. 475-488.

Heras, A. and Ariza, MR. (2007). *Olor a hierba. Biodiversidad medicinal del Volcán Popocatépetl.* Ediciones Papiro Omega. Morelia, Mich.

Hurtado, N. and Rodríguez C. (2006). Estudio cualitativo y cuantitativo de la flora medicinal del municipio de Copándaro de Galeana, Michoacán, México. *Polibotánica* 22, 21-50.

Jacobo, N., Jacobo, F., Zentella A., Andrade, A., Heinrich, M. and Pérez, C. (2016). Medicinal Plants used in Mexican traditional medicine for the treatment of colorectal cancer. *J. Ethnopharmacol.* 179, 391-402. doi: 10.1016/j.jep.2015.12.042.

Jenks, A. and Seung-Chul, K. (2013). Medicinal plant complexes of *Salvia* subgenus *Calosphace* an ethnobotanical study of new world sages. *J. Ethnopharmacol.* 146 (1), 214-224. doi: 10.1016/j.jep.2012.12.035.

Jiménez, J. (1994). *Plantas medicinales de San Juan Tepecoculco, municipio de Atlautla de Victoria, estado de México*. Tesis profesional. FES-Zaragoza UNAM. México, D.F:

Juárez, C., Carranza C., Alonso A., González V., Bravo E., Chamarro F. and Solano E. (2013). Ethnobotany of medicinal plantas used in Xalpatlahuac, Guerrero, México. *J. Ethnopharmacol. 148(2), 521-527.* doi: 10.1016/j.jep.2013.04.048.

Ku, G. (2018). *Etnobotánica médica de Uayma, Yucatán.* Tesis profesional. Facultad de Ciencias, UNAM. Ciudad de México.

López, M.E. (1988). *Contribución etnobotánica en plantas medicinales utilizadas por dos grupos étnicos de Mecapalapa, municipio de Pantepec, Puebla.* Tesis profesional. ENEP Iztacala, UNAM. Tlalnepantla.

López, A. (1994). *Contribución al conocimiento de las Plantas medicinales de los tianguis de la zona metropolitana de Guadalajara, Jalisco*. Tesis profesional. Facultad de Ciencias Biológicas, U de G. Guadalajara, Jalisco.

López, ME. (2009). *Etnobotánica médica de los tepehuas de Hidalgo*. Tesis Maestría. Facultad de Ciencias, UNAM. México, D.F.

López, R. and Hinojosa A. (1988). *Catálogo de plantas medicinales sonorenses*. Universidad de Sonora. Hermosillo, Sonora.

Lozano G. (1996). *Plantas medicinales utilizadas por los mazahuas del municipio de San Felipe del Progreso, estado de México*. Tesis profesional. Facultad de Ciencias, UNAM. México, D.F.

Luna, A. and Rendón, B. (2008). Recursos vegetales útiles en diez comunidades de la Sierra Madre del Sur, Oaxaca, México. *Polibotánica* 26, 193-242.

Macouzet, M., Estrada, E., Jiménez, J., Villareal, J. and Herrera, M. (2013). *Plantas medicinales de Miquihuana, Tamaulipas*. UANL. Monterrey, N.L.

Maldonado, B. (1997). *Aprovechamiento de los recursos florísticos de la Sierra de Huautla, Morelos, México*. Tesis de Maestría. Facultad de Ciencias, UNAM. México, D.F.

Martínez, M. (1975). *Flora medicinal del estado de México*. Gobierno del estado de México. Toluca, México.

Martínez, M.A., Evangelista, V., Mendoza, M., Morales, G., Toledo, G: and Wong, A. (1995). *Catálogo de Plantas útiles de la Sierra Norte de Puebla, México.* Cuadernos 28, Instituto de Biología, Universidad Nacional Autónoma de México. México, D. F.

Martínez, E. (2007). *Plantas medicinales de la comunidad de Plomosas, municipio de Actopan, Hidalgo*. Tesis profesional. UAEH. Mineral de la Reforma, Hidalgo.

Martínez, J. (2016). *Flora médica de Santiago Pinotepa Nacional, Oaxaca e inventario de Plantas de Santa María Huatulco*. Tesis profesional. FES-I, UNAM. Tlalnepantla.

Martínez, D., Valdez, G., Basurto, F., Andrés, A., Rodríguez T. and Figueroa A. (2016). Plantas medicinales de los mercados de Izúcar de Matamoros y Acatlán de Osorio, Puebla. *Polibotánica* 41, 153-178.

Mendoza, B. (1983). *Estudio etnobotánico de plantas medicinales en el ejido Santa Ana, Teoloyucan, estado de México*. Tesis profesional. Facultad de Ciencias, UNAM. México, D. F.

Mercado, A. (2013). *Estudio de Plantas medicinales usadas por cuicatecos en la localidad de Santos Reyes Pápalo, Cuicatlán, Oaxaca.* Tesis profesional. Facultad de Ciencias, UNAM.

Molina, J., Galván, R., Patiño, A. and Fernández R. (2012). Plantas medicinales y listado florístico preliminar del municipio de Huasca de Ocampo, Hidalgo, México. *Polibotánica* 34, 259-291.

Monroy, R. (2016). *Conocimiento en la localidad de origen otomí Jiquipilco el Viejo, Temoaya, México*. Tesis de Maestría. UAEMex. El Cerrillo, Toluca, México.

Monroy, C. and Castillo, P. (2000). *Plantas medicinales utilizadas en el estado de Morelos.* CIB, UAEM. Cuernavaca, Morelos.

Mambo, A. (2015). *Etnobotánica de Santiago Huauclilla, Oaxaca y evaluación farmacológica de* *Zinnia peruviana*. Tesis profesional. Facultad de Ciencias, UNAM. México, D. F.

Naranjo, M. (2012). *Etnobotánica de las Plantas vasculares de San Andrés Chicahuaxtla, Putla, Oaxaca*. Tesis profesional. FAS Zaragoza, UNAM. México, D. F.

Navarro L. and Avendaño, S. (2002). Flora útil del municipio de Astacinga, Veracruz, México. *Polibotánica* 14, 67-84.

Pulido, MT. and Serralta, L. (1993). *Lista anotada de las plantas medicinales de uso actual en el estado de Quintana Roo, México*. Centro de Investigaciones de Quintana Roo. Chetumal, Q. Roo.

Romero, T. (2018). *Estudio etnobotánico de las plantas medicinales de San José Vista Hermosa. Oaxaca, y evaluación farmacológica de* *Plectranthus scutellarioides* (L.) R.Br. Tesis profesional. Universidad de la Sierra Juárez. Ixtlán de Juárez, Oaxaca.

Rodríguez, H. (1992). *Las plantas medicinales silvestres en tres comunidades indígenas de Nayarit-coras y tepehuanos.* Tesis profesional. Facultad de Ciencias, UNAM. México, D.F.

Rodríguez, T. (1994). *Prácticas terapéuticas y medicinales en los aspectos ginecológicos de las nahuas de San Luis Potosí*. Tesis profesional. ENEP Iztacala, UNAM. Tlalnepantla

Senties, A. (1984). *Plantas medicinales y sistemas tradicionales de curación del Valle de Tehuacán, Puebla*. Tesis profesional. Facultad de Ciencias, UNAM. México, D.F.

Serrano-Vega, R., Pérez-González, C., Alonso-Castro, Á., Zapata-Morales, J., and Pérez-Gutiérrez, S. (2020). Anti-inflammatory and antinociceptive activities of *Salvia keerlii*. *Pharmacogn. Mag.* 16 (67), 27-33. doi:10.4103/pm.pm_223_19.

Solano, L. (2008). *Etnobotánica de las plantas vasculares del municipio de Putla, Oaxaca, México*. Tesis profesional. FES Zaragoza, UNAM. México, D.F.

Solano, C. and Blancas, J. (2018). Etnobotánica de wirikuta: uso de recursos vegetales silvestres en el desierto de San Luís Potosí, México. *Etnobiología* 16 (3), 54-77.

Soto J. (1987). *Plantas medicinales y su uso tradicional en la cuenca del río Balsas, estados de Michoacán y Guerrero, México*. Tesis profesional. Facultad de Ciencias, UNAM. México, D.F.

Standley, P. (1923). Trees and shrubs of Mexico. Contributions from the US National Herbarium. *Salvia*. Pp 1256-1269.

Toledo, M. (2014). *Plantas nativas medicinales del municipio de Morelia, Michoacán*. Tesis profesional. Universidad de Guadalajara. Zapopan, Jalisco.

Valdés III, L. J., Díaz, J., and Paul, A. G. (1983). Ethnopharmacology of ska maria pastora (*Salvia divinorum*, Epling and Játiva-M.). *J. Ethnopharmacol.* 7(3), 287-312.

Villanueva, I., Arreguín, M., Quiroz, D. and Fernández, R. (2020). Plantas medicinales que se comercializan en el mercado 8 de julio y uno tradicional, ambos localizados en el centro de Actopan, Hidalgo, México. *Polibotánica* 50, 209-243.

Villarreal, E., García, E., López, P., Palma, D., Lagunez, L., Ortiz, F. and Oranday, A. (2014). Plantas útiles de la medicina tradicional de Malpasito, Huimanguillo, Tabasco, México. *Polibotánica* 37, 109-134.

Villavicencio, M.A. and Pérez B. (2002). *Plantas útiles del estado de Hidalgo II*. UAEH. Pachuca, Hidalgo.

White, L., Juan, J., Chávez, C. and Gutiérrez, J. (2013). Flora medicinal en San Nicolás, municipio de Malinalco, estado de México. *Polibotánica* 35, 173-206.

Zamora, M. and Nieto, C. (1992). Medicinal plants used in some rural populations of Oaxaca, Puebla and Veracruz, Mexico. *J. Ethnopharmacol.* 35, 229-257. doi: 10.1016/0378-8741(92)90021-i.
