# Supplementary material for: A Review on the Ethnopharmacology and Phytochemistry of the Neotropical Sages (Salvia Subgenus Calosphace; Lamiaceae) Emphasizing Mexican Species
Source: Front Pharmacol. 2022 Apr 19;13:867892. doi: 10.3389/fphar.2022.867892 (PMC9061990; doi:10.3389/fphar.2022.867892)
Supplement: Supplementary file 3 [file Table2.docx]

**Supplementary Table 2.** Pharmacological activities for species of *Salvia* subgenus *Calosphace* from Mexico.

| Species  (Scientific name) | Extract or compound(s) | Part of the plant | Pharmacological activity | Positive control | Reference |
| --- | --- | --- | --- | --- | --- |
| *Salvia adenophora* Fernald | Methyl (9*S*,13*R*)-2-hydroxy-12-oxo-(10*Z*,15*Z*)-phytodienoate  Methyl (9*S*,13*S*)-2-hydroxy-12-oxo-(10*Z*,15*Z*)-phytodienoate  Methyl (9*S*,13*S*)-2-acetoxy-12-oxo-(10*Z*,15*Z*)-phytodienoate; (5*R*,8*R*,9*S*,10*R*)-15,16-diol-15,16-dihydro-hardwickiic acid; (2*S*,5*R*,8*R*,9*S*,10*R*)-2-*β*-hydroxy-16-oxo-15,16-dihydro-hardwickiic acid | Aerial parts | -Activity against *Staphylococcus epidermidis* (32 μg/mL) and *S. aureus* (64 μg/mL)  -*S. epidermidis* (21 μg/mL), *S. aureus* (64 μg/mL)  -*S. epidermidis* (75 μg/mL) | Penicillin: *S. epidermidis* and *S. aureus* (1 μg/mL) Oxacillin sodium salt: *S. epidermidis* and *S. aureus*  (6 μg/mL) | Biso et al. 2015 |
| *S. albocaerulea* Linden | 15-hydroxy-7-oxo-abieta-8,11,13-triene  Sugiol | Leaves | -*Staphylococcus aureus* (MIC 50 μg/mL),  *Bacillus subtilis* (MIC 45 μg/mL)  - *S. aureus* (MIC 50 μg/mL), *B. subtilis* (MIC 40 μg/mL) | Streptomycin sulphate:  *S. aureus* (MIC 1.56 μg/mL)*; B. subtilis* (MIC 3.12 μg/mL) | Pereda-Miranda et al. 1992 |
| *S. amarissima* Ortega | Teotihuacanin  Ethyl acetate fraction  Acetone extract  Amarissinin A  Amarissinin B  Amarissinin C  Acetone extract  Ethyl acetate fraction  5,6-dihydroxy-7,3′,4′-trimethoxyflavone  Aqueous extract  6,6″,3‴-  trihydroxy-7,3′,7″-O-trimethylloniflavone  Amarisolide A | Aerial parts  Leaves and flowers  Aerial parts | -MDA (IC_50_ 12.3 μg/mL; HeLa (IC_50_ 13.7 μg/mL); HCT-15 (IC_50_ 12.9 μg/mL); HCT-116 (IC_50_ 10.9 μg/mL)  -MDR (RF_MCF-7 /Vin+_ > 10703 at 25 μg/mL and RF_MCF-7/Vin+_ 7.2 at 5 μg/mL)  -HeLa (IC_50_ 1.50 μg/mL)  -MDR (RF^-^_MCF-7/Vin_= 54 at 25 μg/mL)  -MDR (RF^-^_MCF-7/Vin_= 36 at 25 μg/mL)  -MCF-7 (IC_50_ 18.2 μg/mL); MDA-MB-231 (IC_50_ 19.3 μg/mL); HeLa (IC_50_ 14.0 μg/mL)  -MDR (RF^-^_MCF-7/Vin_= 3.1 at 25 μg/mL)  -MDR (RF^-^_MCF-7/Vin_= 54.0 at 25 μg/mL)  -MDR (RF^-^_MCF-7/Vin_= 2.4 at 25 μg/mL)  -Activity against *E.*  *histolytica* (IC_50_ 128 μg/mL); *G.*  *lamblia* (IC_50_ 180 μg/mL)  -Activity against *E.*  *histolytica* (IC_50_ 18.9 μg/mL); *G.*  *lamblia* (IC_50_ 18.9 μg/mL)  -Antiprotozoal activity against *E.*  *histolytica* (IC_50_ 0.05 μg/mL); *G.*  *lamblia* (IC_50_ 0.13 μg/mL)  -Acute toxicity in ICR male mice  (LD_50_ > 5 g/kg).  -Oral sucrose tolerance test in male ICR mice (31.6, 100, 316 mg/kg)  -*In vitro* against mammalian α-glucosidase  (IC_50_ 39 μM)  -*In vitro* against mammalian α-glucosidase  (IC_50_ 500 μM)  -Agains a recombinant α-glucosidase with maltase-glucoamylase activity from *Ruminococcus obeum* (IC_50_ 400 μM)  -Oral sucrose tolerance test in male ICR mice (10 and 31.6 mg/kg)  -Antinociceptive effect in male CD-1 mice (1, 5 and 10 mg/kg, i.p.). Writhing test  -Antinociceptive effect in male Swiss-Webster mice (0.3 and 1 mg/kg, i.p.) Formalina ant plantar tests  -Anti-inflammatory effect in male Swiss-Webster mice (0.1 and 1 mg/kg, i.p.)  Carrageenan test | Reserpine 5 μg/mL  Reserpine RF_MCF-7/Vin+_ 4.4 at 5 μg/mL  Reserpine 5 μg/mL  Metronidazole: *E. histolytica* (IC_50_ 0.23 μg/mL); *G. lamblia* (IC_50_ 1.22 μg/mL)  Emetine *E. histolytica* (IC_50_ 2.18 μg/mL); *G. lamblia* (IC_50_ 0.83 μg/mL)  Lorke protocol.  Acarbose (5 mg/kg)  Acarbose (IC_50_ 100 μM)  Acarbose (IC_50_ 100 μM)  Acarbose (IC_50_ 1030 μM)  Acarbose (5 mg/kg)  Ketorolac (1 mg/kg, i.p.)  Tramadol (20 mg/kg, i.p.)  Indomethacin (20 mg/kg, i.p) | Bautista et al. 2015  Bautista et al. 2016  Calzada et al. 2020  Flores-Bocanegra et al. 2017  Moreno-Pérez et al. 2019  Moreno-Pérez et al. 2021 |
|  | Pedalitin  Amarisolide F  Hexane; ethyl acetate; dichloromethane/methanol; acetone; methanol; aqueous extracts  Hexane; ethyl acetate; methanol; aqueous extracts  Ethyl acetate extract  Aqueous extract  Ether extract  Ether fraction | Leaves and flowers  Aerial part | -Agains a recombinant α-glucosidase with maltase-glucoamylase activity from *Ruminococcus obeum* (IC_50_ 60 μM)  -Oral sucrose tolerance test in male ICR mice (1.0, 3.1 and 10.0 mg/kg)  -Antinociceptive effect in male CD-1 mice  (1, 5 and 10 mg/kg, i.p.). Writhing test  -Antidiabetic. Inhibition of PTP-1B activity (IC_50_ 62.0 µM/19.6 µg/mL)  -MDR (RF^-^_MCF-7/Vin_ 2.1 at 42.1 μM  (RF^+^_MCF-7/Vin_ 12 at 42.1 μM)  -Antinociceptive effect in male Swiss-Webster mice (10 mg/kg, i.p.). Formalina test  -Antinociceptive effect in male CD-1 mice  (1, 10, 100, 300 mg/kg, i.p.). Writhing test  -Acute toxicity in male CD-1 mice  (LD_50_ > 2000 mg/kg)  -Antinociceptive effect in male Swiss-Webster mice (0.1, 1, 10 mg/kg, i.p.).  Formalin test  -Anti-inflammatory effect (1 and 10 mg/kg, i.p.). Plantar test, carrageenan  -Antidiabetic: Inhibition of PTP-1B activity (IC_50_ 88.6 ± 5.4 µg/mL)  -Antidiabetic: Oral tolerance test of sucrose, starch, and glucose (50 and 300 mg/kg)  -Antidiabetic: Oral tolerance test of sucrose, starch, and glucose (50 mg/kg)  - Acute toxicity in female Balb/c mice  (LD_50_ > 3000 mg/kg) | Acarbose (IC_50_ 1030 μM)  Acarbose (5 mg/kg)  Ketorolac (1 mg/kg, i.p.)  Ursolic acid (IC_50_ 28.1 µg/mL)  Reserpine (RF_MCF/Vin –_ 5.0 and RF_MCF-7/Vin+_ 29.2)  Morphine (1 mg/kg, i.p.) and tramadol (20 mg/kg, i.p.)  Ketorolac (1 mg/kg, i.p.)  OECD 423  Tramadol (20 mg/kg, i.p.)  Tramadol; indomethacin (20 mg/kg, i.p.)  Ursolic acid (IC_50_ 28.1 µg/mL)  Acarbose  OECD 423 | Flores-Bocanegra et al. 2017  Moreno-Pérez et al. 2019  Salinas-Arellano et al. 2020  Fragoso-Serrano et al. 2019  Moreno-Pérez et al. 2021  Moreno-Pérez et al. 2019  Moreno-Pérez et al. 2021  Salinas-Arellano et al. 2020  Solares-Pascasio et al. 2021 |
| *S. anastomosans* Ramamoorthy | Cariocal | Roots | -Activity against cell line K562 (IC_50_ 32.0 μM) | Adriamycin: K562 (IC_50_ 0.3 μM) | Esquivel et al. 2005 |
| *S. ballotiflora* Benth. | Chloroform extract  Essential oil extract  β-caryophyllene  19-deoxyicetexone  Icetexone  7,20-dihydroanastomosine  19-deoxyisoicetexone  Anastomosine  7α-acetoxy-6,7-dihydroicetexone  Conacytone  6,7,11,14-tetrahydro-7-oxo-icetexone | Aerial parts | -Activity against *Spodoptera frugiperda*  (LV_50_ 1685 ppm)  -Acute toxicity in male CD-1 mice  (LD_50_ > 5000 mg/kg)  -Anti-inflammatory in male CD-1 mice (100 mg/kg). TPA-induced ear edema  -Antitumor in Balb/c mice bearing CT-26 (200, 300 mg/kg)  -Inhibited NO (56.74% at 10 μg/mL)  - Activity against A549 (IC_50_ 2.29 μg/mL); CT26 (IC_50_ 6.76 μg/mL); HeLa (IC_50_ 23.79 μg/mL); MCF7 (IC_50_ 6.57 μg/mL); J774A.1 (IC_50_ 29.91 μg/mL).  -Activity against *Spodoptera frugiperda*  (LV_50_ 128.8 μg/mL)  -Activity against *S. frugiperda*  (LV_50_ 153.1 μg/mL)  -Acute toxicity in CD-1 mice > 625 mg/kg  -Antidiarrheal in male CD-1 mice  (12.5, 25 mg/kg).  *-In vitro* Ileum contractions from Wistar rat (reduced 68% of contractions at 100 μg/mL)  -Weight of intestinal content  (Inhibition of 60% at 25 mg/kg)  -Inhibited NO (32.85% at 20 μg/mL)  -Inhibited NO (52.37% at 25 μg/mL)  -Cytotoxicity activity: K562 (IC_50_ 17.0 μM); MCF-7 (IC_50_ 28.7 μM)  -Inhibited NO (61.66% at 25 μg/mL)  - Activity against K562 (IC_50_ 31.2 μM); MCF-7 (IC_50_ 33.24 μM)  - Activity against A549 (IC_50_ 5.11 μg/mL); CT26 (IC_50_ 6.17 μg/mL); HeLa (IC_50_ 3.2 μg/mL); MCF7 (IC_50_ 14.87 μg/mL); J774A.1 (IC_50_ 8.81 μg/mL)  - Activity against U251 (IC_50_ 0.27 μM); SKLU-1 (IC_50_ 0.46 μM); COS-7 (IC_50_ 0.61 μM)  -Activity against: U251 (IC_50_ 1.4 μM); SKLU-1 (IC_50_ 0.82 μM); COS-7 (IC_50_ 1.62 μM)  -Anti-inflammatory effect in female CD-1 mice (37.42% inhibition at 1.0 μmol/ear). TPA-induced edema  -Anti-inflammatory effect in female CD-1 mice (25.37 % inhibition at 1.0 μmol/ear) TPA-induced edema  -DPPH (IC_50_ 98.4 μM) | n.i.  Lorke method  Indomethacin 2 mg/ear  Cisplatine (1mg/kg)  n.i.  Cisplatine (IC_50_ 4.6 μg/mL; IC_50_ 2.8 μg/mL; IC_50_ 1.06 μg/mL; IC_50_ 2,.14 μg/mL; IC_50_ 2.45 μg/mL)  n.i.  n.i.  OECD-420  Loperamide (2.5 mg/kg)  Indomethacin (60.41% at 17.1 μg/mL  Indomethacin (60.41% at 17.1 μg/mL).  Adriamycin (IC_50_ 0.20 μM; IC_50_ 0.23 μM)  Indomethacin (60.41% at 17.1 μg/mL)  Adriamycin (IC_50_ 0.20 μM; IC_50_ 0.23 μM)  Cisplatine (IC_50_ 4.6 μg/mL; IC_50_ 2.8 μg/mL; IC_50_ 1.06 μg/mL; IC_50_ 2.14 μg/mL; IC_50_ 2.45 μg/mL)  Adriamycin (IC_50_ 0.08 μM; IC_50_ 0.05 μM; IC_50_ 0.25 μM)  Indomethacin 78.76% inhibition  α-tocopherol (IC_50_ 31.7 μM) | Zavala-Sánchez et al. 2013  Campos-Xolalpa et al. 2021  Cardenas-Ortega et al. 2015  Pérez-Gutiérrez et al. 2013  Campos-Xolalpa et al. 2021  Esquivel et al. 2017  Campos-Xolalpa et al. 2021  Esquivel et al. 2017  Campos-Xopalpa et al. 2017  Esquivel et al. 2017 |
| *S. buchananii* Hedge | Dichloromethane extract  *ent*-19-O-acetoxy-15,16-epoxy-3,13(16), 14-clerodatrien-6,18-diol;  *ent*-15,16-epoxy-10,6-dihydroxycleroda-3,7,13(16),14-tetraene-17,12;18,19-diolide; 7α,12α-dihydroxyhautriwaic acid-19-lactone; *ent*-19-O-acetoxy-15,16-epoxy-3,13(16), 14-clerodatrien-6,8,12-triol.  Dichloromethane extract  Hyptadienic acid  Salvibuchanic acid  Essential oil | Aerial parts  Roots | -Activity against *Staphylococcus aureus*, *S. epidermidis, Streptococcus agalactiae, Enterococcus faecium, E. faecalis* (MIC values >128 μg/mL)  -Antibacterial activity: *Enterococcus faecium* and *E. faecalis* (MIC values >128 μg/mL).  -DPPH (IC_50_ 295 mg/mL); 5-LOX (IC_50_ 0.19 µg/mL)  -Activity against: Jurkat (IC_50_ 30 μM); HeLa (IC_50_ 25 μM)  Jurkat (IC_50_ 38 μM); HeLa (IC_50_ 40 μM)  -Antioxidant, β-carotene-linoleic acid test  (50.27% at 2 mg/mL)  -Antibacterial against *E. coli*, *P. aeruginosa*, *P. pneumonia*, *S. sonne*, *S. Heidelberg*, *S. aureus* (MIC 40-80 μg/mL)  -Anticholinesterase against acetylcholinesterase and butyrylcholinesterase. Ellman's method | Penicillin (*S. epidermidis* and *S. aureus* 1 μg/mL)  Oxacillin sodium salt (*S. epidermidis* and *S. aureus* 6 μg/mL)  BHT: DPPH (IC_50_ 87 mg/mL)  5-LOX (IC_50_ 3.86 µg/mL)  Etoposide: Jurkat (IC_50_ 2.5 μM); HeLa (IC_50_ 4.0 μM).  n.i.  n.i.  Tacrine | Bisio et al. 2015  Giamperi et al. 2012  Beladjila et al. 2018a  Beladjila et al. 2018b |
| *S. cacaliifolia* Benth. | Dichloromethane extract | Aerial parts | -DPPH (IC_50_ 17.0 mg/mL); 5-LOX (IC_50_ 3.50 µg/mL) | BHT: DPPH (IC_50_ 87 mg/mL); 5-LOX (IC_50_ 3.86 µg/mL) | Giamperi et al. 2012 |
| *S. chamaedryoides* Cav. | Dichloromethane extract  (1R,5S,7S,8S,9R,10R,12R)-1,7,8-trihydroxycleroda-3,13(16),14-triene-17,12;18,19-diolide  Tilifodiolide; Splendidin C; Galdosol; (5S,7R,8S,9R,10S,12R)-7,8-dihydroxycleroda-3,13(16),14-triene-17,12;18,19-diolide; (8S,9R,10S,12R)-7,8-dihydroxycleroda-3,13(16),14-triene-17,12;18,19-diolide;  (7R,8S,9R,12R)-7-hydroxy-5,10-seco-neo-cleroda-1(10),2,4,13(16),14-pentaene-17,12;18,19-diolide; (5R,7R,8S,9R,10R,12R)-7-hydroxycleroda-1,3,13(16),14-tetraene-7,12;18,19-diolide; (5S,7R,8R,9R,10S,12R)-7,8-dihydroxycleroda-3,13(16),14-triene-17,12;18,19-diolide  Dichloromethane extract | Aerial parts | -Antidiabetic: Inhibited α-glucosidase  (IC_50_ 55.8 μg/mL) and α-amylase (IC_50_ 121 μg/mL)  - Activity against *Enterococcus faecium*, *E. faecalis* MIC (2-4 μg/mL)  -Inhibited α-glucosidase (IC_50_ 113 μg/mL) and α-amylase (IC_50_ 238 μg/mL)  -MIC (2-4 μg/mL) against  *Enterococcus faecium* and *E. faecalis*  -DPPH (IC_50_ 98 mg/mL); 5-LOX (IC_50_ 0.88 µg/mL) | Acarbose (IC_50_ 0.1 μM)  Methicillin-resistant and vancomycin-resistant MIC (>128 μg/mL)  Acarbose (IC_50_ 0.1 μM)  Methicillin-resistant and vancomycin-resistant MIC (>128 μg/mL)  BHT: DPPH (IC_50_ 87 mg/mL); 5-LOX (IC_50_ 3.86 µg/mL) | Bisio et al. 2017  Giamperi et al. 2012 |
| *S. cinnabarina* M. Martens & Galeotti | 3,4-secoisopimara-4(18),7,15-triene-3-oic acid  Sodium salt 3,4-secoisopimara-4(18),7,15-triene-3-oic acid | Aerial parts | -Antispasmodic effect (IC_50_ 2.7 µg/mL)  -Hypotensive effect in male Wistar rats  (3 mg/kg i.v.). Ganglion-blockade  -Anxiolytic effect in male CD-1 mice  (10 mg/kg i.p.). Elevated plus-maze test  -Sedative effect in male CD-1 mice  (10 mg/kg i.p.)  -Anticlastogenic effect (74 % at 33 µM) | Papaverine IC_50_ 3.4 µg/mL  n.i.  Diazepam (2 mg/kg)  Pentobarbital sodium; diazepam (2 mg/kg)  Human lymphocytes | Romussi et al. 2000  Alfieri et al. 2007  Maione et al. 2009  Di Sotto et al. 2012 |
| *S. clinopodioides* Kunth | Clinopodiolide B  Clinopodiolide C  Clinopodiolide A-C; 19-O-Acetylclinopodiolide A;  triacetylclinopodiolide B  Clinopodiolide A-C;  triacetylclinopodiolide B | Aerial parts | -DPPH (IC_50_ 24.8 µM); TBARS (IC_50_ 5.9 µM)  -DPPH (IC_50_ 29.9 µM); TBARS (IC_50_ 2.7 µM)  -Activity against *Entamoeba histolytica* (IC_50_ 31.3-48.5 µM)  *-Giardia lamblia* (IC_50_ 46.4-67.1 μM)  -Antidiarrheal male Sprague-Dawley rats (0.01-40 mg/kg, p.o.), (ID_50_ 2.2-5.5 μmol/kg) | Quercetin: DPPH (IC_50_ 10.9 µM); TBARS (IC_50_ 1.5 µM);  BHT: TBARS (IC_50_ 1.2 µM); α-tocopherol: DPPH (IC_50_ 31.7 µM); TBARS (IC_50_ 6.8 µM)  Emetine (IC_50_ 2.2 µM);  Metronidazole (IC_50_ 0.2 µM)  Emetine (IC_50_ 0.8 µM); Metronidazole (IC_50_ 1.2 µM)  Quercetin (ID_50_ 1.1 μmol/kg); Loperamide hydrochloride (ID_50_ 0.2 μmol/kg) | Busto-Brito et al. 2019 |
| *S. coccinea* Buc’hoz ex Etl. | Aqueous extract | Leaves | -DPPH: 80 % inhibition at 160 µg/mL  -Antidiabetic. Albino Wistar rats  (150–200 g) | Ascorbic acid  Glibenclamide (10 mg/kg) | Sudaramoorthy et al. 2021 |
| *S. connivens* Epling | Chloroform extract  Dichloromethane extract  Mixture oleanolic and ursolic acid  Eupatorin  Nuchensin  Methanol extract | Aerial parts | -Activity against *Spodoptera frugiperda* (LV_50_ 936 ppm)  -Anti-inflammatory in male CD-1 mice (60.55 % inhibition at 2.0 mg/ear). TPA- induced edema  -TPA (57.20 % inhibition)  -TPA (56.40 % inhibition)  -*Entamoeba histolytica* (IC_50_ 0.072 µM)  - *Giardia lamblia* (IC_50_ 0.118 μM)  -Antidiarrheal in male CD-1 mice  (6.25-200 mg/kg p.o.) | n.i.  Indomethacin (64.5 % inhibition)  Metronidazole *E. histolytica* (IC_50_ 0.23 µM)  *Giardia lamblia* (IC_50_ 1.22 µM); Emetine *E. histolytica* (IC_50_ 2.18 µM)  *Giardia lamblia* (IC_50_ 0.83 µM)  Loperamide (2.5 mg/kg) | Zavala-Sánchez et al. 2013  González-Chávez 2017  Bautista et al. 2020  Pérez et al. 2014 |
| *S. decora* Epling | Acetone extract, fractions;  7β-acetoxysalvimicrophyllin A, Ursolic acid | Aerial parts | -Inhibitory activity of hPTP1B_1−400_ (human protein tyrosine phosphatase) and α-glucosidase | n.i. | Rivera-Chávez et al. 2020 |
| *S. divinorum* Epling & Játiva | Hexane, ethyl acetate and methanol extracts  Mixture of salvinorins  Ethyl acetate extract  Salvinorin A  Fresh leaves | Leaves  Aerial parts  Aerial parts | -Antinociceptive in male and female Swiss albino mice (100-400 mg/kg, p.o.; 3-300 mg/kg, i.p.). Writhing and formalin tests  -Writhing and formalin tests (30 mg/kg, i.p.)  -Neuropathic and inflammatory pain models in rats (30-200 mg/kg)  - Activity against *Entamoeba histolytica* (IC_50_ 49.0 µM); *Giardia lamblia* (IC_50_ 64.8 μM)  -Anxiolytic and antidepressant. Mice (0.000001-1.0 mg/kg, s.c.)  -Antinociceptive and anti-inflammatory. Formalin test. Male CD-1 mice (0.5-2 mg/kg, i.p.)  -Psychoactive effect in human volunteers. Inhalation of the vaporized compound. (200 µg)  -Psychoactive effect in human volunteers. Held in the mouth for 10 minutes without swallowing (30 grams blended with water) | Tramadol (30 mg/kg, i.p.)  Carbamazepine (50 mg/kg); celecoxib (15 mg/kg)  Metronidazole (IC_50_ 0.23 µM); (IC_50_ 1.22 µM)  Emetine (IC_50_ 0.83 µM); (IC_50_ 2.18 µM)  n.i.  n.i.  n.i.  n.i. | Tlacomulco-Flores et al. 2020  Simón-Arceo et al. 2017  Calzada et al. 2015  Casselman et al. 2014  Siebert, 1994 |
| *S. dugesii* Fernald | Dugesin E; Isosalvipuberulin  Dugesin F | Leaves and stems | -Antifeedant against *Pseudaletia separate* larvae. Leaf-disk choice test. Dugesin E (AI 6.49%), isosalvipuberulin (AI 14.94%)  -Activity against influenza virus *in vitro* models (IC_50_ 45.67 μg/mL and 9.43 μg/mL). | AI antifeedant (index, %)  Virazole | Gang et al. 2011 |
| *S. elegans* Vahl | Hydroalcoholic extract  Hydroalcoholic extract  Aqueous extract  Essential oil  Ursolic acid; 5-O-(6-rhamnosylglucoside)-7-hydroxy-4´-methoxyflavanone  Hydroalcoholic extract and fractions (hexane, ethyl acetate, methanol, n-butanol)  Acetate and methanol extracts | Leaves and flowers  Aerial parts      Aerial parts  Leaves and flowers  Leaves  Aerial parts | -Anxiolytic effect in male ICR mice (250-2000 mg/kg, p.o.). Light-dark test  -Antidepressant effect in male ICR mice (125-2000 mg/kg). Forced swimming test  -Antidepressant effect in male Sprague-Dawley rats (3.12-50 mg/kg, i.p). Forced swimming test  -Anxiolytic effect in male Sprague-Dawley rats (3.12-50 mg/kg, i.p.). Elevated plus-maze  Learning and memory by using active and passive avoidance paradigms  -DPPH (EC_50_ 10.7 μg/mL); NO (EC_50_ 91.5 μg/mL); O_2_^-^ (EC_50_ 30.6 μg/mL)  -Antidiabetic and antiobesity:  Inhibited α-glucosidase (EC_50_ 36 μg/mL); pancreatic lipase (EC_50_ 8.2 μg/mL)  -Larvicidal effect against *Aedes albopictus*  (LC_50_ 46.4 ppm)  -Antidepressant effect (15 mg/kg o.p.). Forced swimming test  -Antihypertensive activity *in vivo*, ICR albino mice ICR. Administración de AG II (0.5-2.0 g kg ^-1^, i.v.)  -Antihypertensive activity *in vitro*  Angiotensin I-converting enzyme (ACE) inhibition model. Inhibition of 63% and 77.1% respectively at a concentration of 3 mg/mL.  Effect similar to the positive control | Diazepam (1.0 mg/kg)  Imipramine (10 mg/kg)  Fluoxetine (10 mg/kg)  Imipramine (12.5 mg/kg)  Diazepam (1 mg/kg)  Ascorbic acid (EC_50_ 6.69 μg/mL); Gallic acid (EC_50_ 7.8 μg/mL)  Acarbose: α-glucosidase (EC_50_ 358 μg/mL);  α-amylase (EC_50_ 0.7 μg/mL); pancreatic lipase (EC_50_ 1.8 μg/mL)  n.i.  Imipramide (15 mg/kg)  Losartan (10 mg/kg)  Lisinopril (0.02 mg/mL) | Herrera-Ruiz et al. 2006  Mora et al. 2006  Pereira et al. 2018  Mathew and Thoppil 2011  González-Cortazar et al. 2013  Jiménez-Ferrer et al. 2010  Gutierrez-Román et al. 2021 |
| *S. farinacea* Benth. | Aqueous extract  Salvifarinin B | Aerial parts | -Antibacterial against *Staphylococcus epidermidis* (MIC 8.50 mg/μL; *S. aureus* (MIC 1.06 mg/μL); *Salmonella typhimurium, Escherichia coli, Pseudomonas aeruginosa* (MIC 8.5 mg/μL)  -DPPH (17.4 μg/μL); Ferric reducing power (59.9 μg/μL); TBARS (42.2 μg/μL); β-carotene bleaching inhibition (153.5 μg/μL)  -Anti-inflammatory: NO radical production (EC_50_ 80.8 μg/μL)  -HepG2 (GI_50_ 87.4 μg/μL); HeLA (GI_50_ 77.8 μg/μL); MCF-7 (GI_50_ 59.8 μg/μL); NCI-H460 (GI_50_ 279.5 μg/μL)  -Cytotoxicity to non-tumor cell: PLP2 growth inhibition (GI_50_ 279.5 μg/μL)  -Effect on reducing hepatic steatosis.  Significant reduction the lipid accumulation and the triglyceride level in HL7702 cells | Gram-positive: Nisin (MIC < 0.63 mg/μL)  Gram-negative: (MIC 0.5, 1.0 mg/μL)  Ascorbic acid (6.7 μg/μL)  Butylated hydroxyanisole (16.1 μg/μL); Trolox 23.0 μg/μL; Trolox (41.7 μg/μL)  Dexamethasone (EC_50_ 16.0 μg/μL)  Ellipticine EC_50_ 1.0; 2.0; 1.0.0; 1.0 μg/μL  Model group, curcumin | Afonso et al. 2019a  Fan et al. 2018a |
| *S. greggii* A. Gray | Ethanol extract  Salvigreside D  Aqueous extract  Essential oil | Leaves  Aerial parts  Leaves | -*Staphylococcus aureus* (12 μg/μL). Halo of inhibition (1 mm)  -*Bacillus subtilis* (8 μg/disk)  -DPPH (EC_50_ 21 μg/mL); NO (EC_50_ 167 μg/mL); O_2_^-^ (EC_50_ 62 μg/mL)  -Antidiabetic and antiobesity activities:  Inhibited α-glucosidase (EC_50_ 345 μg/mL); α-amylase (EC_50_ 6.5 μg/mL); pancreatic lipase (EC_50_ 14 μg/mL)  MCF-7 (IC_50_ 35.35 μg/mL); HCT116 (IC_50_ 23.57 μg/mL) | Ampicillin 10 μg; cefotaxime 30 μg; vancomycin 30 μg  n.i.  Ascorbic acid (EC_50_ 6.69 μg/mL)  Gallic acid (EC_50_ 7.8 μg/mL)  Acarbose: α-glucosidase (EC_50_ 358 μg/mL); α-amylase (EC_50_ 0.7 μg/mL); pancreatic lipase (EC_50_ 1.8 μg/mL)  Doxorubicin: MCF-7 (IC_50_ 5.66 μg/mL); HCT116 (IC_50_ 10.84 μg/mL) | Romero et al. 2005  Kawahara et al. 2004  Pereira et al. 2018  Abaza et al. 2021 |
| *S. herbacea* Benth. | Dehydrosalviarin  1β,10β-epoxysalviarin  Tehuanines D-F  Tehuanin H  Tehuanin F  Tehuanin G | Aerial parts | *Entamoeba histolytica* (IC_50_ 210.4 µM)  *Giardia lamblia* (IC_50_ 201.3 μM)  *E. histolytica* (IC_50_ 188.9 µM); *G. lamblia* (IC_50_ 201.3 μM)  *E. histolytica* (IC_50_ 184.8-204.5 µM); *G. lamblia* (IC_50_ 208.6-221.7 μM)  *E. histolytica* (IC_50_ 167.6 µM); *G. lamblia* (IC_50_ 186.4 μM)  U-251 (IC_50_ 41.86 μM); SKLU-1 (IC_50_ 38.92 μM)  TPA-induced ear edema model (IC_50_ 0.24 μM/ear). Male CD-1 mice | Metronidazole (IC_50_ 0.23 µM); (IC_50_ 1.22 µM)  Emetine (IC_50_ 0.83 µM); (IC_50_ 2.18 µM)  Sulforhodamine B  Indomethacin 10 μL/ear | Calzada et al. 2015  Bautista et al. 2012 |
| *S. hirsuta* Jacq. | Micro-phyllandiolide;  Hirsutolides A-D | Leaves and flowers | MDR: Cancer cell line breast (MCF-7)  (RF_MCF-7 sen_ 6-10 at 25 μg/mL)  RF = IC_50_ Vinblastine/IC_50_ Vinblastine in the presence of compound. | Reserpine 5 μg/mL | Toscano et al. 2020 |
| *S. hispanica* L. | Essential oil  Hispanin D, F, I; Hydroxyhautriwaic lactone; Bacchotricuneatin A  Salvihispin A; salvihispin A-2-O-β-D-3-keto-glucopyranoside  Methanol 70% extract  Hexane extract | Aerial part  Seeds  Leaves | -Antifungal activity: *Aspergillus fumigatus*: 88.9 %; *Penicillium expansum*: 84.4 %; *Monilinia laxa:* 80.0% and *Monilinia fructigena:* 78.9%  Insecticidal activity against *Spodoptera exigua* at 150 µg/insect  (LD_50_ 17.49 µg/insect on 3^rd^ instar larvae by topical application, 72 h)  -Protective effect against H_2_O_2_-induced cardiomyocyte injury at 100 µM  Cell viability (85.17 %; 79.80 % and 73.26 %)  -Neurotrophic activity: Enhanced the neurite outgrowth of NGF-mediated PC12 cells at a concentration of 10 μM  -DPPH (SC_50_ 0.30 mg/mL)  -A431 (57.5% inhibition cells) | Potato dextrose agar  (% mycelium growth inhibition)  Acetone  LD_50_ 0.0 µg/insect on 3^rd^ instar larvae by topical application, 72 h  Tanshinone IIA (82.09%)  n.i.  BHA (SC_50_ 0.12 mg/mL)  Cisplatine (10 mg/mL) | Elshafie et al. 2018  Chen et al. 2021  Fan et al. 2019  Fan et al. 2018b  Mitrovic et al. 2021  Janicsák et al. 2011 |
| *S. involucrata* Cav. | Involucratin A  Kingidiol | Aerial parts | -U251 at 25.0 μM (IC_50_ 66.8 μm)  -K562 (IC_50_ 19.0 μM) | Adriamycin 0.5 μM | Bustos-Brito et al. 2021 |
| *S. keerlii* Benth. | Essential oil  Kerlinolide  Chloroform extract | Aerial parts | -Insecticidal activity against *Spodoptera frugiperda* (LC_50_ 590.2 mg/L)  -Antifeedant activity: *Spodoptera littoralis* larvae (AI_50_ less than 100%)  -LD_50_ (3393 mg/kg)  -Antinociceptive. Male CD-1 mice (10-200 mg/kg, p.o). Writhing test  -Anti-inflammatory. TPA (84.96% inhibition)  -*In vitro* antiarthritic. Bovine serum protein denaturation method (IC_50_ 40.73 μg/mL) | n.i.  AI_50_ is the concentration of the compound that gives an estimated antifeedant index of 50%  Lorke  Naproxen (100 mg/kg)  Indomethacin (2.5 μL)  Diclofenac (IC_50_ 19.43 μg/mL) | Zavala-Gómez et al. 2021  Simmonds et al. 1996  Serrano-Vega et al. 2020 |
| *S. leucantha* Cav. | Salvileucantholide  Salvileucantholide; 3β-methoxyisopuberulin; dugesin B  Leucansalvialin G and J  Salvileucalin B  Essential oil  Salviandulin E | Aerial parts | -HCT116 (IC_50_ 32.61 μM); BT474 (IC_50_ 25.02 μM); HepG2 (IC_50_ 37.35 μΜ)  -Acetyl cholinesterase inhibitory activity  (IC_50_ 50.55; 32.2; 22.13 μΜ)  -Neurotrophic activities on PC12 cells  (Differentiation rate 9.52 %)  -Cytotoxic activity against A549 and HT-29 cells with IC_50_ values of 5.23  and 1.88 μg/mL, respectively  -Inhibitory activity of the enzyme  butyrylcholinesterase (IC_50_ 32.60 µg/mL)  Antitrypanosomal activity against *Trypanosoma brucei* (IC_50_ 0.72 µg/mL) | Hsp90 luciferase refolding  n.i.  Nerve Growth Factor  (Differentiation rate 18.43 %)  n.i.  Donepezil  (IC_50_ 3.6 µg/mL)  Pentamidine (IC_50_ 0.0017 µg/mL), suramin ((IC_50_ 1.58 µg/mL), eflornithine (IC_50_ 2.27 µg/mL) | Jiang et al. 2016  Li et al. 2018  Aoyagi et al. 2008  Villalta et al. 2021  (Aoyagi et al. 2014) |
| *S. lineata* Benth. | 1(10)-dehydrosalviarin |  | -Antifeedant activity against *Spodoptera littoralis* larvae (AI_50_ less than 100%) | AI_50_ is the concentration of the compound that gives an estimated antifeedant index of 50% | Simmonds et al. 1996 |
| *S. melissodora* Lag. | 13,14-dihydro-3,4-epoxy-melissodoric acid methyl ester acetate; Acetoxy-7α-hydroxy-*neo*-clerodan-3,13-dien-18,19:16,15-diolide |  | -Antifeedant activity against *Spodoptera littoralis* larvae (AI_50_ less than 100%) | AI_50_ is the concentration of the compound that gives an estimated antifeedant index of 50% | Simmonds et al. 1996 |
| *S. microphylla* Kunth | Carnosic acid 12-methylether  Microphyllandiolide; salvimicrophyllin B; salvimicrophyllin D  Essential oil  Hexane extract  Ethanol extract (95%) | Aerial parts  Leaves  Aerial parts  Leaves | -Antibacterial activity (*S. aureus* 78 µg/mL)  *-E. histolytica* (IC_50_ 182.2; 172.9; 187.2 µM); *G. lamblia* (IC_50_ 201.3; 161.4; 215.3 μM)  -Antioxidant activity β-carotene/linoleic acid (IC_50_ 770 µg/mL)  -Insecticidal against *Spodoptera frugiperda* (LC_50_ 456 ppm)  -Neuroprotective effect in the memory impairment evaluated in male albino rats Step-through passive avoidance (300 mg/kg, p.o.), Morris water maze (150 and 300 mg/kg, p.o.)  -Cholinergic dysfunction via acetyl cholinesterase activity (150 and 300 mg/kg, p.o.)  -Oxidative stress markers: catalase activity, reduced glutathione level and lipid peroxidation level (300 mg/kg, p.o.) | Cefuroxime sodium  Metronidazole (IC_50_ 0.23 µM); (IC_50_ 1.22 µM); Emetine (IC_50_ 0.83 µM); (IC_50_ 2.18 µM)  Thymol (IC_50_ 714 µg/mL)  n.i.  Donezepil (0.5 mg/kg, i.p.) | Aydoğmuş et al. 2006  Calzada et al. 2015  Lima et al. 2012  Romo-Asunción et al. 2016  Ayoub et al. 2022 |
| *S. mexicana* L. | Aqueous extract | Aerial parts | -*Staphylococcus aureus* (MIC/MBC 1.19/1.19 mg/mL). *S. epidermidis* (MIC/MBC 4.75/9.50 mg/mL). *Escherichia coli*, *Pseudomonas aeruginosa* (MIC/MBC 9.50/9.50 mg/mL)  -DPPH, ferric reducing power, TBARS  (EC_50_ 10.0; 34.0; 26.2 µg/mL)  -Anti-inflammatory activity: NO production inhibition  -HepG2 (EC_50_ 52.4 µg/mL); HeLa (EC_50_ 61.0 µg/mL); MCF-7 (EC_50_  66.2 µg/mL) | Nisin: *S. aureus*, *S. epidermidis* (0.63/0.63 mg/mL). *E. coli* (0.50/1.0 mg/mL). *P. aeruginosa* (1.0/1.0 mg/mL)  Ascorbic acid, butylated hydroxy anisole and trolox (EC_50_ 6.68; 16.1; 23.0 µg/mL)  Dexamethasone (EC_50_ 66.3 µg/mL)  Ellipticine (EC_50_ 1.0; 2.0; 1.0 µg/mL) | Afonso et al. 2019b |
| *S. polystachia* Cav. | Linearolactone; polystachyne E  Ethanol extract  Salvifiline A; 15-epi-salvifiline A  Polystachyne G; 15-epi-polystachyne G | Aerial parts  Leaves | -*E. histolytica* (IC_50_ 22.9; 76.6 µM). *G. lamblia* (IC_50_ 28.2; 83.6 μM)  -Neuroprotective: *In vitro* excitotoxicity model (100 % protection at 0.1 µg/mL)  -*In vivo* ischemia model. Male Wistar rats (90.4 % at 3 mg/kg, i.v.)  -Antioxidant. OH (IC_50_ 154.18 μg/mL); O_2_^-^ (IC_50_ 118.97 μg/mL); ROO (IC_50_ 18.21 μg/mL)  -Induced collagen (COL1A1) transcription at 0.27 µM. Human dermal fibroblasts  -Stimulated expression of the elastin gene at 25.6 µM | Metronidazole (IC_50_ 0.23; 1.22 µM); Emetine (IC_50_ 0.83; 2.18 µM)  Resveratrol (40 µM)  Resveratrol: OH (IC_50_ 30.02 μg/mL); O2- (IC_50_ 28.28 μg/mL); ROO (IC_50_ 18.21 μg/mL)  Ascorbic acid (100 µM) | Calzada et al. 2015  Pineda-Ramírez et al. 2020  Bautista et al. 2017 |
| *S. reflexa* Hornem. | Methanol-water extract | Aerial parts | -DPPH (IC_50_ 106.20 μg/mL) | n.i. | Malencic et al. 2000 |
| *S. reptans* Jacq. | 9α-epoxy-7-ketoroyleanone  Horminone | Roots | *-Bacillus cereus* (MIC 2.175 g/mL); *Micrococus luteus* (MIC 2.175 g/mL)  *-B. cereus* (MIC 0.870 g/mL); *M. luteus* (MIC 0.870 g/mL); *S. aureus* (MIC 0.435 g/mL) | Oxytetracycline (MIC 0.93; 0.021 g/mL)  Oxytetracycline (MIC 0.93; 0.021; 0.021 g/mL) | Martínez-Vázquez et al. 1998 |
| *S. rhyacophila* Fernald | Salviarin; 6β-hydroxysalviarin |  | -Antifeedant activity: *Spodoptera littoralis* larvae (AI_50_ less than 100%) | AI_50_ is the concentration of the compound that gives an estimated antifeedant index of 50% | Simmonds et al. 1996 |
| *S. semiatrata* Zucc. | Hexane, ethyl acetate and methanol extracts  7-keto-neoclerodan-3,13-dien-18,19:15,16-diolide  Tilifolidione  Semiatrin | Aerial part  Roots | -Antinociceptive effect in male CD-1 mice (300 mg/kg o.p.). Writhing and formalin tests  -Anxiolytic effect in male CD-1 mice (300 mg/kg o.p.). Open-field, hold-board and plus-maze tests  - Antinociceptive and anxiolytic effects in male CD-1 mice (0.1, 1 and 10 mg/kg)  -U251 (IC_50_ 23.1 µM); K562 (IC_50_ 20.9 μM); HCT15 (IC_50_ 30.2 μM); MCF7 (IC_50_ 18.0 μM); MT2 (IC_50_ 32.8 μM)  -Antifeedant activity: *Spodoptera littoralis* larvae (AI_50_ less than 100%)  -Antifeedant activity: *S. littoralis* | Ketorolac (10 mg/kg, p.o.); diclofenac (10 mg/kg, p.o.)  Clonazepam (0.5 mg/kg, p.o.)  Adriamycin: U251 (IC_50_ 0.1 μM); K562 (IC_50_ 0.3 μM); HCT15 (IC_50_ 0.2 μM); MCF7 (IC_50_ 0.1 μM)  AI_50_ is the concentration of the compound that gives an estimated antifeedant index of 50% | Ortiz-Mendoza et al. 2020  Esquivel et al. 2005  Simmonds et al. 1996 |
| *S. sessei* Benth. | Hexane extract  Dichloromethane extract  Methanolic extract  Isosessein  Sessein | Aerial parts | -*Staphylococcus hominis* (MIC 12.5 μg/mL)  -TPA model. Male ICR mice 25-30 g (0.125-2.0 mg/ear). ED_50_ 40.5%  -FRAP (IC_50_ 1038.19 μg/mL)  -*Staphylococcus haemolyticus*, *S. hominis*,  *Enterococcus faecalis* (MIC 100 μg/mL)  -TPA (ED_50_ 56.0%)  -DPPH (IC_50_ 3313.39 μg/mL); ABTS (IC_50_ 479 μg/mL); FRAP (IC_50_ 1377.08 μg/mL)  -*S. haemolyticus* (MIC 50 μg/mL); *E. faecalis* (MIC 12.5 μg/mL)  -TPA (ED_50_ 66.0%)  -DPPH (IC_50_ 5156.73 μg/mL); ABTS (IC_50_ 1.34 μg/mL); FRAP (IC_50_ 1723.75 μg/mL)  -*S. haemolyticus*, *E. faecalis*, *E. coli*  (MIC 12.5 μg/mL)  -TPA (ED_50_ 79.85%)  -DPPH (IC_50_ 470.9 μg/mL); ABTS (IC_50_ 0.46 μg/mL); FRAP (IC_50_ 1536 μg/mL)  -*S. aureus*, *S. haemolyticus*, *S. epidermidis*, *Streptococcus pyogenes* (MIC 100 μg/mL)  -TPA (ED_50_ 54.36%)  -DPPH (IC_50_ 1458.47 μg/mL); ABTS (IC_50_ 0.61 μg/mL); FRAP (IC_50_ 1034.03 μg/mL) | Gentamicin (MIC 43.14 μM)  Indomethacin (1.0 mg/ear)  Trolox: DPPH (IC_50_ 531.2 μg/mL); ABTS (IC_50_ 0.84 μg/mL); FRAP (IC_50_ 2184.65 μg/mL)  Gentamicin (MIC 43.14 μM) | Gómez-Rivera et al. 2018 |
| *S. shannonii* Donn. Sm. | Sepulturin A, C, E; Infuscatin;  Tehuanin G | Leaves | *-Entamoeba histolytica* (IC_50_ 56.8-79.7 μg/mL)  *-Giardia lamblia* (IC_50_ 63.3-86.4 μg/mL) | (+)-catechin: *E. histolytica* (IC_50_ 65.6 μg/mL); *G. lamblia* (IC_50_ 33.9 μg/mL)  Tyramine: *E. histolytica* (IC_50_ 54.2 μg/mL); *G. lamblia* (IC_50_ 68.9 μg/mL) | Bautista et al. 2013 |
| *S. tiliifolia* Vahl | Tilifodiolide  Tiliifolin E | Aerial parts | -Antifeedant: *Spodoptera littoralis* larvae (AI_50_ less than 100%)  -Antidiarrheal activity in male Balb/c mice  (ED_50_ 10.62 mg/kg p.o.). Castor oil tests  -Vasorelaxant activity smooth muscle tissues from rats (EC_50_ 48 μM)  -Anxiolytic effect in male Balb/c mice  (ED_50_ 20 mg/kg p.o.). Cylinder exploratory test  -Antidepressant effect in male Balb/c mice  (44% action at 50 mg/kg p.o.). Tail suspension test  -Anti-inflammatory activity in male Balb/c mice (200 mg/kg p.o.). Carrageenan-induced paw edema  -Antinociceptive activity male Balb/c mice (ED_50_ 48.2 mg/kg p.o., 1st phase) and (ED_50_ 28.9 mg/kg p.o., 2nd phase). Formalina test  -Anti-nociceptive activity male Balb/c mice (ED_50_ 32.3 mg/kg p.o.). Writhing test  -Neuroprotective activity in PC12 cells  differentiation rate of 11.10% at 10 µM | AI_50_ is the concentration of the compound that gives an estimated antifeedant index of 50%  Loperamide (2.5 mg/kg)  Carbachol  Clonazepam (1.5 mg/kg)  Fluoxetine (20 mg/kg)  Indomethacin (10 mg/kg)  Naproxen (ED_50_ 36.2 mg/kg)  NGF (50 ng/mL) | Simmonds et al. 1996  Alba-Betancourt et al. 2019  González-Chávez et al. 2018  Fan et al. 2017 |
| *S. urica* Epling | Methanol extract | Aerial parts | -Inhibitory effect on the production of verotoxin by enterohemorrhagic *Escherichia coli* O157:H7 (EHEC) (31.3-125 µg/mL) | n.i. | Sakagami et al. 2001 |
| *S. wagneriana* Pol. | Dichloromethane extract | Aerial parts | -DPPH (IC_50_ 636 mg/mL); 5-LOX (IC_50_ 0.11 μg/mL) | BHT: DPPH (IC_50_ 87 mg/mL); 5-LOX IC_50_ (3.86 μg/mL | Giamperi et al. 2012 |

Abbreviations: BHA: 2-terbutyl-hydroxyanisole; BHT: 2-terbutyl-hydroytoluene; DPPH: 2,2-diphenyl-1-picrilhidrazil; EC_50_: Half-maximal effective concentration; FRAP: Ferric ion reducing power; IC_50_: Half-maximal inhibitory concentration; LC_50_: Half-maximal lethal concentration; 5-LOX: lypoxigenase; MDR: Multidrug resistance; MBC: Minimal bactericidal concentration; MIC: Minimum inhibitory concentration; NFG: Neuronal growth factor n.i.: non-information; OECD: Organization for Economic Co-operation and Development; TBA: Thiobarbituric acid; TBARS: Thiobarbituric acid reactive substance.

**References**

Abaza, I., Aboalhaija, N., Alsalman, A., Talib, W., and Afifi, F. (2021). Aroma profile, chemical composition and antiproliferative activity of the hydrodistilled essential oil of a rare *Salvia* species (*Salvia greggii*). *J. Biol. Act. Prod. Nat.* 11, 129–137. doi:10.1080/22311866.2021.1906320.

Afonso, A. F., Pereira, O. R., Fernandes, Â. S. F., Calhelha, R. C., Silva, A. M. S., Ferreira, I. C. F. R., et al. (2019a). The health-benefits and phytochemical profile of *Salvia apiana* and *Salvia farinacea* var. *victoria* blue decoctions. *Antioxidants* 8, 1-14. doi:10.3390/antiox8080241.

Afonso, A. F., Pereira, O. R., Fernandes, Â., Calhelha, R. C., Silva, A. M. S., Ferreira, R. C. F., et al. (2019b). Phytochemical composition and bioactive effects of *Salvia africana*, *Salvia officinalis* “Icterina” and *Salvia mexicana* aqueous Extracts. *Molecules* 24, 4327. doi:10.3390/molecules24234327. PAGINAS

Alba-Betancourt, C., Sánchez-Recillas, A., Alonso-Castro, A. J., Esquivel-Juárez, D., Zapata-Morales, J. R., Yáñez-Pérez, V., et al. (2019). Antidiarrheal, vasorelaxant, and neuropharmacological actions of the diterpene tilifodiolide. *Drug Dev. Res.* 80, 981–991. doi:10.1002/ddr.21578.

Alfieri, A., Maione, F., Bisio, A., Romussi, G., Mascolo, N., and Cicala, C. (2007). Effect of a diterpenoid from *Salvia cinnabarina* on arterial blood pressure in rats. *Phytother. Res* 21, 690–692. doi:10.1002/ptr.

Aoyagi, Y., Yamazaki, A., Nakatsugawa, C., Fukaya, H., Takeya, K., Kawauchi, S., et al. (2008). Salvileucalin B, a novel diterpenoid with an unprecedented rearranged neoclerodane skeleton from *Salvia leucantha* Cav. *Org. Lett.* 10, 4429–4432. doi:10.1021/ol801620u.

Aoyagi, Y., Fujiwara, K., Yamazaki, A., Sugawara, N., Yano, R., Fukaya, H. et al. (2014). Semisynthesis of salviandulin e analogues and their antitrypanosomal activity. *Bioorganic Med. Chem. Lett.* 24, 442–446. doi.org/10.1016/j.bmcl.2013.12.052.

Aydoǧmuş, Z., Yeşilyurt, V., and Topcu, G. (2006). Constituents of *Salvia microphylla*. *Nat. Prod. Res.* 20, 775–781. doi:10.1080/14786410500462843.

Ayoub, I.M., George, M.Y., Menze, E.T., Mahmoud, M., Botros, M., Essam, M., et al. (2022). Insights into the neuroprotective effects of *Salvia officinalis* L. and *Salvia microphylla* Kunth in the memory impairment rat model. *Food Funct.* 13, 2253-2268. doi: 10.1039/d1fo02988f. PMID: 35137748.

Bautista, E., Calzada, F., Yépez-Mulia, L., Bedolla-García, B. Y., Fragoso-Serrano, M., Pastor-Palacios, G., et al. (2020). *Salvia connivens*, a source of bioactive flavones with amoebicidal and giardicidal activity. *Rev Bras Farmacogn.* 30, 729–732. doi:10.1007/s43450-020-00103-8.

Bautista, E., Ortiz-Pastrana, N., Pastor-Palacios, G., Montoya-Contreras, A., Toscano, R. A., Morales-Jiménez, J., et al. (2017). Neo-clerodane diterpenoids from *Salvia polystachya* stimulate the expression of extracellular matrix components in human dermal fibroblasts.  *J. Nat. Prod.* 80, 3003–3009. doi:10.1021/acs.jnatprod.7b00591.

Bautista, E., Fragoso-Serrano, M., Ortiz-Pastrana, N., Toscano, R. A., and Ortega, A. (2016). Structural elucidation and evaluation of multidrug-resistance modulatory capability of amarissinins A–C, diterpenes derived from *Salvia amarissima*. *Fitoterapia* 114, 1–6. doi:10.1016/j.fitote.2016.08.007.

Bautista, E., Fragoso-Serrano, M., Toscano, R. A., García-Peña, M. D. R., and Ortega, A. (2015). Teotihuacanin, a diterpene with an unusual spiro-10/6 system from *Salvia amarissima* with potent modulatory activity of multidrug resistance in cancer cells. Org. Lett. 17, 3280–3282. doi:10.1021/acs.orglett.5b01320.

Bautista, E., Toscano, A., Calzada, F., Díaz, E., Yépez-Mulia, L., and Ortega, A. (2013). Hydroxyclerodanes from *Salvia shannoni*. *J. Nat. Prod.* 76, 1970–1975. doi:10.1021/np400606g.

Bautista, E., Maldonado, E., and Ortega, A. (2012). Neo-clerodane diterpenes from *Salvia herbacea*. *J. Nat. Prod*. 75, 951–958. doi:10.1021/np3001464.

Beladjila, K. A., Berrehal, D., Al-Aboudi, A., Semra, Z., Al-Jaber, H., Bachari, K., et al. (2018a). Composition and antioxidant, anticholinesterase, and antibacterial activities of the essential oil of *Salvia buchananii* from Algeria. *Chem. Nat. Compd.* 54, 581–583. doi:10.1007/s10600-018-2414-z.

Beladjila, K. A., Cotugno, R., Berrehal, D., Kabouche, Z., de Tommasi, N., Braca, A., et al. (2018b). Cytotoxic triterpenes from *Salvia buchananii* roots. *Nat. Prod. Res.* 32, 2025–2030. doi:10.1080/14786419.2017.1365072.

Bisio, A., de Mieri, M., Milella, L., Schito, A. M., Parricchi, A., Russo, D., et al. (2017). Antibacterial and hypoglycemic diterpenoids from *Salvia chamaedryoides*. *J. Nat. Prod.* 80, 503–514. doi:10.1021/acs.jnatprod.6b01053.

Bisio, A., Schito, A. M., Ebrahimi, S. N., Hamburger, M., Mele, G., Piatti, G., et al. (2015). Antibacterial compounds from *Salvia adenophora* Fernald (Lamiaceae). *Phytochemistry* 110, 120–132. doi:10.1016/j.phytochem.2014.10.033.

Bustos-Brito, C., Joseph-Nathan, P., Burgueño-Tapia, E., Martínez-Otero, D., Nieto-Camacho, A., Calzada, F., et al. (2019). Structure and absolute configuration of abietane diterpenoids from *Salvia clinopodioides*: antioxidant, antiprotozoal, and antipropulsive activities. *J. Nat. Prod.* 82, 1207–1216. doi:10.1021/acs.jnatprod.8b00952.

Bustos-Brito, C., Pérez-Juanchi, D., Rivera-Chávez, J., Hernández-Herrera, A. D., Bedolla-García, B. Y., Zamudio, S., et al. (2021). Clerodane and 5 10-seco-clerodane-type diterpenoids from *Salvia involucrata*. *J. Mol. Struct.* 1237, 130367. doi:10.1016/j.molstruc.2021.130367.

Calzada, F., Bautista, E., Barbosa, E., Salazar-Olivo, L. A., Alvidrez-Armendáriz, E., and Yepez-Mulia, L. (2020). Antiprotozoal activity of secondary metabolites from *Salvia circinata*. *Rev Bras Farmacogn.* 30, 593–596. doi:10.1007/s43450-020-00077-7/Published.

Calzada, F., Bautista, E., Yépez-Mulia, L., García-Hernández, N., and Ortega, A. (2015). Antiamoebic and antigiardial activity of clerodane diterpenes from Mexican *Salvia* species used for the treatment of diarrhea. *Phytother Res.* 29, 1600–1604. doi:10.1002/ptr.5421.

Campos-Xolalpa, N., Alonso-Castro, Á. J., Ortíz-Sánchez, E., Zapata-Morales, J. R., González-Chávez, M. M., and Pérez, S. (2021). Anti-inflammatory and antitumor activities of the chloroform extract and anti-inflammatory effect of the three diterpenes isolated from *Salvia ballotiflora* Benth. *BMC complement. med. ther.* 21. doi:10.1186/s12906-020-03179-w.

Campos-Xolalpa, N., Alonso-Castro, Á. J., Sánchez-Mendoza, E., Zavala-Sánchez, M. Á., and Pérez-Gutiérrez, S. (2017). Cytotoxic activity of the chloroform extract and four diterpenes isolated from *Salvia ballotiflora*. *Rev Bras Farmacogn.* 27, 302–305. doi:10.1016/j.bjp.2017.01.007.

Cárdenas-Ortega, N. C., González-Chávez, M. M., Figueroa-Brito, R., Flores-Macías, A., Romo-Asunción, D., Martínez-González, D. E., et al. (2015). Composition of the essential oil of *Salvia ballotiflora* (Lamiaceae) and its insecticidal activity. *Molecules* 20, 8048–8059. doi:10.3390/molecules20058048.

Casselman, I., Nock, C. J., Wohlmuth, H., Weatherby, R. P., and Heinrich, M. (2014). From local to global - Fifty years of research on *Salvia divinorum*. *J. Ethnopharmacol.* 151, 768–783. doi:10.1016/j.jep.2013.11.032.

Chen, Y., Luo, J., Zhang, N., Yu, W., Jiang, J., and Dai, G. (2021). Insecticidal activities of *Salvia hispanica* L. essential oil and combinations of their main compounds against the beet armyworm *Spodoptera exigua*. *Ind Crops Prod.* 162, 113271. doi:10.1016/j.indcrop.2021.113271.

di Sotto, A., Carbone, F., Hrelia, P., Maffei, F., Castelli, F., Sarpietro, M. G., et al. (2012). Anticlastogenic effect in human lymphocytes by the sodium Salt of 3,4-secoisopimar-4(18),7,15-trien-3-oic acid. *J. Nat. Prod.* 75, 1294–1298. doi:10.1021/np3001893.

Elshafie, H. S., Aliberti, L., Amato, M., de Feo, V., and Camele, I. (2018). Chemical composition and antimicrobial activity of chia (*Salvia hispanica* L.) essential oil. *Eur. Food Res. Technol.* 244, 1675–1682. doi:10.1007/s00217-018-3080-x.

Esquivel, B., Bustos-Brito, C., Sánchez-Castellanos, M., Nieto-Camacho, A., Ramírez-Apan, T., Joseph-Nathan, P., et al. (2017). Structure, absolute configuration, & antiproliferative activity of abietane & icetexane diterpenoids from *Salvia ballotiflora*. *Molecules* 22, 1690. doi:10.3390/molecules22101690.

Esquivel, B., Sánchez, A. A., Vergara, F., Matus, W., Hernandez-Ortega, S., and Ramírez-Apan, T. M. (2005). Abietane diterpenoids from the roots of some Mexican *Salvia* species (Labiatae): Chemical diversity, phytogeographical significance, and cytotoxic activity. *Chem. Biodivers.* 2, 738–747.

Fan, M., Luo, D., Peng, L. Y., Li, X. N., Wu, X. de, Ji, X., et al. (2019). Neo-clerodane diterpenoids from aerial parts of *Salvia hispanica* L. and their cardioprotective effects. *Phytochemistry* 166, 1-11. doi:10.1016/j.phytochem.2019.112065.

Fan, M., Chen, X. J., Wu, X. de, Shao, L. D., Ji, X., and Zhao, Q. S. (2018a). Salvifarinin A, a neo-clerodane diterpenoid with a 6/5/7 tricyclic skeleton from *Salvia farinacea*. *Tetrahedron Lett.* 59, 3065–3068. doi:10.1016/j.tetlet.2018.06.015.

Fan, M., Zhu, Y., Zhang, Z. J., Du, R. N., Zhu, Q. F., Wu, X. de, et al. (2018b). Salvihispin A and its glycoside, two neo-clerodane diterpenoids with neurotrophic activities from *Salvia hispanica* L. *Tetrahedron Lett.* 59, 143–146. doi:10.1016/j.tetlet.2017.12.010.

Fan, M., Bao, Y., Zhang, Z. J., Zhang, H. bin, and Zhao, Q. S. (2017). New neo-clerodane diterpenoids with neurotrophic activity from the aerial parts of *Salvia tiliifolia*. *Fitoterapia* 123, 44–50. doi:10.1016/j.fitote.2017.09.013.

Flores-Bocanegra, L., González-Andrade, M., Bye, R., Linares, E., and Mata, R. (2017). α-Glucosidase inhibitors from *Salvia circinata*. *J. Nat. Prod.* 80, 1584–1593. doi:10.1021/acs.jnatprod.7b00155.

Fragoso-Serrano, M., Ortiz-Pastrana, N., Luna-Cruz, N., Toscano, R. A., Alpuche-Solís, A. G., Ortega, A., et al. (2019). Amarisolide F, an acylated diterpenoid glucoside and related terpenoids from *Salvia amarissima*. *J. Nat. Prod.* 82, 631–635. doi:10.1021/acs.jnatprod.8b00565.

Gang, X., Fang, Z., Xian-Wen, Y., Juan, Z., Li-Xin, Y., Shen, X. L., et al. (2011). Neo-Clerodane diterpenoids from *Salvia dugesii* and their bioactive studies. *Nat. prod. bioprospect*. 1, 81–86. doi:10.1007/s13659-011-0016-6.

Giamperi, L., Bucchini, A., Bisio, A., Giacomelli, E., Romussi, G., and Ricci, D. (2012). Total phenolic content and antioxidant activity of *Salvia* spp. exudates. *Nat. Prod. Commun.* 2, 201–202.

Gómez-Rivera, A., González-Cortazar, M., Herrera-Ruíz, M., Zamilpa, A., and Rodríguez-López, V. (2018). Sessein and isosessein with anti-inflammatory, antibacterial and antioxidant activity isolated from *Salvia sessei* Benth. *J. Ethnopharmacol.* 217, 212–219. doi:10.1016/j.jep.2018.02.012.

González-Chávez, M. M., Alonso-Castro, A. J., Zapata-Morales, J. R., Arana-Argáez, V., Torres-Romero, J. C., Medina-Rivera, Y. E., et al. (2018). Anti-inflammatory and antinociceptive effects of tilifodiolide, isolated from *Salvia tiliifolia* Vahl (Lamiaceae). *Drug Dev. Res.* 79, 165–172. doi:10.1002/ddr.21432.

González-Chávez, M. M., Ramos-Velázquez, C. S., Serrano-Vega, R., Pérez-González, C., Sánchez-Mendoza, E., and Pérez-Gutiérrez, S. (2017). Anti-inflammatory activity of standardized dichloromethane extract of *Salvia connivens* on macrophages stimulated by LPS. *Pharm. Biol.* 55, 1467–1472. doi:10.1080/13880209.2017.1305423.

González-Cortazar, M., Maldonado-Abarca, A. M., Jiménez-Ferrer, E., Marquina, S., Ventura-Zapata, E., Zamilpa, A., et al. (2013). Isosakuranetin-5-O-rutinoside: A new flavanone with antidepressant activity isolated from *Salvia elegans* Vahl. *Molecules* 18, 13260–13270. doi:10.3390/molecules181113260.

Gutiérrez-Román, A. S., Gonzalez-Cortazar, M., Trejo-Tapia, G., Herrera-Ruiz, M., Zamilpa, A., Sanchéz-Mendoza, E., et al. (2021). Angiotensin-converting enzyme inhibitors from *Salvia elegans* Vahl. *Nat. Prod. Res.* 35, 5344–5349. doi:10.1080/14786419.2020.1758093.

Herrera-Ruiz, M., García-Beltrán, Y., Mora, S., Díaz-Véliz, G., Viana, G. S. B., Tortoriello, J., et al. (2006). Antidepressant and anxiolytic effects of hydroalcoholic extract from *Salvia elegans*. *J. Ethnopharmacol.* 107, 53–58. doi:10.1016/j.jep.2006.02.003.

Janicsák, G., Zupkó, I., Nikolova, M. T., Forgo, P., Vasas, A., Máthé, I., et al. (2011). Bioactivity-guided study of antiproliferative activities of *Salvia* extracts. *Nat. Prod. Commun.* 6, 575–579.

Jiang, Y. J., Su, J., Shi, X., Wu, X. de, Chen, X. Q., He, J., et al. (2016). Neo-Clerodanes from the aerial parts of *Salvia leucantha*. *Tetrahedron* 72, 5507–5514. doi:10.1016/j.tet.2016.07.037.

Jiménez-Ferrer, E., Hernández Badillo, F., González-Cortazar, M., Tortoriello, J., and Herrera-Ruiz, M. (2010). Antihypertensive activity of *Salvia elegans* Vahl. (Lamiaceae): ACE inhibition and angiotensin II antagonism. *J. Ethnopharmacol.* 130, 340–346. doi:10.1016/j.jep.2010.05.013.

Kawahara, N., Tamura, T., Inoue, M., Hosoe, T., Kawai, K. I., Sekita, S., et al. (2004). Diterpenoid glucosides from *Salvia greggii*. *Phytochemistry* 65, 2577–2581. doi:10.1016/j.phytochem.2004.08.012.

Li, L. W., Qi, Y. Y., Liu, S. X., Wu, X. de, and Zhao, Q. S. (2018). Neo-clerodane and abietane diterpenoids with neurotrophic activities from the aerial parts of *Salvia leucantha* Cav. *Fitoterapia* 127, 367–374. doi:10.1016/j.fitote.2018.03.007.

Lima, R. K., Cardoso, M. D. G., Andrade, M. A., Guimarães, P. L., Batista, L. R., and Nelson, D. L. (2012). Bactericidal and antioxidant activity of essential oils from myristica fragrans houtt and *Salvia microphylla* H.B.K. *J. Am. Oil. Chem. Soc.* 89, 523–528. doi:10.1007/s11746-011-1938-1.

Maione, F., Camela Bonito, M., Colucci, M., Cozzolino, V., Bisio, A., Romussi, G., et al. (2009). First evidence for an anxiolytic effect of a diterpenoid from *Salvia cinnabarina*. *Nat. Prod. Commun.* 4, 469–472.

Malenčic, D., Gašic, O., Popovic, M., and Boža, P. (2000). Screening for Antioxidant Properties of *Salvia reflexa* Hornem. *Phytother Res.* 14, 546–548.

Martínez-Vázquez, M., Miranda, P., Valencia, N. A., Torres, M. L., Miranda, R., Cárdenas, J., et al. (1998). Antimicrobial diterpenes from *Salvia reptans*. *Pharm. Biol.* 36, 77–80. doi:10.1076/phbi.36.2.77.4611.

Mathew, J., and Thoppil, J. E. (2011). Chemical composition and mosquito larvicidal activities of *Salvia* essential oils. *Pharm. Biol.* 49, 456–463. doi:10.3109/13880209.2010.523427.

Mitrović, J., Nikolić, N., Karabegović, I., Lazić, M., and Stojanović, G. (2021). Characterization of free and insoluble-bound phenolics of chia (*Salvia hispanica* L.) seeds. *Nat. Prod. Res.* 36, 385–389. doi:10.1080/14786419.2020.1761357.

Mora, S., Millán, R., Lungenstrass, H., Díaz-Véliz, G., Morán, J. A., Herrera-Ruiz, M., et al. (2006). The hydroalcoholic extract of *Salvia elegans* induces anxiolytic- and antidepressant-like effects in rats. *J. Ethnopharmacol.* 106, 76–81. doi:10.1016/j.jep.2005.12.004.

Moreno-Pérez, F., Hernandez-Leon, A., Valle-Dorado, M. G., Cano-Martínez, A., Narváez-González, F., Aguirre-Hernández, E., et al. (2021). Neo-clerodane diterpenic influence in the antinociceptive and anti-inflammatory properties of *Salvia circinnata* Cav. *J. Ethnopharmacol.* 268, 113550. doi:10.1016/j.jep.2020.113550.

Moreno-Pérez, G. F., González-Trujano, M. E., Martínez-Gordillo, M. J., San Miguel-Chávez, R., Basurto-Peña, F. A., Dorazco-González, A., et al. (2019). Amarisolide A and pedalitin as bioactive compounds in the antinociceptive effects of *Salvia circinata* (Lamiaceae). *Bot. Sci.* 97, 355–365. doi:10.17129/botsci.2187.

Ortiz-Mendoza, N., Zavala-Ocampo, L. M., Martínez-Gordillo, M. J., González-Trujano, M. E., Basurto-Peña, F. A., Bazany-Rodríguez, I. J., et al. (2020). Antinociceptive and anxiolytic-like effects of a neo-clerodane diterpene from *Salvia semiatrata* aerial parts. *Pharm. Biol.* 58, 620–629. doi:10.1080/13880209.2020.1784235.

Pereda-Miranda, R., Lourdes Hernández and Lopez, R. (1992). A novel antimicrobial abietane-type diterpene from *Salvia albocaerulea*. *Planta Medica* *Letters* 58, 223-224. doi: 10.1055/s-2006-961436. PMID: 1529036.

Pereira, O. R., Catarino, M. D., Afonso, A. F., Silva, A. M. S., and Cardoso, S. M. (2018). *Salvia elegans*, *Salvia greggii* and *Salvia officinalis* decoctions: Antioxidant activities and inhibition of carbohydrate and lipid metabolic enzymes. *Molecules* 23, 3169. doi:10.3390/molecules23123169.

Pérez Gutiérrez, S., Zavala Mendoza, D., Soto Peredo, C., Sánchez Sánchez, O., and Zavala Sánchez, M. A. (2014). Evaluation of the anti-diarrheal activity of *Salvia connivens*. *Pharm. Biol.* 52, 1467–1470. doi:10.3109/13880209.2014.898076.

Pérez-Gutiérrez, S., Zavala-Mendoza, D., Hernández-Munive, A., Mendoza-Martínez, Á., Pérez-González, C., and Sánchez-Mendoza, E. (2013). Antidiarrheal activity of 19-deoxyicetexone isolated from *Salvia ballotiflora* benth in mice and rats. *Molecules* 18, 8895–8905. doi:10.3390/molecules18088895.

Pineda-Ramírez, N., Calzada, F., Alquisiras-Burgos, I., Medina-Campos, O. N., Pedraza-Chaverri, J., Ortiz-Plata, A., et al. (2020). Antioxidant properties and protective effects of some species of the Annonaceae, Lamiaceae, and Geraniaceae families against neuronal damage induced by excitotoxicity and cerebral ischemia. *Antioxidants (Basel)* 9, 253. doi:10.3390/antiox9030253.

Rivera-Chávez, J., Bustos-Brito, C., Aguilar-Ramírez, E., Martínez-Otero, D., Rosales-Vázquez, L., Dorazco-González, A., et al. (2020). Hydroxy- *neo*-Clerodanes and 5,10- seco- *neo*-Clerodanes from *Salvia decora*. *J. Nat. Prod.* 83, 2212–2220. doi:10.1021/acs.jnatprod.0c00313.

Romero, C. D., Chopin, S. F., Buck, G., Martinez, E., Garcia, M., and Bixby, L. (2005). Antibacterial properties of common herbal remedies of the southwest. *J. Ethnopharmacol.* 99, 253–257. doi:10.1016/j.jep.2005.02.028.

Romo-Asunción, D., Antonio Ávila-Calderón, M., Ramos-López, M. A., Barranco-Florido, J. E., Rodríguez-Navarro, S., Romero-Gomez, S., et al. (2016). Juvenomimetic and insecticidal activities of Senecio salignus (Asteraceae) and *Salvia microphylla* (Lamiaceae) on *Spodoptera frugiperda* (Lepidoptera: Noctuidae). *Fla. Entomol.* 99, 345–351. doi.org/10.1653/024.099.0301.

Romussi, G., Ciarallo, G., Bisio, A., Fontana, N., de Simone, F., de Tommasi, N., et al. (2000). A new diterpenoid with antispasmodic activity from *Salvia cinnabarina*. *Planta Med.* 67, 153–155. . doi.10.1055/s-2001-11511.

Sakagami, Y., Murata, H., Nakanishi, T., Inatomi, Y., Watabe, K., Iinuma, M., et al. (2011). Inhibitory effect of plant extracts on production of verotoxin by enterohemorrhagic *Escherichia coli* O157 : H7. *J. HEALTH Sci.* 47, 473–477. doi.org/10.1248./jhs.47.473.

Salinas-Arellano, E., Pérez-Vásquez, A., Rivero-Cruz, I., Torres-Colin, R., González-Andrade, M., Rangel-Grimaldo, M., et al. (2020). Flavonoids and terpenoids with PTP-1B inhibitory properties from the infusion of *Salvia amarissima* ortega. *Molecules* 25, 3530. doi:10.3390/molecules25153530.

Serrano-Vega, R., Pérez-González, C., Alonso-Castro, Á., Zapata-Morales, J., and Pérez-Gutiérrez, S. (2020). Anti-inflammatory and antinociceptive activities of *Salvia keerlii*. *Pharmacogn. Mag.* 16, 27-33. doi:10.4103/pm.pm_223_19.

Siebert, D. J. (1994). *Salvia divinorum* and Salvinorin A: new pharmacologic findings. *J. Ethnopharmacol.* 43, 53-56. doi: 10.1016/0378-8741(94)90116-3.

Simmonds, M. S. J., Blaney, W. M., Esquivel, B., and Rodriguez-Hahn, L. (1996). Effect of clerodane-type diterpenoids isolated from *Salvia* spp. on the feeding behavior of *Spodoptera littoralis*. *Pestic. Sci.* 47, 17–23. doi. 1002/(sici)1096-9063(199605)47:13.0.co;2-i.

Simón-Arceo, K., González-Trujano, M. E., Coffeen, U., Fernández-Mas, R., Mercado, F., Almanza, A., et al. (2017). Neuropathic and inflammatory antinociceptive effects and electrocortical changes produced by *Salvia divinorum* in rats. *J. Ethnopharmacol.* 206, 115–124. doi:10.1016/j.jep.2017.05.016.

Solares-Pascasio, J. I., Ceballos, G., Calzada, F., Barbosa, E., and Velazquez, C. (2021). Antihyperglycemic and lipid profile effects of *Salvia amarissima* ortega on streptozocin-induced type 2 diabetic mice. *Molecules* 26, 947. doi:10.3390/molecules26040947.

Sudaramoorthy, A., Shanmugam, G., and Shanmugam, N. (2021). Inhibitory effect of *Salvia coccinea* on inflammatory responses through NF-κB signaling pathways in THP-1 cells and acute rat diabetes mellitus. *Acta Histochem.* 123. 151735. doi:10.1016/j.acthis.2021.151735.

Tlacomulco-Flores, L. L., Déciga-Campos, M., González-Trujano, M. E., Carballo-Villalobos, A. I., and Pellicer, F. (2020). Antinociceptive effects of *Salvia divinorum* and bioactive salvinorins in experimental pain models in mice. *J. Ethnopharmacol.* 248, 112276. doi:10.1016/j.jep.2019.112276.

Toscano, R. A., Cárdenas, J., Ortiz-Pastrana, N., Fragoso-Serrano, M., Ortega, A., Pérez-Vázquez, F. J., et al. (2020). NMR and SC-XRD analyses of a solid solution of diastereomers of microphyllane diterpenoids from *Salvia hirsuta*. *J. Mol. Struct.* 1203, 127409. doi:10.1016/j.molstruc.2019.127409.

Villalta, G., Salinas, M., Calva, J., Bec, N., Larroque, C., Vidari, G., et al. (2021). Selective buche inhibitory activity, chemical composition, and enantiomeric content of the essential oil from *Salvia leucantha* cav. Collected in Ecuador. *Plants* 10, 1169. doi:10.3390/plants10061169.

Zavala-Gómez, C. E., Zamora-Avella, D., Luis Rodríguez-Chávez, J., Zavala-Sánchez, M. Á., Campos-Guillén, J., Moustapha Bah, M., et al. (2021). Bioactivity of 1,8-Cineole and Essential Oil of *Salvia keerlii* (Lamiaceae) against *Spodoptera frugiperda*. *Southwest. Entomol.* 46, 385–396.

Zavala-Sánchez, M. A., Gutiérrez, S. P., Romo-Asunción, D., Cárdenas-Ortega, N. C., and Ramos-López, M. A. (2013). Activity of four *Salvia* species against *Spodoptera frugiperda* (J.E Smith) (Lepidoptera: Noctuidae). *Southwest. Entomol.* 38, 67–74. doi:10.3958/059.038.0107.
